# Contrasting Health Effects of *Bacteroidetes* and *Firmicutes* Lies in Their Genomes: Analysis of P450s, Ferredoxins, and Secondary Metabolite Clusters

**DOI:** 10.3390/ijms23095057

**Published:** 2022-05-02

**Authors:** Bridget Valeria Zinhle Nkosi, Tiara Padayachee, Dominik Gront, David R. Nelson, Khajamohiddin Syed

**Affiliations:** 1 Department of Biochemistry and Microbiology, Faculty of Science and Agriculture, University of Zululand, KwaDlangezwa 3886, South Africa; brilenhle@gmail.com (B.V.Z.N.); teez07padayachee@gmail.com (T.P.); 2 Biological and Chemical Research Center, Faculty of Chemistry, University of Warsaw, Pasteura 1, 02-093 Warsaw, Poland; dgront@gmail.com; 3 Department of Microbiology, Immunology and Biochemistry, University of Tennessee Health Science Center, Memphis, TN 38163, USA

**Keywords:** *Bacteroidetes*, *Firmicutes*, Cytochrome P450 monooxygenases, ferredoxins, secondary metabolite gene clusters, human gut microbiome, human health

## Abstract

Species belonging to the bacterial phyla *Bacteroidetes* and *Firmicutes* represent over 90% of the gastrointestinal microbiota. Changes in the ratio of these two bacterial groups were found to have contrasting health effects, including obesity and inflammatory diseases. Despite the availability of many bacterial genomes, comparative genomic studies on the gene pools of these two bacterial groups concerning cytochrome P450 monooxygenases (P450s), ferredoxins, and secondary metabolite biosynthetic gene clusters (smBGCs) are not reported. This study is aimed to address this research gap. The study revealed the presence of diverse sets of P450s, ferredoxins, and smBGCs in their genomes. *Bacteroidetes* species have the highest number of P450 families, ferredoxin cluster-types, and smBGCs compared to *Firmicutes* species. Only four P450 families, three ferredoxin cluster types, and five smBGCs are commonly shared between these two bacterial groups. Considering the above facts, we propose that the contrasting effects of these two bacterial groups on the host are partly due to the distinct nature of secondary metabolites produced by these organisms. Thus, the cause of the contrasting health effects of these two bacterial groups lies in their gene pools.

## 1. Introduction

The bacterial phylum *Bacteroidetes* consists of gram-negative bacteria, primarily degraders of carbohydrates [1,2]. They provide energy for their host by breaking down the polysaccharides that their host cannot digest [1,2]. These bacterial species can be found in all ecosystems, including fresh water and soil [2,3]. In humans, they are predominantly found in the gastrointestinal tract, inhabiting the distal gut [4]. They play a significant role in the gastrointestinal tract by interacting with the gut immune system, inhibiting the colonization of potential pathogens [5]. Gut *Bacteroidetes* species produce acids, such as acetic acid, propionic acid, and succinic acid, as final products of their metabolism, which help kill the pathogens [5].

This bacterial phylum consists of 130 genera [6]; *Bacteroides* is known for producing skimmed or low-fat milk and displaying postbiotic activities, such as promoting health and well-being [7]. *Zobellia galactanivorans*, a flavobacterium, degrades algal biomass [8]. *Spirosoma* species produce valuable human compounds with neuroprotective, anti-tumorigenic, and anti-inflammatory properties, among others [9]. Some of the secondary metabolites produced by these species and their biological properties are listed in Table 1. In addition to the many beneficial bacteria, this phylum also has pathogenic species. Species belonging to the genus *Empedobacter* are human pathogens. In humans, they cause meningitis, endophthalmitis, urinary tract infection, and bacteremia [10,11]. *Bacteroides fragilis* and other members of the *Bacteroides* genus were shown to be the primary causes of anaerobic septicemia [12]. The genus *Elizabethkingia* consists of novel species such as *E. anophelis* and *E. meningosepta*, which are gram-negative, non-spore-forming, nonmotile, and rod-shaped, and depict natural resistance to multiple known antibiotics [13].

For quite some time, *Bacteroidetes*, in association with other bacterial species belonging to the phylum *Firmicutes*, have been in focus due to their impact on human health. These two bacterial groups represent 90% of the gut microbiota [22], indicating their importance in human health. As expected, due to their abundance in the human gut, the ratio of *Firmicutes* and *Bacteroidetes* was found to have contrasting effects on human health, including in obesity and inflammatory disease [23,24], albeit with some uncertainties [25]. These two bacterial groups produce secondary metabolites that ultimately affect human health.

Despite the availability of many species genomes belonging to these two phyla, comparative analyses of the gene pools responsible for these two bacterial groups’ behavior and health effects on humans are scarcely reported. Genome-wide comparative analysis of a few species revealed that *Firmicutes* species have smaller genomes and a disproportionately smaller number of glycan degrading enzymes than *Bacteroidetes* species [26]. A subsequent study involving 60 *Bacteroidetes* and 197 *Firmicutes* on host-synthesized mucin glycans revealed different glycosyl hydrolases patterns between *Bacteroidetes* and *Firmicutes*, indicating some preference for cleaved mucin glycans in the host [27]. Analysis of chicken caecum gut microbiome revealed that *Bacteroidetes* and *Firmicutes* follow different strategies for colonization and coexistence in the intestinal tract [28]. A study detailing the genomic blueprint of the human gut microbiota revealed a large number of uncultured *Firmicutes* compared to *Bacteroidetes* [29]. The study also reported novel secondary metabolite biosynthetic gene clusters (smBGCs) coding for undiscovered natural compounds produced by the intestinal microbiota [29].

In a recent study, *Firmicutes* species were found to have a large number of cytochrome P450 monooxygenases (CYPs/P450s) [30] and their redox proteins, ferredoxins [31], in their genomes. P450s are heme–thiolate enzymes known to play a role in an organism’s primary and secondary metabolism. These enzymes are found in species across the biological kingdoms, including in nonliving entities such as viruses [32,33]. It is now well-established that the P450 contingent of organisms is indicative of their lifestyle, because the lifestyle of an organism was found to affect the P450 gene pool in its genome [30,34,35,36,37,38,39,40,41,42]. P450s were found to help organisms adapt to ecological niches, and organisms with parasitic, commensal, or adapted living on simple carbon sources were found to have the lowest number of P450s in their genome [30,34,35,36,37,38,39,40,41]. P450s need electrons to perform their enzymatic reactions, and these electrons are supplied by redox proteins such as ferredoxins [43,44]. A recent study revealed many ferredoxins in *Firmicutes* species [31]. The study also suggested unique ferredoxins in *Firmicutes* species indicative of characteristics of the species in this phylum [31]. It is well-known that P450s, due to their regio- and stereo-specific oxidation capabilities, play a crucial role in producing secondary metabolites per se, contributing to the diversity of the secondary metabolites in an organism [45,46]. In a recent study, many P450s were part of smBGCs, indicating their role in secondary metabolite production in *Firmicutes* species [30].

Considering the above facts, especially the P450s and ferredoxins gene pools as characteristics of an organism’s lifestyle and their role in the production of secondary metabolites, in this study, we selected these two sets of genes for comparative analysis of the gene pools of *Bacteroidetes* and *Firmicutes*. These two bacterial groups are known to produce secondary metabolites (Table 1) [30], and it is well-known that these secondary metabolites affect human health [47,48,49]. Thus, we performed a comprehensive comparative analysis of smBGCs to understand the rationale behind these two bacterial groups’ distinct effects on human health regarding their secondary metabolism.

## 2. Results and Discussion

### 2.1. Only a Few Bacteroidetes Species Have P450s

**Figure 1:**
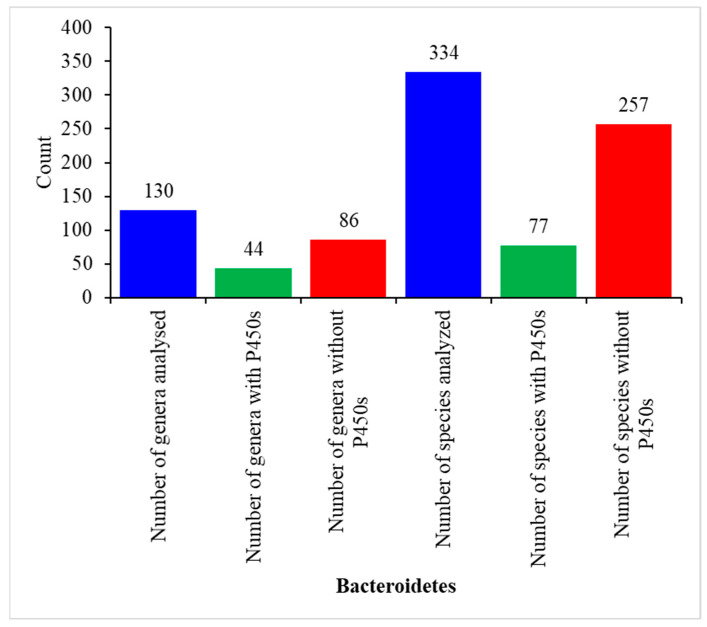
Analysis of P450s in *Bacteroidetes* species. The number next to the bar indicates the count for that category. Detailed analysis of the species, genera, and P450s are presented in Table S1.

Genome-wide analysis of P450s in 334 *Bacteroidetes* species belonging to 130 genera revealed the presence of P450s only in 77 *Bacteroidetes* species, indicating most of the species do not have P450s in their genome (Figure 1 and Table S1). This shows only 23% of *Bacteroidetes* species have P450s in their genomes. Interestingly, 23% of *Firmicutes* species had P450s in their genomes [30], indicating most of the species belonging to these two phyla do not have P450s. Analysis of *Bacteroidetes* genera disclosed that of the 130 genera, species belonging to 44 genera have P450s in their genomes (Figure 1 and Table S1). A point to be noted is that only a few species genomes are available in the 130 genera. Sometimes only a single species genome is available, and thus future availability of more species genomes will provide more accurate information on P450s in these genera (Figure 1 and Table S1). However, this study analyzed a significant number of species belonging to genera such as *Bacteroides*, *Capnocytophaga*, and *Prevotella*, and no P450s were found, suggesting that species in these genera probably do not have P450s (Figure 1 and Table S1).

**Figure 2:**
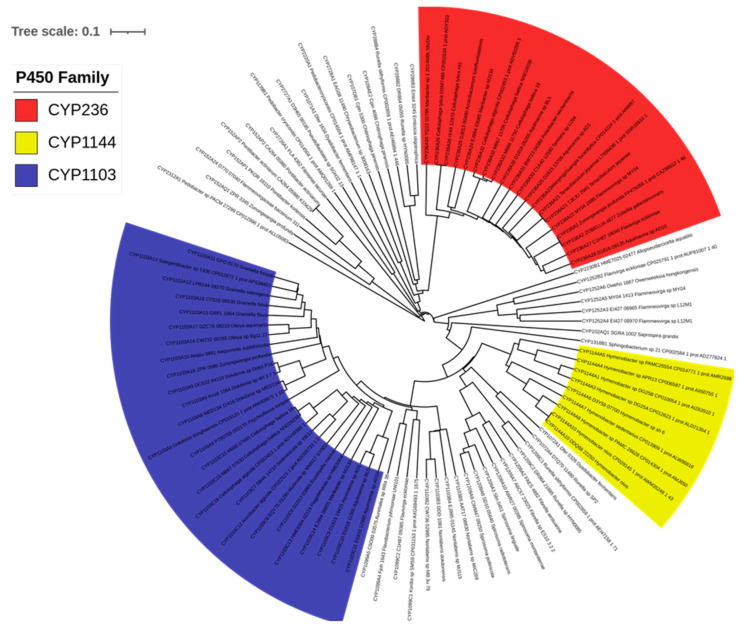
Phylogenetic analysis of *Bacteroidetes* species P450s. The P450 families that are expanded in these species are displayed in different colors.

Analysis of P450s in 77 *Bacteroidetes* species revealed the presence of 98 P450s in their genomes (Figure 2 and Table S1). The P450 count in the *Bacteroidetes* species ranged from a single P450 to three P450s; *Zunongwangia profunda* and *Flavivirga eckloniae* had the highest number of P450s (three P450s) in their genomes (Table S1). *Bacteroidetes* species were found to have the highest average number of P450s in their genomes compared to Gammaproteobacterial species, but the lowest compared to other bacterial species (Table 2). *Bacteroidetes* species P450s identified in this study and their protein sequences and species are presented in Supplementary Dataset S1.

### 2.2. Bacteroidetes Species Have the Highest P450 Diversity

Based on the International P450 Nomenclature Committee rules, i.e., percentage identity of >40% for a family and >55% for a subfamily [53,54,55], and following the phylogenetic analysis of P450s, where P450s belonging to the same family are grouped (Figure 2), 98 P450s of *Bacteroidetes* species can be grouped into 21 P450 families and 28 P450 subfamilies (Table 3). The number of P450 families in *Bacteroidetes* species was found to be higher compared to *Firmicutes* species (Table 2). However, the number of P450 families in *Bacteroidetes* species was lowest compared to Gammaproteobacterial species, mycobacterial species and cyanobacterial species, and *Streptomyces* species (Table 2). The comparative analysis of P450 diversity percentage among different bacterial groups revealed that *Bacteroidetes* species have the highest P450 diversity and *Firmicutes* species have the lowest P450 diversity (Table 2).

**Figure 3:**
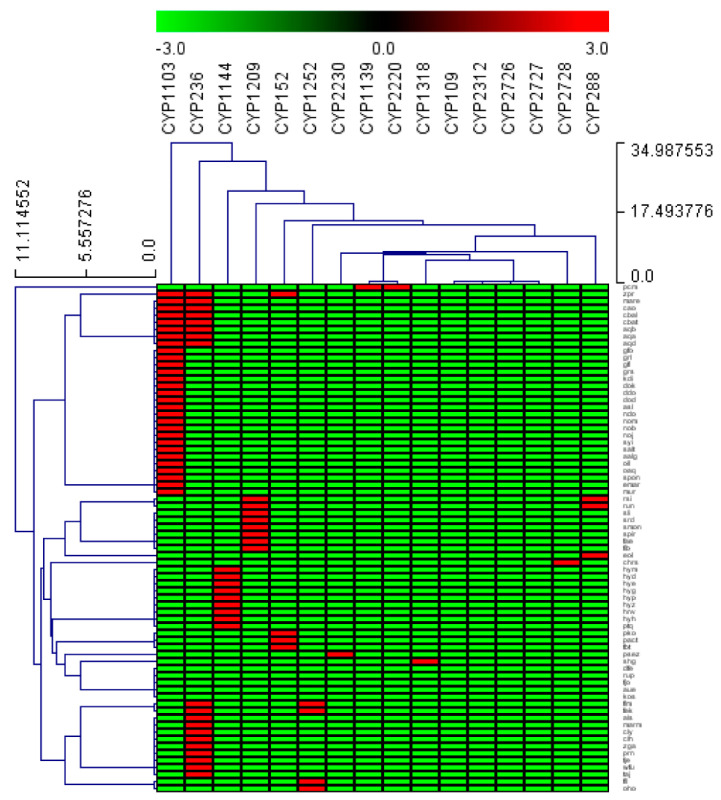
Analysis of P450 family absence/presence and co-occurrence in *Bacteroidetes* species. The data have been represented as −3 for family absence (green) and 3 for family presence (red). There are 77 *Bacteroidetes* species that form the horizontal axis, and 21 P450 families form the vertical axis.

Among P450 families, the CYP1103 has the highest number of members, with 29 P450s contributing 30% of total P450s in *Bacteroidetes* species (Table 3), followed by CYP236 (20 P450s contributing 20%), and CYP1144 (10 P450s contributing 10%) (Table 3). The number of members in the remaining 18 P450 families ranged from one to eight members (Table 3). This indicates that the P450 families CYP1103, CYP236 and CYP1144 were expanded in *Bacteroidetes* species. The P450 family expansion was also observed in other bacterial species belonging to *Firmicutes* [30] and *Gammaproteobacteria* [39]. Comparative analysis of dominant P450 families across different bacterial species revealed that the CYP107 family was dominant in *Firmicutes* and *Streptomyces* species (Table 2). In contrast, different P450 families were prevalent in other bacterial groups (Table 2). Interestingly, P450 families such as CYP102, CYP107, and CYP109 had only one P450 each in *Bacteroidetes* species, although these are quite large families in other species [30,35,50,51,52]. The analysis of the P450 subfamilies revealed that 12 out of 21 P450 families had a single subfamily (Table 3). The P450 families with the most subfamilies were CYP1103 and CYP152, with three subfamilies each (Table 3). They were followed by P450 families such as CYP1099, CYP1209, and CYP1252, each having two subfamilies (Table 3). A particular subfamily was dominant when analyzing the P450 subfamilies in a specific family. In CYP1103 and CYP1252 families, the subfamily ‘A’ was predominant, and in the CYP152 family, the subfamily ‘AP’ was dominant (Table 3). The heat map analysis of P450 family profiles revealed no P450 family conserved across the *Bacteroidetes* species (Figure 3). However, based on the heat-map profile of P450 families, the P450 families CYP1103 and CYP236 were found to have co-presence in eight *Bacteroidetes* species (Figure 3).

### 2.3. Bacteroidetes-, and Firmicutes-Species Have Diverse P450 Families in Their Genome

**Figure 4:**
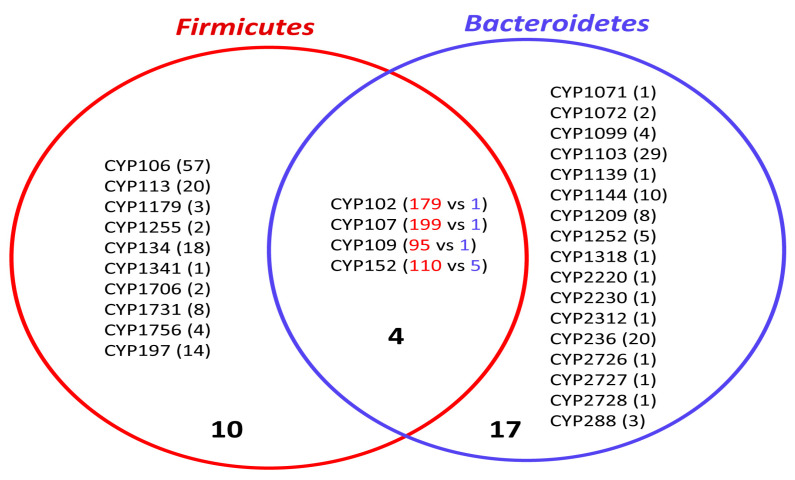
Comparative analysis of P450 families between *Bacteroidetes*- and *Firmicutes*-species. The number in parenthesis indicates the number of members in a P450 family. The numbers indicated with red and blue colors represent the P450 family count for *Firmicutes*- and *Bacteroidetes*-species, respectively. Numbers in bold indicate the number of P450 families.

The P450 family level comparative analysis revealed that only four P450 families are commonly shared between *Bacteroidetes*- and *Firmicutes*-species (Figure 4), indicating these two bacterial groups have a diverse set of P450s in their genomes. In addition to this, the number of members in the commonly shared P450 families was found to be highly expanded in *Firmicutes* species, whereas in *Bacteroidetes* species, one (CYP102, CYP107, CYP109) to five members (CYP152) are present (Figure 4). This suggests that these two bacterial groups have different P450s in their genomes, indicating that P450s play different roles in their physiology, including producing different secondary metabolites.

### 2.4. Bacteroidetes Species Have a Large and Diverse Number of Secondary Metabolite BGCs

**Figure 5:**
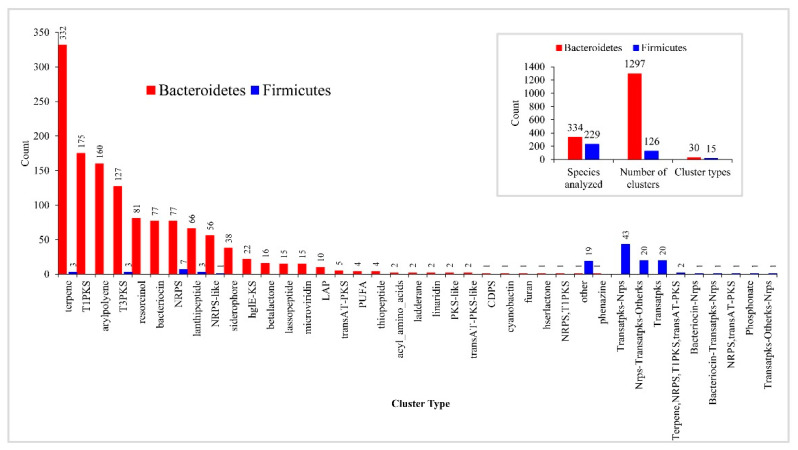
Comparative analysis of secondary metabolite biosynthetic gene clusters (smBGCs) between *Bacteroidetes*- and *Firmicutes*-species. The main panel compares cluster types and the inset panel represents overall features between these two bacterial groups. The number next to the bar indicates the count for that category. The cluster type names and abbreviations used in the figure are the standard abbreviations proposed by anti-SMASH [56].

The analysis of secondary metabolite biosynthetic gene clusters (smBGCs) revealed many smBGCs in *Bacteroidetes* species compared to *Firmicutes* species (Figure 5). In total, 269 *Bacteroidetes* species have 1297 smBGCs, with an average of 4.8 smBGCs in their genome, whereas 229 *Firmicutes* species have 126 smBGCs, with an average of 0.5 smBGCs in their genome, indicating the lowest number of smBGCs in *Firmicutes* species. This suggests that *Bacteroidetes* species produce more secondary metabolites compared to *Firmicutes* species. Analysis of smBGCs revealed the presence of 30 cluster types in *Bacteroidetes* species, compared to only 15 cluster types in *Firmicutes* species (Figure 5). This further indicates that *Bacteroidetes* species produce numerous highly diverse secondary metabolites compared to *Firmicutes* species.

Among 30 types of smBGCs found in *Bacteroidetes* species, terpene was dominant, followed by T1PKS (Type I PKS (Polyketide synthase)), arylpolyene (Aryl polyene cluster), T3PKS (Type III PKS) (Figure 5). Altogether, these four cluster types contribute to 60% of smBGCs in *Bacteroidetes* species (Figure 5). Among 15 types of smBGCs found in *Firmicutes* species, Transatpks-Nrps (Trans-AT PKS- Non-ribosomal peptide synthetase cluster) was dominant, followed by Nrps-Transatpks-Otherks and Transatpks (Figure 5). The smBGCs abbreviations used here are the standard abbreviations that were proposed by anti-SMASH [56].

The comparative analysis of cluster types revealed that *Bacteroidetes* species and *Firmicutes* species only share six cluster types, indicating the distinct nature of smBGCs between these two bacterial groups (Figure 5). However, the number of smBGCs in these six cluster types was found to be very different between these species. In the case of the terpene cluster type, 332 smBGCs were found in *Bacteroidetes* species, whereas only three smBGCs were found in *Firmicutes* species. The difference was also evident for cluster types T3PKS, NRPS, lanthipeptide, and NRPS-like, where many smBGCs were found in *Bacteroidetes* species. In contrast, smBGCs were limited to a single digit in *Firmicutes* species (Figure 5). Most of the smBGCs have no similarity to known smBGCs, indicating *Bacteroidetes* species smBGCs encode novel secondary metabolites.

Considering the above facts, we propose that the contrasting effects of these two bacterial groups on hosts and organisms are partly due to the distinct nature of secondary metabolites produced by these organisms.

### 2.5. Bacteroidetes Species P450s Has a Minor Role in Secondary Metabolism

Analysis of the P450s part of smBGCs revealed that only eight P450s (8%) are part of these clusters (Table 4), indicating P450s play a minor role in secondary metabolism in *Bacteroidetes* species. In contrast to *Bacteroidetes* species’ P450s, 18% of *Firmicutes* species’ P450s were part of smBGCs (Table 2), indicating *Firmicutes* species P450s play a significant role in secondary metabolism. The percentage of the P450s part of smBGCs in *Bacteroidetes* species was found to be the lowest compared to other bacterial groups (Table 2). Out of the 21 P450 families, only 5 formed part of the smBGCs in *Bacteroidetes* species (Table 4). Among these families, four members were from the CYP1209 family. Only a single member from each of the P450 families, CYP109, CYP109, CYP1139, and CYP1318, was part of smBGCs (Table 4). The connection between *Bacteroidetes* species’ P450 families and secondary metabolite cluster type revealed that the P450 family CYP1209 is mainly associated with biosynthetic gene cluster terpene (Table 4). Two P450s, CYP109 and CYP107, from the same species, *Chitinophaga pinensis,* were part of different cluster types (Table 4), indicating their association in producing different secondary metabolites.

### 2.6. Bacteroidetes- and Firmicutes-Species Have Highly Diverse Ferredoxins in Their Genomes

**Figure 6:**
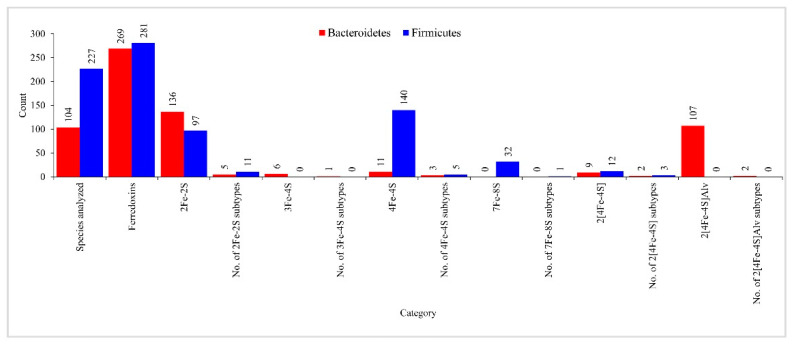
Comparative analysis of ferredoxins iron–sulfur (Fe-S) cluster features between *Bacteroidetes*- and *Firmicutes*-species. The number next to the bar indicates the count for that category.

Genome data mining and annotation of ferredoxins in 104 *Bacteroidetes* species revealed the presence of 269 ferredoxins in their genomes (Figure 6 and Table S2). Among *Bacteroidetes* species, *Tenacibaculum jejuense* has the highest number of six ferredoxins (Table S2). *Bacteroidetes* species were found to have double the number of ferredoxins in their genomes compared to *Firmicutes* species, as the average number of ferredoxins was found to be 2.6 in *Bacteroidetes* species compared to 1.2 in *Firmicutes* species [30]. The 269 ferredoxins found in *Bacteroidetes* species can be grouped into five Fe-S cluster types: 2Fe-2S, 3Fe-4S, 4Fe-4S, 2[4Fe-4S], and 2[4Fe-4S]Alv (Figure 6 and Table S2). 7Fe-8S cluster-type ferredoxins were not found in *Bacteroidetes* species analyzed in this study. Of the five Fe-S cluster types found in *Bacteroidetes* species, the 2Fe-2S was the most abundant, with 136 ferredoxins, followed by 2[4Fe-4S]Alv, with 107 ferredoxins (Figure 6). In comparison to *Bacteroidetes* species, *Firmicutes* species had only four Fe-S cluster types, such as 2Fe-2S, 4Fe-4S, 7Fe-8S, 2[4Fe-4S], in their genomes (Figure 6), indicating the absence of 3Fe-4S and 2[4Fe-4S]Alv Fe-S cluster ferredoxins. Further differences were observed concerning the number of ferredoxins in the common Fe-S cluster types found in these two bacterial groups (Figure 6). *Bacteroidetes* species have more 2Fe-2S cluster ferredoxins, whereas *Firmicutes* species have more 4Fe-4S and 2[4Fe-4S] cluster ferredoxins (Figure 6). Overall, 4Fe-4S cluster-type ferredoxins and 2[4Fe-4S]Alv cluster-type ferredoxins were most abundant in *Firmicutes* species and *Bacteroidetes* species, respectively (Figure 6). This suggests that these two bacterial groups have different preferences for Fe-S cluster type.

Based on the amino acid spacing pattern analysis between the cysteine amino acids of the Fe-S cluster binding motif [31], 136 and 97 2Fe-2S ferredoxins of *Bacteroidetes* and *Firmicutes* species can be grouped into 5 and 11 subtypes (Figure 6 and Table S3). Among *Bacteroidetes* species 2Fe-2S ferredoxin subtypes, subtype 18 has the most ferredoxins, followed by subtype 4 (Figure 6), indicating these species highly prefer subtype 18 ferredoxins. The comparative analysis revealed that three subtypes were shared between the *Bacteroidetes* species and the *Firmicutes* species (Figure 6), suggesting the common ancestral origin of these ferredoxin subtypes [31]. Six 3Fe-4S ferredoxins found in *Bacteroidetes* species can be grouped into a single subtype 8 (Table S3).

Eleven 4Fe-4S ferredoxins found in *Bacteroidetes* species can be grouped into three subtypes (Figure 6 and Table S3). Of the three subtypes, subtype 13 ferredoxins were found in higher numbers (Table S3). Contrary to the 2Fe-2S ferredoxin subtypes, no common 4Fe-4S subtypes were found between these two bacterial groups (Figure 6), indicating that 4Fe-4S ferredoxins are highly diverse in these two bacterial groups. Nine 2[4Fe-4S] ferredoxins found in *Bacteroidetes* species can be grouped into two subtypes (Table S3). Of the two subtypes, subtype 34 ferredoxins were found in higher numbers (Table S3). There were no common 2[4Fe-4S] subtypes between these two bacterial groups (Table S3). The 107 2[4Fe-4S]Alv ferredoxins of *Bacteroidetes* species were grouped into two subtypes (Table S3). Of the two subtypes, subtype 11 has more ferredoxins than subtype 12 (Table S3). Ferredoxin sequences identified in this study and their subtypes were presented in Supplementary Dataset S2.

## 3. Materials and Methods

### 3.1. Species and Database

Genomes for 334 *Bacteroidetes* species, available for public use at Kyoto Encyclopedia of Genes and Genomes (KEGG) [6], were used in the study for data mining of P450s, ferredoxins, and smBGCs. Information on genera, species names, species codes, and their genome IDs is presented in Table S1.

### 3.2. Genome Data Mining and Annotation of P450s

Genome data mining and annotation of P450s were carried out using the standard procedure described previously by our laboratory [30,39]. Briefly, the proteome of each Bacteroidetes species was acquired from KEGG [6] and submitted to the NCBI Batch Web CD-Search Tool [57]. The result was analyzed and proteins that belong to the P450 superfamily were selected and searched for the presence of characteristic P450 motifs, EXXR, and CXG [58,59]. Proteins that were short in amino acid length and lacked one of the motifs were regarded as P450 fragments, and these P450 fragments were not considered for further analysis. The selected P450s were annotated (assigning the P450 family and P450 subfamily) following the International P450 Nomenclature Committee rules [53,54,55]. Proteins with a percentage identity greater than 55% were classified under the same subfamily, whereas those with a percentage identity greater than 40% were classified under the same family. Proteins with a percentage identity lower than 40% were classified under a new family.

### 3.3. Genome Data Mining and Annotation of Ferredoxins

Genome data mining and annotation of ferredoxins in *Bacteroidetes* species with P450s were carried out using the procedure recently published by our laboratory [31]. Briefly, each of the *Bacteroidetes* species genomes was blasted using ferredoxins belonging to different Fe-S cluster types (Table S4), and the hit protein sequences were collected. The hit protein sequences were then subjected to protein BLAST at the National Center for Biotechnology and Information (NCBI) [60] against the Protein Data Bank (PDB) database [61] and analyzed for the presence of characteristic motif of ferredoxins at the Pfam database [62], InterPro database [63], and NCBI Conserved Domains Database (C.D.D.) [64]. Proteins that had a hit against ferredoxins at the PDB database and have ferredoxin motifs, as indicated by different databases, were selected for further annotation. Annotation of ferredoxins (assigning Fe-S cluster subtypes) was carried out based on the characteristic spacing patterns between cysteine amino acids of the Fe-S cluster-binding motif as described previously [31]. Ferredoxins belonging to the new subtypes were assigned a unique subtype number in par with the continuation of ferredoxin subtype numbers published for the species of *Alphaproteobacteria* and *Firmicutes* [31]. Some *Bacteroidetes* species ferredoxins were retrieved from the published article [65] and annotated into different subtypes. These *Bacteroidetes* species names and their ferredoxins are indicated in Table S2.

### 3.4. Phylogenetic Analysis of P450s

Phylogenetic analysis of P450s was carried out following the procedure described recently by our laboratory [30,39]. The phylogenetic tree of P450s was constructed using protein sequences (Supplementary Dataset S1). Firstly, the MAFFT v6.864 [66] was used to align the Trex web server’s protein sequences [67]. The alignments were then used to interpret the best tree by the Trex web server [67]. Lastly, a web-based tool, VisuaLife, was used to create, visualize, and color the tree [68].

### 3.5. Generation of P450 Profile Heat-Maps

The generation of the heat map profile was carried out according to the method previously reported by our laboratory [30,39]. The data were represented as (−3) for P450 family/subtype absence (green) and (3) for P450 family/subtype presence (red). A tab-delimited file was imported into Mev (Multi-experiment viewer) [69]. Hierarchical clustering using a Euclidean distance metric was used to cluster the data. P450 families formed the vertical axis and *Bacteroidetes* species formed the horizontal axis.

### 3.6. smBGCs Analysis and P450s Identification

smBGCs and the P450s part of the smBGCs were carried out following the procedure described by our laboratory [30,38]. Briefly, genome IDs of *Bacteroidetes* species (Table S1) were submitted to anti-SMASH (antibiotics & Secondary Metabolite Analysis Shell) [56] for the identification of secondary metabolite BGCs. Anti-SMASH results were downloaded in gene cluster sequences and Excel spreadsheets representing species-wise cluster information. P450s that formed part of a specific gene cluster were identified by manual data mining of gene cluster sequences. Standard gene cluster abbreviation terminology available in the anti-SMASH database [56] was maintained in this study.

### 3.7. Data Analysis

All calculations were carried out following the procedure reported previously by our laboratory [39]. The average number of P450s was calculated using the formula: Average number of P450s = Number of P450s/Number of species. The P450 diversity percentage was calculated using the formula: P450 diversity percentage = 100 × Total number of P450 families/Total number of P450s × Number of species with P450s. The percentage of P450s that formed part of BGCs was calculated using the formula: Percentage of P450s part of BGCs = 100 × Number of P450s part of BGCs/Total number of P450s present in species.

### 3.8. Comparative Analysis of P450s, Ferredoxins, and smBGCs Data

P450s, ferredoxins, and smBGCs data for *Firmicutes* species were retrieved from published articles [30,31] and used for comparative analysis. Ferredoxins proteins used for data mining were retrieved from published articles [70,71,72,73,74,75,76,77,78,79,80,81,82,83,84].

## 4. Conclusions

Each organism belonging to a particular group is different because it has a characteristic gene pool that is ultimately responsible for its behavior. This study attempts to understand the gene pools of two different bacterial groups, *Bacteroidetes* and *Firmicutes*, that make up more than 90% of the human gut and exert distinct effects on human health. Based on their distinct health effects, one can expect diversity in their gene pools. As expected, these two bacterial groups were found to have a diverse set of cytochrome P450 monooxygenases (CYPs/P450s) and ferredoxins genes in their genome. Annotation and classification of P450s and ferredoxins revealed that *Bacteroidetes* species have more P450 families and ferredoxin subtypes than *Firmicutes* species. A point to note is that the Alvin ferredoxins (2[4Fe-4S]Alv) are expanded in *Bacteroidetes* species, although this is not observed in *Firmicutes* and Alphaproteobacterial species. This indicates gene pool diversity in these two sets of genes in these organisms. Furthermore, very few P450s were found to be part of secondary metabolism in *Bacteroidetes* species compared to *Firmicutes* species. This study strongly supports the hypothesis put forward by our laboratory that organisms’ lifestyles influence the P450 contingent in their genomes. The commensal, pathogenic lifestyle of *Bacteroidetes* resulted in the loss of P450s in their genomes; a few species have P450s in this phylum. The same phenomenon was observed in *Firmicutes* species and Betaproteobacterial species. Analysis of secondary metabolites biosynthetic gene clusters (smBGCs) revealed that *Bacteroidetes* species have many cluster types compared to *Firmicutes* species, indicating that the former produces a more diverse array of secondary metabolites. Furthermore, the smBGCs in these two bacterial groups were distinct, indicating that these species produce different secondary metabolites and, as a result, distinct health effects on humans. A point to note is that, unlike *Firmicutes* species smBGCs [30], *Bacteroidetes* species smBGCs have less or almost no similarity to known smBGCs indicating these clusters encode novel secondary metabolites. Results from this study serve as a reference for further analysis of the gene pools and characterization of secondary metabolites from these two bacterial groups. This study is the first report on a comparative analysis of P450s, ferredoxins, and smBGCs between *Bacteroidetes* and *Firmicutes* species.

## Figures and Tables

**Table 1:** Secondary metabolites produced by *Bacteroidetes* species and their biological functions.

Species (Source)	Secondary Metabolite	Biological Function	References
*Spirosoma* spp. and *Sphingobacterium* sp. 21	Terpene	Neuroprotective, anti-tumorigenic, and anti-inflammatory	[9,14]
*Flavobacterium* spp.	Siderophore	Iron scavenger and transporter	[15]
*Spirosoma* spp.; *Pedobacter cryoconitis*; *Sphingobacterium* sp. 21 and *Chitinophaga pinesis*	T1PKS	Useful as agrochemical and pharmaceutical compounds	[9,16]
*Chinophaga pinesis*; *Flavobacterium johnsoniae* UW10	Arylpolyene	Shields bacteria from reactive oxidation	[17,18]
*Spirosoma* spp.; *Flavobacterium* spp.	T3PKS	Involved in hypocrellin and kanamycin synthesis	[9,19]
*Spirosoma* spp.; *Bacteroides* spp.	Bacteriocin	Anti-bacterial activity	[9,20]
*Prevotella* spp.	Resorcinol	Used to treat skin disorders	[21]
*Spirosoma* spp.	NRPs	Antibiotic, immunosuppressant, and cytotoxic properties	[9]
*Spirosoma* spp.	Lanthipeptide	Antimicrobial, antifungal, and antiviral activities	[9]

**Table 2:** Comparative analysis of key features of P450s and their association with secondary metabolism between *Bacteroidetes* species and different bacterial species. Abbreviations: No.: number, BGCs: biosynthetic gene clusters.

	*Bacteroidetes* Species	*Firmicutes* Species	Gammaproteobacterial Species	*Streptomyces* Species	Mycobacterial Species	Cyanobacterial Species
Total no. of species analyzed	334	972	1 261	203	60	114
No. of species with P450s	77	229	169	203	60	114
No. of P450s	98	712	277	5460	1784	341
No. of families	21	14	84	253	77	36
No. of subfamilies	28	53	105	698	132	79
Dominant P450 family	CYP1103	CYP107	CYP133	CYP107	CYP125	CYP110
Average no. of P450s	1	3	0.2	27	30	3
P450 diversity percentage	0.28	0.01	0.18	0.02	0.07	0.09
No. of P450s part of BGCs	8	126	49	1231	204	27
Percentage of P450s part of BGCs	8	18	18	23	11	8
Reference(s)	This work	[30]	[39]	[50,51]	[50,52]	[35]

**Table 3:** Comparative analysis of P450 families and subfamilies in *Bacteroidetes* species.

P450 Family	Count	Percentage	P450 Subfamily	Count
CYP1103	29	30	A	13
			B	4
			C	12
CYP236	20	20	A	20
CYP1144	10	10	A	10
CYP1209	8	8	A	6
			C	2
CYP1252	5	5	A	4
			B	1
CYP152	5	5	A	1
			A.P.	3
			AQ	1
CYP1099	4	4	A	2
			C	2
CYP288	3	3	B	3
CYP1072	2	2	A	2
CYP102	1	1	AQ	1
CYP1071	1	1	A	1
CYP107	1	1	DB	1
CYP109	1	1	AE	1
CYP1139	1	1	B	1
CYP1318	1	1	B	1
CYP2220	1	1	A	1
CYP2230	1	1	B	1
CYP2312	1	1	A	1
CYP2726	1	1	A	1
CYP2727	1	1	A	1
CYP2728	1	1	A	1

**Table 4:** Comparative analysis of P450s involved in secondary metabolism in *Bacteroidetes* species. The cluster type names/abbreviations used in the table are the standard abbreviations proposed by anti-SMASH [56].

Species Name	P450	Cluster Type
*Chitinophaga pinensis*	CYP109	terpene
	CYP107	NRPS
*Pedobacter cryoconitis*	CYP1139	lanthipeptide
*Sphingobacterium* sp. 21	CYP1318	NRPS-like
*Spirosoma linguale*	CYP1209	terpene
*Spirosoma radiotolerans*	CYP1209	terpene
*Spirosoma montaniterrae*	CYP1209	terpene
*Spirosoma pollinicola*	CYP1209	terpene

## Data Availability

Not applicable.

## Supplementary Materials

The following are available online at https://www.mdpi.com/article/10.3390/ijms23095057/s1.
