# Supplementary material for: Contrasting Health Effects of Bacteroidetes and Firmicutes Lies in Their Genomes: Analysis of P450s, Ferredoxins, and Secondary Metabolite Clusters
Source: Int J Mol Sci. 2022 May 2;23(9):5057. doi: 10.3390/ijms23095057 (PMC9100364; doi:10.3390/ijms23095057)
Supplement: Supplementary file 1 [file ijms-23-05057-s001.zip › Supplementary Dataset S1.pdf]

Article

Contrasting Health Effects of *Bacteroidetes* and *Firmicutes* Lies in Their Genomes: Analysis of P450s, Ferredoxins, and Secondary Metabolite Clusters

Bridget Valeria Zinhle Nkosi [1], Tiara Padayachee [1], Dominik Gront [2], David R. Nelson [3,*] and Khajamohiddin Syed [1,*]

[1] Department of Biochemistry and Microbiology, Faculty of Science and Agriculture, University of Zululand, KwaDlangezwa 3886, South Africa; brilenhle@gmail.com (B.V.Z.N.); teez07padayachee@gmail.com (T.P.)
[2] Biological and Chemical Research Center, Faculty of Chemistry, University of Warsaw, Pasteura 1, 02-093 Warsaw, Poland; dgront@gmail.com
[3] Department of Microbiology, Immunology and Biochemistry, University of Tennessee Health Science Center, Memphis, TN 38163, USA; drnelson1@gmail.com
* Correspondence: drnelson1@gmail.com (D.R.N.); khajamohiddinsyed@gmail.com (K.S.); Tel.: +1901-448-8303 (D.R.N.); +27-035-902-6857 (K.S.)

Supplementary Dataset S1: P450 sequences identified in *Bacteroidetes* species are presented along with their annotated name, followed by protein ID (in parenthesis) and species name.

```
>CYP236A2 Zunongwangia profunda(FP476056.1_prot_CAZ98812.1_4632)

MKKSELPDPFEKARESKGYGEMNDQDDPVTMLLRHKDVRKSAHNYKTFQSGAVPGRIVIPSEVDIRDTRQIPFEVDPPVHGVY
RAIVEPWFKRPLQAEYQEKLTAQISEIVEETLLKGSVEVVTDFALRLQSRALTLLLNTPFSESETWISWGTHVFRSEGEALDG
DKANILYHYIDEQIDRASENPGDDMYSVLLNSEFEGRKLTKEEVKGVMVLTFAGGRDTVINAVTNSIAYLAEHPEALERLRKE
PEITGRAVEEMIRYFSPLTQMGRVVTEDTHVCEHAVKADSRISLCWASANRDAAVFENPNEIVLDRKVNPHVGFGFSHHNCLG
ATHARQILKILLQTLAQKVASFEILDYKENIEDLDHFQRKVGFHNIQIKFNPLTK
>CYP236A21 Tenacibaculum jejuense(LT899436.1_prot_SNR16610.1_2800_)

MKSEFSDPFEEARKKTGLGHIDDQNDPVAMILRHKDVRKTAHNWKTYQSSAVPGRIVVPSEVNIRDIRQIPFEVDPPLHKDFR
DLLEGWFKRPNREEYQKTLKNQVSNLIDDVLSEDEIEVVHDFSLKLQSRALTILLNTEYSESDTFISWGTHVFRSEGDSLDAS
KANVLYDYLDAKINGARENLGEDLYSVLLQAEVNGKKLTHDEIKGIMILTFAGGRDTVINAVTNTVAYFADHPKSLLDIKNNP
EKINKAVEELIRYFSPLTHMGRVVTEDTQVCEHAVKNDTRVSLCWASANRDETVFENPNQVNFDRKINPHVAFGFSHHNCLGA
THARQIMRTLLQLLADKVSSIDIIDSRENIEEWGEFKRKVGFHELKVKFNK

>CYP236A23Wenyingzhuangia fucanilytica (CP014224.1_prot_ANW97227.1_2616_)

MEKSKISDPFEKARVEKGYGNMDDQNDPVTILLRHKDVRKTAHNWKTFQSGGDEVGRIVIPSEVAIRDTRQIPFEVDPPLHKE
FRNLLDGWFKRPNGEEYQAKLAAQIETLVNEVLNKDEVEVVRDFSLKLQSRALTLLLNTPFSESETWISWGTHVFRSEGESLD
ADKANVLYNYIDEKIIEASKNPGEDLYSVLLNSEVNGNKLTHEEVKGIMILTFAGGRDTVINALTNSIAYFAEHPKSLQAIKE
NPEMINNAVEELIRYFAPLTHMGRVVTEDTQVCEHAVKANTRVSLCWASANRDATVFENPNEVDFNRKMNPHVSFGFGTHNCL
GATHARQIMRTLLEILANKVATIDIVDTDENIEELGEFNRKVGYNSITVKFNKY

>CYP236A26 Cellulophaga lytica DSM7489(CP002534.1_prot_ADY30302.1_2467)

MAKCKFADAFKEEREKSGIGKMDDQGDPVVMLLNHKDVRKAAHNYKAFSSEAVPGRIVVPSEVNIRTTRQIPFEVDPPMHKEY
RSLLEAWFKRPLQDTYKEKLTTIIGTVLDDVLSKDKVDVVSDLALVIQSRALTLLLNVPFDKSVKWISWGTHVFRSEGEALDG
DKAAILYNYIDEEIDNAIANPTDNLYSTLLAAEVNGKKLTKEEVKGVLILTFAGGRDTVINALTNMMSYFSANKKALDYIRSK
PEIIKTAVEEFIRYFSPLTHMGRVVTEDTTVCEHAVKADSRISLCWASANRDEKVFENPDEVILERKVNPHVGFGFSHHNCLG
ATHARQIMRITLKLLAEKVSDITVLEAEENIENLGEFTRKVGYNSLKATFKKR
```

>CYP236A32 Cellulophaga algicola (CP002453.1_prot_ADV50295.1_2933)

MKESIFTDPFKKARQETGIGEMDDQNDPVAMILGHKEVRRAAHHWKSYSSGAVPGRIVIPSEVNIRSIRQIPFEVDPPQHGAY
REVVEKWFKRPLEKKYEEELTLQIEALVTKVLDQDSFEVIHDFALVLQSQALTLLFNVSFEEAQTWISWGTHVFRSKDSALDG
AKATVLYDYIDLKLAESSSNPGKDFYAVLLAAEVDGKKLTAEEAKGVLILTFAGGRDTVINAVTNSIAYFAEHPKSLEWLRKE
PELLPTAIEELIRYFSPLTQMGRVAMQDTMVCQHAVKADSRISLCWASANRDETVFENADEVVLDRKANPHVAFGFGTHNCLG
ATHARQVMKVLLRTLIAKVKSIDIHEAKDNIEDLGDFKRKVGFDSLTVKFNKL

>CYP288B4 Runella slithyformis(CP002859.1_prot_AEI46894.1_446)

MDISACPYHSQLSKEFKPFDLTDPFPVYKKAREEEPVFYSEELGYYVVSRYADIQEIFRNWQVFTSENAQTPPKPVPDAVRQI
MVEGGIVGLSGLSGRIPPDHTRIRRIVTMAFTPKRLRKLEPDIRALAIEMIEKFQDKKHAELVKELVYDLPAFVIFMLLGVPK
EEVTQVKAWAISRMMLTFSDTSEEEQLFHARQVVKYWDYCKEMVARRKQQLGDDFPSDLVRLQQEGYEISDREIAAMCYNQLF
AGHETTTSLMGNGIRELLKYRHNWEKLCADASLIPGAIEEILRFNPSVITWRRKATEEATIGGFTFPKGADILLLMGSGNRDE
AVFENGETLDIERKNAKEHLSFGSGIHYCLGAPLAKLEFKIVLEELTQRIPNLQLTPDQTFEFAYNTSFRAPVALEVEW

>CYP1099C1 Kordia sp. SMS9(CP031153.1_prot_AXG69493.1_1675)

MPYHYPNTIPFYKILFKSSSITRNPIPFHKENFEKHGDTFAISPPFSKRIMLTCDAEIILYLLRKNHRNYKKSKIQTKFLSKY
VGKGLLTSSGEYWLKQRRLIQPAFHKEKLQKLVTIMESTIETQLQNLPKNTKVDSYPIMNELAFHVVAKSLFNYSSDEHTMHR
LQEIIETLQDFIIREIRQPHKQWWYKASGLVKKHMTLVKESRDIINTVIDERRNSDKEHDDLLDMLLKAKYEDDGTSMTNEQL
IDEILIFFVAGHETTANALTFTFHLIAKNPEVYAKVLTEIDAIDDAISPMEKIAKLNYVKNCVEESMRLYPPAWITDRVALED
DSFGGFNVQKGTMIGISFYELHRNKKYWENPNDFIPERFSDENRKATTGYYFPFGAGPRMCIGNSFALYEMMLSVYQMMKKYT
IATDPKKVAIAPLITLKPIDLPLTFTKRTV

>CYP1103A8 Dokdonia donghaensis(CP015125.1_prot_ANH59975.1_1031)

MSNDIPAVSVLKFLANAGSILKNPLPFHHDNFESKGDTFKLQLGFGNEVVFSRDPGFAKYALQKNQRNYTKSPIQTKDLAKYV
GEGLLTSEGSLWKKQRKLIQPAFHKKQLAQLIEAMHTVIKEELQNIKTGEAFDVFEIFNDLAFNTVAKSLFQTEVDRKKINRL
QYITEQAQKMLVKELRQPYKSWYFKYAGPIDKHLALTQEARDILMELVEERRASHQKVGDLLDMLLESTYEDGTGMNDEQLID
EILILFTAGHETTSNALTFAASLLARHPEWQDKIYEEYAFAKANSTSLNEFLRHCTVTKQVLEETMRLYPPAYFIDRVNLEED
EYNGMKIPAGSNLLFSCIEIHKHDDFWKDPLQFNPTRFDENAGMYHDAYFPFGAGPRMCIGNNFAMYEMILAVTEMVSRFKIT
PINTPIEILPLITLKPKNAILEFTAR
>CYP1103A13 Salegentibacter sp. T436(CP012872.1_prot_APS38407.1_1110_)

MTEDGKIPEVSLLKFLKHSANILKNPLPFHHNNFQKKGDTFRLNIGFNKSVIFSRDAGLVEYVLQKNQKNFVKSEIQTKDLVK
YVGKGLLTSEGEHWKKQRKLIQPAFHKKQLANLLDSIKQAILLEYKKITVNEELDIFPIYNDLAFQTVVKSLFSSAANQEEIN
RLQYITEETQKMLVKELRQPYLGWWFKASGKIDSYLKLTAEARNILRKIVQERRESNEKYDDLLDMLLEAKYDDGNFMDEEQL
IDEILILFTAGHETTSNSLTFTAQLLALNPEWQEKIYEEISSLKEQDLDLMSYVIKCQITQQVIEESMRLYPPAYFIDRVNLE
EDEFEGKYFQPGSNLLFSIHEVHRHPDLWEDPDAFKPERFAEGGRKYSSQYFPFGAGPRKCIGNNFAMFEMIIAVTELVSQYK
IVPGFDEIDIKPLITLKPKNAILKFQSRS

>CYP1103C12 Arenibacter algicola(CP022515.1_prot_ASO05733.1_2230)

MPFHHENFEAYGDCFRVQLSTKEIVLFTRNPGLIKHILQKQHRKYEKSPLQTVDLAKYIGHGILTSSGEHWRTHRRMVQPAFH
KKKLQNLMGVMREAILMELERIEPDKVQDVFPLMGDLAFQVVAKSLFSSSDIREKMSRLQHITEANQRMLIKEMRQPYLKWWY
RLSGKIDKHLKMAGEAKRLLLDIIEERRSSGLEKDDLLDMLLRARYEDGSPMPDGQLLDEVLILFTAGHETTANALSFTLFLL
AKNPAIQDQVYEEVSKVDFEDSNIDLLQGVMQLQFVKQCIEEALRLYPPAYVIDRVATEDDTFEDILLLKGTMVLMSIYELHR
YSNFWERPLEFDPSRFKETDKKDYGDYYYPFGAGPRMCVGNNFAMYEMIIALAEIVKKYSISSDLKNIEINPLISLKPKAVPL
MFTER

>CYP1103C16 Cellulophaga algicola(CP002453.1_prot_ADV49365.1_2003_)

MKQTPIISQFEVLMNSKRILKNPLPFHRENFEKHGDLFRVPVGIRNELAFTRDPKLIKHILQKQHRKYQKSTLQTKDLAKYIG
HGILTSNGEHWRTHRRMVQPAFHKKKLRNLMGTIREAILLEIDGLSTSNTIDVYPIMSNLAFQVVAKSLFSSDDIQKEMSELQ
YITETNQRMLIKEMRQPYLNWWFQLSGKIKKHLNLAKDGQAILHQLIEERRNSEIEKDDLLDMLLQARYEDGTAMAKEQLIDE
VLILFTAGHETTANALSFLVFLLAKHPDIQEKAYQEVKNISLEGDDVLTQILELKYIQQCVEEAMRLYPPAYIIDRVSIEDDE

FEGLQIPKDSLVLMSIYELHRNEDFWEKPNEFNPERFDASRKKEYQEYYYPFGAGPRMCVGNNFAMYEMVIAVTELLQKYKMT
TPLKAVTVNPLISLKPVDVLINFRNRN

>CYP1139B1 Pedobacter cryoconitis(CP014504.1_prot_AMQ01269.1_4293)

MDTGIILDWNTIEPYTFYTQRRATHPLYYAKAQQSWLIYAYPQAKEVLLHQDALIPELNTSQTGLNKNAVQLIQQLARLNNFE
QHQQSRAAALQIYNQMQPVATQDLLDLLLKPVITGIPVDWVEVVCKRLPALYILKSLNVEAKDCTFLIDHLPVLVKIMSPQQN
PATIKQLNELINLIFPLLEKYLPDGAFAPNYIAAEEWETLLVSNLIGLLIQSYDAGRGLLTNTLLQLSNQQLSLSEAENYFEQ
AVVETLRFDPPIHLTRRVAGKDLLINDQLIKKGEMIIILLAAANLDPQIFESPLTYNPSRGNNKAHLTFGAGHHQCLAKHLTM
RLTAETFKILYHQIQILPQSFTYEPLLNARLVKNLFIIFKNQEK

>CYP1144A1 Hymenobacter sp. DG25B(CP010054.1_prot_AIZ63510.1_1220)

MDTQASDTAAKPFPWVPRWRTLLSSVAMARDPIGNLDRAHAHHGDTVGMHLGGVRPIIVTRDPVLAQHILQKNHRRYLKSDLT
HGLIRYIGRGLLTNEGADWLRQRRLIQPGFHRQRLAGLTRLMQAAAEEWTQELRTRTAAGPALLDIHEAMTRVAFRIISQATF
GTGMSEAERERLSDILTQIQAFYVRTIRQPYLRPWWHLTGSYRYHDALSQELRELVRGYIRRRQAAPNSQTHDDLLQMLLDAR
YEDTGEPMTEDRLLDEANILLLAGHETSANALSWLFYLLATHPQEAVKLREELQAAGLANRPPAFDELSRLPYSLQVIQETMR
LYPPAWIVDRVAQEEDEYQGQRIPKGTLFSVYLYGLHRHPQLWSAPNEFRPERFAPHAQPPVPAYGYLPFGAGPRLCIGNHFA
LTEIQLVVLETLRHFTLELPPNTASVPVPLITLRPPHGMVLRFLSV

>CYP1144A3 Hymenobacter sp. DG25A(CP012623.1_prot_ALD21354.1_1672)

MDTRTTDTAAEPFPWVPRWRTLLSSVAMARDPIGNLDRAHAGRGDTVGMHLGGIRPIIMTRDPALAQHILQKNHRCYLKSDLT
HGLIRYIGRGLLTNEGADWLRQRRLIQPGFHRQRLAGLTRLMQAAAEEWTQELRQRTAAGPALVDIHEAMTRVAFRIIAQATF
GTSMGDTERERLSTILTQIQAFYVRTIRQPYLRPWWHLTGSYRYHDALSQELRELVRGYIRRRRAASNSQPHDDLLQMLLDAR
YEDTGEPMTEDRLLDEANILLLAGHETSANALSWLFYLLAAHPEAAAKVRAELQSAGLANRPPVFDELPRLPYSLQVIQETMR
LYPPAWILDRVALEEDEFQGQRIPKGTLFSIYLYGMHRHPQLWAEPDEFRPGRFAPHAQPPVPAYGYLPFGAGPRMCIGNHFA
LTEIQLMLVETLRHFSIELPPDTASVPVPLITLRPPHGMLLRFQQIQ

>CYP1144A4 Hymenobacter sp. APR13 (CP006587.1_prot_AII50755.1_395)

MSEFPELPPAALAGPLPRVPRWRTLLGSLAMARQPIRNLDRALAHHGDTVGLHLGGVRPCIVTRDPALTQHILQKNHRRYLKS
DLTHGLIRYLGRGLLTNEGADWLRQRRLIQPGFHRQRLAALTRLMQAAAEEWSQELRARLAAAGGRLTVDIHAEMTRVAFHII
AQATFGTSMTDAERDRLSEVLTQIQAFYVRTIRQPYLRPWFTARGAFRRHDALSQELRELVRGYIRWRQAAPGTLASPEIAAP
FTNNPPPVTPNLSPVTPTPPNDLLQMLLDARYEDTGEGMSEEQLLDEANILLLAGHETSANALSWMLYLLARHPEAAAQVRQE
RTAAGLARRPPEFAELAQLPYSMQVVQETMRLYPPAWILDRVALEDDEFRGLPIPKGTLFSLYIHGIHRHPGLWPEPNAFRPE
RFAPGQEPPIPAYAYLPFGGGPRLCVGSHFALTEIQLVLLEALRHFTFVPVAEAPAATDPLITLRPKGPLWLAVGSA

>CYP1144A5 Hymenobacter sp. PAMC26554(CP014771.1_prot_AMR26880.1_1392_)

MVDTPLPTPAWPRIPRLITLRSSLQAARDPVGNLNKLLAHYGDTVHLFLGGARPSVVTRDPALVQHILQKNHRRYAKSDLTHG
LVRYLGRGLLTNEGADWLRQRRLIQPGFHRQRLAGLTRLMRRAAAEWVQDLATQLTDQQAVAELDAHAAMTRVAFRIVARSVF
GDSLGEEQLTRLSDWLTAIQAFYVGTIRQPYLRPWHWVRGSYGRHDQLARNLRELVRGAILRHEAALAAAPADALPPDDLLQM
LLDARYEDDGTAMSENQLLDELNILLIAGHETSANALAWAFALLAHHPEKQDKLAAEIERELPGGQPVEFEDLPRLPYALHVV
QEAMRLYPPAWIIDRTATEADEFEGVPIAKGTIFSLYIYGLHHHPGLWPEAEVFQPERFAPGAEPVPPPYGYLPFGGGPRLCV
GQQFALTELQLVLIQALREFRVEPANAPIPGMNPLITLRPAGELRLRFRRRVTG

>CYP1144A7 Hymenobacter sedentarius(CP013909.1_prot_ALW86816.1_3553)

MGEVETLEKAVAPFSLPRVPRWRSLLRSFALAKDPLPVLDATLARYGDTVELFIGGVEKSILTRDAGLTQHVLQKNSRNYAKS
KFTQGFSRYVGHGLLTNEGPDWLRQRRLIQPGFHRQRVAGLTGLMQEITAETLAPLAAQAAGGGGAVAVPVHELMTRLTFRII
ARSVFSTNFPEAELDRLAQLITEIQAFFVRSIRQPYLKPWFRLRGRFRYHDALSAELRALLGRYIAQRQQANAQPNATPPDDL
LQMLLDVRYEDTGEPMTPDRVLDEALILLIAGHETSANALTWLIYLLAHHPEEAASIRAETEAVLAGRSPTFDDLPRLGRALY
AVQEAMRLYPPAWMVDRVALADDHYQGLHIPKGTLFSLYFYGLHHDAKYWANPHEFRPARFAPGQARALQPFAYVPFGGGPRL
CIGMQFALTEMQLVTLALLRQFEVEWVEGQPAVTMQPLITLRPKNDFRVRLRLR

>CYP1144A8 Hymenobacter sp. PAMC 26628(CP014304.1_prot_AMJ65095.1_1186)

MADDTLGPRPPHAPLPQVPRWHSIRGSLRLVADPIGALDAVHADYGSTARLYIGGVQPSVLTRDPGLVQHILQKNHRNYAKSK
FTLGFARYIGHGLLTNEGPDWLRQRRLIQPGFHRQRVAGLTGLMQEVVADTLGPLAQAAARAGGAVAVPAHALLTRLTFRIIA

RSVFSTRFAEAELDRLAQLITEIQAFYVRIIRQPYLNWWRRLRGQFRHHDRLTAELRALLGRYIAQRQAASAAPGAPAPPDDL
LQMLLDARYEDTGEPMAPARVLDEALILLVAGHETSANALTWLLYLLAHHPAEAQAIQAETAAVLGTRAPTFADLPRLGRALQ
AVQETMRLYPPAWMVDRVALADDDYQGLRIPKGTLFSLYLYGLHRDPQHWDAPAEFRPARFAPDAPRPLVPFAYAPFGGGPRL
CVGLQFALTEMQFVAVELLRTFDVEWPAGQPLVVKQPLITLRPRGDFQVRLRLREGPRAAAG

>CYP1144A10 Hymenobacter nivis (CP029145.1_prot_AWM35248.1_4395)
MADDDLGGRVPRWALPQVPRWVSLRNSLKLVADPLGALDAIHARFGATARLYIGGVQPSVLTRDPGLVQHVLQKNHRNYAKSR
FNLGFARYIGHGLLTNEGPDWLRQRRLIQPGFHRQRVAGLTGLMQEIVADTLGPLAQDATRLGGAVAVPAHALMTRLTFRIIA
RSVFSTSFADAELDRLAQLITELQGFYMSTIRQPYLNGWRWLRGQFRYHDARTAELRALLGRYIAQRQAASAAPGAPAPPDDL
LQMLLDARYEDTGQPMAPDRVLDEALILLVAGHETSANALTWLLYLLAHHPAEAQAIQHETAEVLGNRAPTFADLPRLGRALH
AVQETMRLYPPAWMVARVALADDDYQGLRIPKGTLFSLNLYGLHHDPQHWDAPAEFRPARFAADAPRPLVPFAYAPFGGGPRL
CIGLQFALTEMQLVVVELLRVFDVEWPVGQPPVAKQPLITLRPRGDFQVRLRLR
>CYP1209C1 Runella slithyformis(CP002859.1_prot_AEI47158.1_716)

MKYSKSGVSTIPQAKKNPFFGNTPDFVRNPLRFLEKMQKEFGHVGVVKLSLVNRDFFLVLTPEDTKHVLQENNRNYHKSEAYK
VLAIFLGNGLLTSEGDFWRRQRKLTQPAFYKQRLALMVEMMNREVATAVEGWERKNGEEAFDTTEEMLNLTLKIVTRALFSTD
VKHRLGGISESLNEIMHFADSTLKSFIRLPLTVPTPRNLRFKRAVAKVEAVIYSIIEGRREEIKQNAHVRYNDLLDMLIHTRD
EETGETMTDQQVRDEVTTIFMAGHETTANALSWALYLLSKHRDVLHKLREEVKMVLGEEGMPTFETIRELKYTLQVVQEVMRL
YPPAWVMGRKALGDDQLSGYSIAAGTYLLLPIYLLHRDPKYWQKPNEFYPDHFLPENIKARPTYSYIPFGGGPRMCVGNNFAL
MEMQIVLALWVRRLDFTLIDQKAMEADPLVTLRPKKSLKMYVKAFRQT
>CYP1252B2 Flavivirga eckloniae(CP025791.1_prot_AUP81007.1_4099)

MTDNQLVEVRELKSPKGAFLLGHLPQFNTYNKHQVLERWVEECGDLFKIHFVGKEFVVSANPDINNKMLRLRPEGFKRFSKID
EILKEMGVDGVFNAEGDTWKRHRKPIAEALSVKNVKAYYPIILDKTNRILEKFKNYSQQKTIVDVQKEFMAFTIDITTEIAFG
HKLDTINNRADSFQKHLEVIFPMINTRVTAPIPIWRYFKRKKDKTLDDSLKAIEKVIYEFIDGAKKRIAENPKLKEHPSNFLE
ALLVENGDVNFTDKEIYGNVFTMLLAGEDTTSNSLSWAMFYLAQHPETVEKVREEAKRVYTEDAPNNYENVEDLKYANAVAQE
TMRLKPTTPQLYLESNDDIIVEHVVIPKGTSIILQNKVAQTQDDYFSSPDDFVPERWLSGGCPMHENHAPNVMRAFGGGARYC
PGMHLAKTEMVVLISTLCKHFNFELEVAPEDVREKFEFTMYPGNLKVTFTPVVD

>CYP1318B1 Sphingobacterium sp._21(CP002584.1_prot_ADZ77824.1_1231)

MDLLTDKNIPFGPKEASEVEEGPQALSNILNLFHQYGDIYKIYSERRNNYTYVISDPEMVKHVLITNNRNYEKGVGIDRVKIL
LGNGIMVSEGNYWKRQRRMIQPAFHKRVIAKLTDDIAQANETMLSNWLTGNKEINLTAELSSVTLRIVLQALFSVDFQQLEKR
EGVNPFALLTEVHERNLVFAMKFRALAKTIQEIINLRRKEHRVEEDFLSMIMEAKNDEGQGMSDREIIDEMMTLIVAGHETTA
SALTWAWYLLHKHPEVYARAKQEALQVQNVHLGFHHLEQLPYIRQVIEETMRLYPPGWLLTRRAMQDDVIGGYHVPPKTDIFI
SPYVIHRHPRYWEQPDLFNPERFDASYRRERHRFEYFPFSGGPRQCIGDFFALVEMQLHLALILRTTDMEILVDEPISMEAQI
NLRPDKPLFARLINPRN

>CYP2220A1 Pedobactercryoconitis(CP014504.1_prot_AMP98147.1_1172)

MTTNADEPRYSVADPYPWYKWVQQKKPIYRTPEGMWMITGYEDAMMLLSDPRCSHWGQDSKTFQYLSPVEKAIAQTLHALAPG
NTPAFRKQIMHQLAARTLQIDEDDMKRQADEILDGLRSSSGMEFMNDYAHPFTFGTICSVMGVPQEEVGAFSKIVGRLQGGYL
SFIDEKSWSNGEDEQKKIFIDTLRRLIGLKRQTPGKDLCSAILAVVPADEQDDSYLISLMVLLFYAGHQNMMNFMGNALVALQ
DRVEDQSMLRESLPFAITSVDELIRYDSPLQSVLLITQESINLHGTIIPAGSQLLVSIGAANRDAAKFDDPDQLILARRPNHL
GFGAGAFRCIGARLAQIQGGIGLHRFFAHVNSYAPVADPISWSHFSVQRGPSSILLDINWNHER

>CYP2312A1 Pedobacter sp. PACM 27299(CP012996.1_prot_ALL05083.1_1150)

MSTTPVKVHKLPRLALLLANHDRALFYRKLNETGDAFLIEEDRHWLINSPNLITQLFHEPSLDSNRQNLKKLLIGQGTPDSIN
FFYQNWLMYMSGAEHQFWREKFIACFPKRMELSYKYFDMANAIGTSIKEFDLIKQVINPYVVHMLCEIAGMKVTAFQNAYHLI
NPILQLLHGRGQVDNPTTKHNALDEWSTIIERLTREQKLTKQGFLSQLAKTGKLGLAVGLLPFLDVVDALIALCGRVVIDYAA
LKQQQNIKIQQEKLLDLIKLYSPFQVCNRKVINPITHFPELDFQYGDKVSLMIGAANSNTPKRDVAEIQSIPNLKNFSFGIGQ
HSCPGRNWSITIVMEFYKSLDNYLTEYKASIKVKNINHREEFGFQGIQNMIVLLKLP

>CYP152AP2 Pontibacter actiniarum(CA264_05980_K15629_)

MKNIPHDNTLDSSISLLLEGYPFLKKRMEDHQTDIFQTRLLGEKVICIHGKEAAALFYDNAYFWREGVLPKRVQQTLMGKNGV
QMLDGEAHRHRKALFMSFMSRDRIDDLMGLMLRYWRAYARKWEKMERVVLFDEAREVLCLAACEWAGVPLKPTEVREHAQEYI
EMIYGFGGATTRYWRGIHARNVSEKWNKKIIEDIRSRKLEVPENSAAYQIAWFRDLDGELLDLHVATVELMNVVRPIVAIATY
VAFSALALHEHPEQVEKIRTGEEKYAQLFVQEVRRFYPFTPFLGARPHRDFEWKGHQFKEGTLVLLDVFGMLHDPEMWPDPYA

FKPERFTEWSGSPFDFIPQGGGDHYTGHRCAGEWITIKAMKVALQFLTQEVKYIVPEQDLEVDIHKMPTLPKSGFEISDVQYT
GSNVYAEV

>CYP109AE2(Cpin_4098)Chitinophaga pinensis

MTPSTLKSPLSWHQQLQDEQAVYFDPAFRFYFGGQGAWQVFRHKEVQRVLSDHEVFSNEY
MPKSDDNLLGSNLNQTDPPRHRQLRALVSKAFAPAVIAKLEAWIHHECKELLQTVLAKGE
MDFVKVFSIPLPGRVTAQLLGVPDQDHDQVNAWVNAISSDPAVIGMDAYFQAQQEMGRLF
TALLEERAKTPQSDLISHLLHAEIDGERLSMPDTLAFCIALLIAGNETTNGFLANAMYTF
ATTPDVQSHLQAHIEDLPSALNEVLRYAPPVQSMCRIAKMDVELGGQLIRKGDLINAWLS
AANRDPSVFRNPDTFDIHRNNIKMVSFGHGAHYCIGAMLARMEAKIAFEIIFSAIKNVTL
KPGVTPARNPSTIVAGFLDLPIVFEPK

>CYP107DB1(Cpin_5300)Chitinophaga pinensis

METTTQTAKCPFAGKAIPDIDFSDSAFIKNPFATYAAMRDEAPVHRVAFSSGQPFWLITR
YEDAMLVFKDPRFTKDIKKTLPPDHKAPASPLAMSQLFSHHVLYMDPPDHTRMKQLVQKA
FTPKLVEGMRAHIQDITNNLLDKHIPSGRIDIINDYALPLPISIISGLLGIPEKDQQLFR
RWSNIILNIDINVTREERMQMMPEAIGGFTNYLREIFASKQVHPADDLITHLVQAKEGSD
KLNETELMSTVFLLFAAGYETSVNLIGNGVLALLQYPEQQLALRNDPALINTAIDEVLRL
DPPVSLASERYTLEDVEMNGVTIPKGELVHICITAANRDPRRFESPDAFDITRKDNKHLS
FGQGTHYCVGSALGKLEGEIAINTLLKRIPAFSVEGGDIDALKYKNNSVMRGLEALPIVF

>CYP2726A1(FLA_4362)Filimonas lacunae

MSISNSLKVWVSSAPVLRPVFSILRKYKPVARIGKTVVVTRYKDVMDVLKRETDFTVYEI
DGYKMERMGNPFVLGMDASPETTRDRDILRQVIKREDLKTIRVMIRHIANDLLEQAQPNH
TIDTVNGYARLASVRVVAQYFGVPADEATMMRWQRSIFSEAFANLNDNKAIRERGMIAAK
EIAAHLNQLIQQRQQQTTPLEDNVLNRLIQLQPYNSWLNNDAVRRNILCILGVVENTSKV
VTHIIDQLLKRPDIMKACQQAVHANDMETLRSYCFDILRFNPHNPIILRYCKHGAVIGKD
TPYERRVPAGSTIYAATLSAMFDEDIVHNAKQIDPNRNVEYMHFGYGPHVCSGKYISEVT
VPELVAGLLRLKNLQRAPGKAGKIQYEEVVFPKSLSLTFN

>CYP2727A1(D3H65_00195)Pseudoflavitalea sp. 5GH32-13

MQHHTKQKTFMQPVLFPQSTVNNPFEIYARKLQESPVYRDEAQQVWGIYSYEHCLQLLTG
NDAHIPALPVLPAGTLNDQVLTIIEHHTRLNNGTAHRSTREIAMGLYNARLPISPIGLLA
TSLSHKNLRNEIDWVQEVGKQLPLACMLQEFQFNEKDRESILMYIAVLVKIMVPDKTAQQ
IAAINAATREVYQLTERHILHTPSLYAIAHNVSKVSFPTALAMTVANLVGLMIQSYDAGR
GILCNTLLQALQHRELAGQPGKEKAMGQLVMETLRYDPPVHHTRRVLTNDVLLHGQELKK
GDTAILVLAAANRDAMHFERPDAFDIYRANNEAHLTFGAGAHRCMANHSTVRFATEILNY
LLTRYPRLQLLTTEITYEPAMNVRLPKEMMLSLS

>CYP102AQ1(SGRA_1002)Saprospira grandis

MKSPVSKPIPHPPIYPIVKSVLSLDIKRPVQSMMALAEKYGGIYRLEVPNDSLVIVSGLE
YVQEFCDEDRFDKKVFSALEKVRDLTGDGLFTAHTEEPNWGKAHRILTPAFGPHAMQDMF
DKMYDVAEQLCVKLERLGPDEPFNVPANMTRLTLDTIALCAFDYRFNSFYKNEMHPFVEA
MLFILHEANQHMRRLPIMNRLMYKTKERYNKDIQYMYTVAQKILDQRRKADKAEQVDDLL
GRMLEGVDPDTGEKLSDQNIIYQMVTFLIAGHETTSSMMAFTFYEMLKQPHILARVQAEV
DEVLGQEKLQFHHLSKLKYMDMVLKECLRLWPIASGFNLRSFKDEQVGEYLIKPTDSIFI
FLPSLHRAPIWGEDPKLFNPENFSPENEAKIPPSAYLPFGNGRRSCIGRPFAFQEAKLAI
AMILQRFDISLADPNYELVLDETLTIKPDNYFLKFKKRPGFKPVENGPKVPASQSLKLKG
GKQIYVQNKPLLYLAYGSNMGSSKKFIQQLAETAQALGYQPSVHSLNETKALMAQNKGHY

VIITASYEGQPTHDAEEFVDWIVEQPEGSLDQVQYSLFGCGNSDWIHSYQAVPRKIDAQL
KRLGAQPFLAPGEGNAKQNFFASFNQWHDELWPALAKSLGQDAISPINKKSYKVQLLHDW
HAQTLGENDFVRAQLLSKKELVQQESPFASSKIELQIEVPEGQSYQTGGYIDILPQNSPA
TIQRVLRHFQMQHDPYIKLESEEQSVALKLPLDQHIRLTDLLKYYVELQRPAGKKWLEEV
LAQTRCPFSAQGLQQFIEQYESEVLQKNRSLLSIIEQFPGAKIDLGQFISALPAMRPRTY
SISSSVKQSTDKASLVVSCIKGAAWSGQGEYQGLASGYLSQLEEGQLLWLRFSPNLPNFP
APKSEDKLILIAAGTGIAPFRAFMQERSLEKTPEKAVFFYGCRAKEVDQLYAEEFAQWEK
EGWLDLRPVYSKAPEKEGQTYVQHRLWADRDAFWAAWEAGAKIYICGDGEGMAPAVRQCL
MDIYQEKAQATEAQALDWLMQIAGKDKRYFTDIFS

>CYP1072A1(Dfer_0326)Dyadobacter fermentans

MQAIPEFKISSVPLVSPIRFARDPLRFLRQGFDECGDTFKIKLFREFIVTRDPAFFRHVL
QQHHKNFKKGNSVKMLRPVLGNGLVISDGDFWLRQRRLVQPAFHRERLQELFVTMGGLTA
AFLDEMEQFRGKAPVDVDAKMMGITSDIALKTLFGNMNTEDKEQIYNQVSRTQTYLVTRV
RKPYRLPLMAINGEDRRFKSDLAYFNSLVYDFIRKRRLSGETPNDLLQLLLDSTDEETGE
QMTDEQIRDEAITMFAAGHETSATGLSWLLWELSAQPEIVARIRQESSIFETVPSFEQLI
QMPYTRQVVEEGLRLYPPAWTMTRESTVDQKIEDYPVPRGSSVFMSIFELHRNPNLWHNP
AAFDPERFQPEAVKNRAKFNYLPFGAGPRICIGQQFALMEMQLVLAALVKRFDFVREPGY
SVGMHPQIVLKSTNGIKLNIR

>CYP1071A1(Dfer_1634)Dyadobacter fermentans

MQTPLMSATSIWSAKKEVNQNYTELIADVRKSDPLHINMFGDLVAMDYANVKKILADSDN
FKNFDFTERFKVVSALSDNDPGLLEFGESLRYWLLFMNGEKHAEHRRFVNQKFYQANYEQ
ITLDAITEVIAALDGQEEADLVEMARQFSFLIISKIIDLDKADFDFIQKFSYVITFIFEK
TLSVTELLECASMSRTFHSYLSETFSRHEREATNSLLLEMREVMGSARAPQLIGTWEFLV
NAATETTTLLLTKSIAALIENRDKVINWNAHNECAIAVEELIRYVSPVNWIPRQVAAEME
FEGLQLRKGQTVLLGLASANRDPAVFQNPDTFTPTRKPNPHIGFGFGMHHCLGARLSRFE
LQKFLPRFMAAFPDIRLHPTKAPQWDSKIFFRGFKTLPVLLK

>CYP1209A3(Slin_0451)Spirosoma linguale

METTAAPTRPVPLHPGLPFLGNTIAFVRDPLSILHTLQRKQERIVHLRIGGRHQYLVFQP
EDSKHILQENNRNYGRSPAFEVLKIFLGNGLLTSDGDFWRRQRRLAQPAFHRQKLAALAD
AMVAETADWLDTLNPSDIRQPINVSQAFMDVTMRIVCKTLFGSDTNGKLDGLSHALDSLN
YLANSRMLSPIRFPMSWPTPHNQRSKRAQRQVDEFIYGLIDQRRQQHEDKDDLLGMLLSA
EDEETGERMSDQQLRDECVTLFSAGHETTAVSMAWTTYLLTQNPDILARLQVESETILGD
ARTPPADAFRRLTYTMQVVQESLRLYPPAWIMSRRAREDDHIGPYTIPAGDTALVCPYLL
HRDPVNWPDPERFDPDRFAPGGPKDQLHSYAYLPFGGGPRLCIGNQFALMEMQILLALFV
RKFSVSGPPNQRIVPKPLITLRPNQPIKAILSKP

>CYP1209A8(SD10_05640)Spirosoma radiotolerans

METSTAPTQPVPLHPGLPFLGNTLAFLRNPLGTLDTLQRNHDRLVHLRIGGRHQYLVLQP
EDAKHILQENHRNYGRSPAFDILKIFLGNGLLTSDGDFWRRQRRLAQPAFHRQKLAALTQ
TMISETADWIDELSGHNLKEPVNVSQAFMDVTMRIVCKTLFGSDTNGKLDGLSNALDTLN
YLANNRMLSPLRFPYSWPTPNNQRSKRARMQVDSFIFGLIDERRKALDERDDLLGMLLSA
EDEETGERMSNQQLRDESVTIFSAGHETTAVSMAWTIHLLTKHPDVLARLKAESQSVLGD
ARTPPPEAFRALTYTMQVVQESLRLYPPAWIMSRRAHQNDHVGPYTIPAGDTALVCPYLL
HRDPANWPNPDRFDPERFAPGGLKDRLHSYAYLPFGGGPRLCIGNQFALMEMQILLALFV
RRFTPKAAGNQRIVPKPLITLRPNQAIRAVLE

>CYP1209A10(AWR27_00395)Spirosoma montaniterrae

METLTSSTRPVPKHPGLPMLGNTLPFMRDPLAILQTLQQRYDRLVHLNIGGRHQYLVMTP
EDAKHVLQENHRNYGRSPAFRVLQIFLGNGLLTSDGDFWRRQRRLAQPAFHRQRLAALAQ
TMISETTDWITELSRLDKSKPVNISQAFMDVTMRIVCKTLFSSNVVGKLDGLSEALETLM
HLSNNRMLSPFRFPMSWPTPPQRRFRRAAQVVDTFIYGVIDQRRRSNERYDDLLDMLLYA

EDEDTGDWPNSGRMSDKQLRDECVTIFAAGHETTAVSMAWTMHLLTQHPDVQARLRDEVN
ERLGDAATPAPETFRSLTYAMQVIQESMRLYPPAWIMSRLAHADDQIGPYTIPAGDTALV
SPYLLHHDPANWPDPERFDPERFAPGWEKERHSYAYLPFGGGPRLCIGNQFALMEMQILL
TMLVRAFAFQPIPNQRVKPQPLITLRPKRPVWLLMSNQIS

>CYP1209A9(CWM47_09250)Spirosoma pollinicola

METTVISTQPVPLHPGLPFLGNTLAFLRNPLGTLHTLQQNHARMVHLRIGGRNQYLVLKP
EDAKHVLQENNRNYGRSPAFEVLKIFLGNGLLTSDGDFWRRQRRLAQPAFHRQKLAALTQ
TMIAESADWIDELKGHNPKEPVNVSQAFMDVTMRIVCKTLFGSDTTGKLDGLSTALDTLN
YLANKRMLSPLKFPYAWPTPNNLRSKRARMQVDTFIYGLIEQRRKASEERDDLLGMLLSA
EDEETGEGMSDQQLRDECVTIFSAGHETTAVSMAWTIHLLTNHPDVLARLKAESHSVLGD
ARTPAPEVFRGLTYTLQVVQESLRLYPPAWIMSRKAFNDDHIGFYTIPAGDTALVCPYLL
HRDPANWPDPGRFDPDRFAPGGPKDSLHPYAYLPFGGGPRLCIGNQFALMEMQILLALFV
RQFDLKAVPHQRIFPKPLITLRPNQSIWVTLN

>CYP288B2(DR864_05055)Runella sp. HYN0085

MSTCPYHKISEEFKPFDLTNPFPFYKKSRDEQPVFYSQELGYYVVTRYEDIKAVFSNWKT
YTSENAQSPFKPIAPKAKKLMEDQGMIGLSGLSGRIPPDHTRIRRIVSMAFNLSRFKKLE
PGIRALAIEMIEKFENEGHAEIVKQLAYDLPALVIFMLLGVPKEDVQQVKSWAESRLLIT
WGDLTEEEQLVHAQNMVRYWEYCQNLVALRKENPTDDLPGDLVRLQAEGHEISDREIAAI
CYSQLFAGHETTTSLMGNGIRELLLHPDSWKAICENPSLIPNAIEEILRFSPSIVSWRRK
ALDDSEIGGVTIPAGSNLLLVMGSANRDEANFENGETFDIQRPNAKEHLSFGYGIHFCLG
SPLAKLEFKIVLEELTRKIPHLTIKENQVFQFALNTSFRAPVALEVEW

>CYP1209C2(DR864_21885)Runella sp. HYN0085

MLFLLDIKEHFLGKVQKNVTKIGKENDISEGYSNILCTTIMRTIMMNYSKSALSTIPEAK
KSPFFGNTSAFVRNPLGFLEKMQKEFRGAGLVRLNLVNRDFILVLNPEDTKHVLQENNRN
YHKSEAYKVLAIFLGNGLLTSEGDFWRRQRKLTQPAFYKQRLALMVAMMNDEVTNLVQRW
EQHEKTETLNMSEEMLNLTLKIVTQALFSTDVKHRLGGISESLNEIMHFADNTLKSFIRL
PLYVPIPRNLRFKRAVKKVESVIYSIIEGRRQELKHNPEVRYNDLLDMLIHTQDEETGET
MTDQQLRDEVTTIFMAGHETTANALSWAFYLLTKNGDVLHKLREEVKTVLGEEGMPTFEN
VRELKYTFQVVQEVMRLYPPAWVMGRRALGADQLSGHPIGPNTYLLLPIYLLHRDPLHWQ
KPHEFYPDHFLPENVKQRPTYAYIPFGGGPRMCVGNNFALMEMQIVLALLVRKFDFLLVE
RKDAVADPLVTLRPKKALKMNIKALSRLEVN

>CYP1072A4(DTQ70_11480)Runella sp. SP2

MPVLPTYKPSKIPLTIPLRFAKDPLSFIRSGFSACGDTFRLQLFRDIIFSRDPAFFRHVL
QQNHRGYAKGKAFTELSKVLGKGLLTSEGDFWLRQRRLIQPVFHRERMLGLYQIMAELTA
QFVKDFEANRNQSAIDLDEKMMAITADIALRTLFTTITNEDKATIYQQINRTQEFIIANI
RKPFLKPWMAINGANRRFRADLSYFNRLIFDIIEQRRASSQPQDDLLQMLLDCTDEETGQ
QMNDQQVRDEAITMFAAGHETSANGLNWLLLELSKHPEVVQKIREECSIFDTVPTWEQLL
QLTYTRQVVEEGLRLFPPAWAVAREAIADDVIEGFEIRKGTIVFLPMYELHRNPEFWHQP
LSFDPSHFAPENVKNRPKFAYLPFGAGPRICIGQQFALIEMQLILASLLKRFTFIPDDTH
NPKMFPLITLKPMNGIKVWVK

>CYP288B3(Emtol_3245)Emticicia_oligotrophica

MNTAIGCPYHKVSESFKPFDLTNPFPFYKQAREEEPIFFSEELGYYVVTRFQDIKEIFGN
WKVFTSENAQSPFKPIAPKAKALMEEGGLIGLSGLSGRIPPDHTRIRRIVSMAFNVGRFR
KLEPKIRELAINMIEDFAAKGKTNIIKDLAYDLPAYVIFMLLGVPNEEVQQVKSWAESRL
LLTWGDLSEDDQLMHAQNMVKYWNYCQGLVAKRKENPTDDLPGDLVRYQAEGYEISDREI
AAMCYSALFAGHETTTSLLGNGIRELLIHRKSWESLCTNHEMIPNAVEEVLRYSPSIVSW

RRRSTEEATVGGITIPAGSNILLVMGSGNRDEAQFENGEDFDIERKNANQHLSMGSGIHF
CLGAPLAKLEAKVVLEELTKRLPSLRLTPEQTFAFAQNTSFRAPVALEVEWDV

>CYP1209A2(FAES_4882)Fibrella aestuarina

MDAPIHPGLPFVGNTLEIARDPLAMFGRLFQRYDRIVKINIGGRNQYLVFRPEDAKHVLQ
ENHRNYGRSPAFLILKRFLGEGLLTSDGDFWRQQRRLAQPAFHRQKIALLGETMVQESAA
WIDELAQHDLTTPVNTSQAFMDVTMRIVCKTLFSTDVTGSDALDGLSNALDTLNRLANDS
LLSPIKWPQHWPTPRNIRFRQARERVDKLIYSLIADRQRTGERHDDLLDMLMYAEDEENG
RMSEQQLRDECVTLFTAGHETTAVSMAWTTYLLARHPDVLARLRAEVDATLGPYAPGTLP
SIAAFRAMPYTLQVVQEGLRLYPPAWAMSRMALGEDQIGPFRIPKGDTVLVSPYLLHRDP
AHWPDPDRFDPDRFLPEQEKERPAYAYLPFGGGPRLCIGNQFALLEMQILLALLVQRFDF
QPANTRLVRPKPLITLRPNRPIELHLTARP

>CYP1209A7(A6C57_23025)Fibrella sp. ES10-3-2-2

METLVPVHPGLPLLGNALSLARDPLGLFTKLHRTYGRVVRISIGGRRQYVLFQPEDVKHV
LQENNRNYVRSPAFMVLKRFLGEGLLTSDGDFWRKQRRLAQPAFHRQKITMLAETMVQES
AAWVGDLRQLDLSKPVNISQSYMDVTMLIVCKTLFSTNVEGRLDGLSHSLETLNVLANKA
LLSPIKVPRTWPTPNNIRYNQSLERVNALIYEFIHTRKQTGDRHDDLLDMLLHATDEDTG
ESMSEQQLRDECVTLFTAGHETTAVSMAWLTYLLAQHPDVVTRLRTEADAVLGDNVPGKV
PPVAAFRTMPYALQVVQEALRLYPPAWAMSRMALADDQIGPYRIPKGDTVVVSPYLLHHD
PTNWPDPDRFDPDRFAEGRDKDRPTYAYLPFGGGPRLCIGNQFALMEMQILLTFFVRTFD
FQLVNAASIKPKPLITLRPNRPIQVKLTPRS

>CYP2230B1(HME7025_02477)Allopseudarcicella aquatilis

MDGKSTIKWNPFAPDYFNDPYPIYALCRQHNPIQKDSFGNILLFRYRDIAPILDSTDFEV
SSLVTYFESKESYIFKNSPQCPFLAKTTSKWLMYLNGDIHRKLRIALGKVLFSYDFDRLI
QEAVKDSVNHFQDYQELDLVHFSKYFIFHILGQFIGLKDFASFEKVVEYSNLAARSQDIF
VSKQMYLEINQCFLWGKGLFSETGFKAKLVKELDGFEFEEDDYYSILAVTLMAFFETSKD
NLALTLHSILSNPSLKEYVLNADQKAIKLVSEECIRYTSPLQFTVRISKNEVIIHGQQFA
ASTRFVLGIASANRDEEIFESPDEILPNRKVNPHLAFGAGAHLCLGALIARKEMEFGLKP
MVEFLQKFNIDSTQPLEWGNQIFMRTLERAMVKMH

>CYP236A29(DJ013_00490)Arcticibacterium luteifluviistationis

MDKKISEFKDPFSTARKEKGIGEMDDQNDPVQMLLRLKDVRKTAHNWKTFQSGAKPGRIV
IPSEVDIRETRQLPFELDPPEHTEFRGIVEDWFKRPQNEEYAQNVKKQISSAIDDAMLKE
SFDMVTDLALPIQSRALTLLFNIPLEEAKVWISWGTHVFRSEGEALDKDKANVLYDYIDE
QLEKAAKNPGEDLYSVLLQSEYQGRKITKEEAKGVMVLTFAGGRDTIINALTNATAYFAE
HPESLNRLKEEPQLIGKAVEEFVRYFSPLTQMGRVVTEDTYVCEHAAKADTRVSLCWASA
NRDETVFENPNEIQIDRKMNPHVGFGFSHHKCMGAPHARQVMKIFIEVLTEKVGQIEILD
FKENIEELGEFERKVGFHNLQINLKPIA

>CYP1144A10(DDQ68_22250)Hymenobacter nivis

MADDDLGGRVPRWALPQVPRWVSLRNSLKLVADPLGALDAIHARFGATARLYIGGVQPSV
LTRDPGLVQHVLQKNHRNYAKSRFNLGFARYIGHGLLTNEGPDWLRQRRLIQPGFHRQRV
AGLTGLMQEIVADTLGPLAQDATRLGGAVAVPAHALMTRLTFRIIARSVFSTSFADAELD
RLAQLITELQGFYMSTIRQPYLNGWRWLRGQFRYHDARTAELRALLGRYIAQRQAASAAP
GAPAPPDDLLQMLLDARYEDTGQPMAPDRVLDEALILLVAGHETSANALTWLLYLLAHHP
AEAQAIQHETAEVLGNRAPTFADLPRLGRALHAVQETMRLYPPAWMVARVALADDDYQGL
RIPKGTLFSLNLYGLHHDPQHWDAPAEFRPARFAADAPRPLVPFAYAPFGGGPRLCIGLQ
FALTEMQLVVVELLRVFDVEWPVGQPPVAKQPLITLRPRGDFQVRLRLR

>CYP1144A6(D3Y59_07700)Hymenobacter sp. sh-6

MNASAPAASPTLPQVPRLRAFRNSLALAENPIPVLTQYLDELGDTIGLHMGGIKPTLLTR
DPGLIQHILQKNHRNYPKSEMSHGVARYLGHGLLTSEGSYWLQQRRLIQPGFHRQRIAAL
TETMLQVIEECLEPVAAQARQQGGATTVAVHELMTTTAFRVIARSVFSTSMSEAELQQLA
HLLTDIQAFYTRTLRQPYLKPWLAVKGQFRYHDQLAAQMRQLVLKYIRQRQQEGGAGKDD
LLQMLLDARYEDTHEPMTEAQVLDEAIILLVAGHETSANALSWLWYLLAQHPEVVTKLRA
EMAAALGERRPTFQDLPQLPYSLQVIQETMRLYPPAWIVDRQALNDDEYNGLPLPKGTLI
SAYIYGVHHLPRLWPEPEAFRPERFGKEQLREQPAYAYLPFGGGPRLCIGNQFALTEMQL
VLLETLRRFEVEWEAQPAPGMRPLITLRPRAEITLRFRVRA

>CYP152AP1(PKOR_18110)Pontibacter korlensis

MRKIPKERTIDNTLAVLMDGYPFLTKRMEKFNSKIFKTRLLGEEVICLYGKEAAALMYDN
SYFWRQNVIPKRVQNTLMGTNGVQMLDDEQHHHRKALFMSFMSRDKIQRLMELMLRYWRA
YAQKWEKMERVVLFDEASEVMYLAASEWAGVAVDPTKVREHAQEYINMIFGFGGVAMRYL
RGVKARNKREQEIGQVIENIRSGKLDVPKNTAAYQIAWFRDLDGELLDTKIASVELINVI
RPIVAIANYVAYSALALHEHPEQRNKLASGEDKYSQYFVQEVRRLYPFTPFLGARTRKGF
EWNGYQFDEGKLVLLDAYGMLHDPEIWPEPYEFKPERFSNWSGSPFDFIPQGGGDDFYTT
HRCAGEWITIEAMKVALKFLTLEVQYEVPQQDLSISLVQIPTKPKSGIEISNIVYKGHGL

>CYP152AP2(CA264_05980)Pontibacter actiniarum

MKNIPHDNTLDSSISLLLEGYPFLKKRMEDHQTDIFQTRLLGEKVICIHGKEAAALFYDN
AYFWREGVLPKRVQQTLMGKNGVQMLDGEAHRHRKALFMSFMSRDRIDDLMGLMLRYWRA
YARKWEKMERVVLFDEAREVLCLAACEWAGVPLKPTEVREHAQEYIEMIYGFGGATTRYW
RGIHARNVSEKWNKKIIEDIRSRKLEVPENSAAYQIAWFRDLDGELLDLHVATVELMNVV
RPIVAIATYVAFSALALHEHPEQVEKIRTGEEKYAQLFVQEVRRFYPFTPFLGARPHRDF
EWKGHQFKEGTLVLLDVFGMLHDPEMWPDPYAFKPERFTEWSGSPFDFIPQGGGDHYTGH
RCAGEWITIKAMKVALQFLTQEVKYIVPEQDLEVDIHKMPTLPKSGFEISDVQYTGSNVY
AEV

>CYP1252A5(MY04_1413)Flammeovirga sp. MY04

MNYSKTFKDLDGPKGKPFVGNIFDLEKERLHLQYEDWSRIYGKMYNLKFLSTNVTVCTDP
DINAYILKNRPTKFRRLKKLADVINEVGVEGVFTAEADSWRKQRKVTQKALDKRHIKTFF
PKILLVASRLENYWNEQLKIKKSHEDLPKDFIRATVDVTTNLAFGYDMNTVENLENITQK
HVEKIFPKMNQRVNSPIPFYKYFKSKSDKEFDESMSYLREMLGEIIKITKEKLNDQPELI
EQPTNFLEAMIASQEKDNPFTWDEIFGNLYTMLLAGEDTTSNTLTWTSYFLSKYPDVQNK
IREEIALTLPNGEMNDIDQLKNLRYVNAVMKEVARLKPVTPNLYMQANEDIVINDILFPK
GHFFITQLSFASRSEEFFEDALEFKPERWLTEPSVNKVCPFSGHMKPDAAKPFGGGPRLC
PGKYLAEVEFAVFLVTLMKNFELGFAQSEDKVEEEFAFTMVPKNLHIVIKKIKTPQILRI
KNWIILTLIIIILRY

>CYP236A22(MY04_2085)Flammeovirga sp. MY04

MEHQTETQKKVKTSEFPDPFEQARIERGYGDIDDQNDPVTMLLRLKDVRKTAHNWKTFIS
GATPGRIVVPSEVAIRDIRQIPFEVDPPEHKDYRNLVEPWFKRPLEEEYTAKMTKIIEEL
VDEVIAKDDIEVVSDFSLKLQSRCLTLLLNIPYEEAETWIGWGVHVFRSEESSTDADKAN
ILFDYLDEQIDRAAENPSDDLYSLLLSSEVNGKKMTKEEVKGVMILTFAGGRDTVINAVT
NSLAYFAEHPKSLERLRQEPEIVGKAVEELVRYFSPLTHMGRVVTEDTKVCEHAIKNDSR
VSMCWASANRDSAAFENPNEVVLDRKINPHVAFGFSHHNCLGATHARQILKVLINTLANK
VGSIEIKNYKENIEEWGEFNRKVGFDNITMNIKGK

>CYP1252A3(EI427_08965)Flammeovirga sp. L12M1

MTNQDYTRTITDIKGPKGIPLFGNLFQIEREQIHLYYEKWAKEFGDFFFVNFLGKKILIS

SNPQNNANILKNRPTKFRRLSKMAEIIEEVGFYGVFTAESEEWIKHRKVTQQALSNKNVK
SFFPQIVKVAERLDNFWESNLIDEVTKYQISNDFTRATVDVTTNLAFGYDMNTVENHENE
IQDHIAKIFPKVNERVNSPLPIWRYIKSSSDKGLDQSMDALKITIGEFIKKAENNLEKDP
SLKENPSNFIEALIASQDKENPFTWDEIFGNIYTMLLAGEDTTANSLSWLTYFIASDNEL
QNKVYNEIKEVLGEKEQITTFEEVARFKYLSAVLKEVLRLKPVSPSLYFQANEDVVVGDL
LIPKDTFVLTQLRVGALSDDNFENAAEFIPERWLSAKETTGGCPFTGKHKAEVAMPFGGG
PRFCPGKFLSETEMILFAVTLFKKFELTLSVPKEDIKEKFAFTMSPENLAVNLKKRKQVS
ISNLTKEKVSI

>CYP1252A4(EI427_08970)**Flammeovirga sp. L12M1**

MELTKDVHYKQTINDLKGPKGLPLVGNLFQLDKIKIHNQYEDWADEFGELYALNFLGRKV
VVSTSPINNDFILKHRPTKFRRFSKMAEVIENVGIDGVFSAEGDVWKKQRQVTQKALDSK
NVKSFFPKIVLVANRLEEYWNTLIASSQNKNHEIMHDFMRITVDITTLLAFGYDMNTINN
KTDPTQEQIAKIFPKINERINSPLPFWKYIKTTSDKEFDQALAHIKSFFGDFISNTNQKI
EDNPELLENPSNFLEAMLASQDKENPYTWEEIFGNLYTMLLAGEDSTSNTLSWVSYFLAS
KKDVQIKVQKEISDLLAGNVELKSFDQLKLFKYTSAVIKEAMRMKPASPNLFMEANEDVV
IGDVLFPKNSLIITQLSKSARSEDHFENSQDFLPERWMQKEEGCPFTGRHNEKALKAFGG
GTRTCPGKLLAETEILVFIITMMKNFDIKLSVPEKEIIEKYAFTMSPKNLFVEITPKEKK

>CYP152A24(D770_07040)**Flammeovirgaceae bacterium 311**

MPKAINFRLTIDFSHQPIIAFFTQDLPMNNIPKDKNPDSTLSLMLEGFPFIRNRCQRYGS
DIFETRLMLQKTVCLHGEEAAKIFYDPARFKRKGAAPKRIQKTLFGEKGVQTLDGEAHRH
RKAAFMSLMTRDHIQLLMQMLENNWRLYIHKWETVDSLVLYDEVQELLCRASSKWAGVPM
QESEVKNRAKDYGLMVDAFGAIGPRHLRGRLARTRAENWMKGIVKQIRSGQLTPEPGTAA
HLFSWHQDLKGKLLPEKVAAVEIINIIRPIVAIARYITFSALAMHEHPEYRQKLQTGEAD
MEEIFAQEVRRFYPFGPFLGARVKKDFEWGGYHFPKNRLVLLDIWGTNRDEKQWEEPDRF
WPERFRNWNGSPFNFIPQGGGDFMANHRCAGEWITIEVMKQAINILANKINYRVPEQDLG
FSLVRMPTIPRSRFVISDVKVK

>CYP1103A11(GFO_0170)**Gramella forsetii**

MNNNNKIPEVSLLKFLKHSANILKNPLPFHHQNFTEKGDTFRLNIGFKKSVIFSRDAGFL
EYALQKNQRNYIKSEIQTKDLAKYVGKGLLTSDGEHWKKQRKLIQPAFHKKQLANLLGSI
KEAINTEYDKIQTDKEIDIFPILNDLAFQTVVKSLFSSAANQEEINRLQYITESAQKMLV
KELRQPYLGWWFKASGKIDSYLKLTAEARTILKRIVHERRESNTRYDDLLDMLLDAKYDD
GNFMDEEQLIDEILILFTAGHETTSNALTFISQLLALNPQWQDRILGEIQNLNTETDDLM
GFVTQAKVTQQVIEEGMRLYPPAYFIDRVNVEQDEFGGMLLEPGENLLFSVHEIHRHPNL
WEKPEEFLPERFEDGGKKYSSQYFPFGAGPRKCIGNNFAMFEMIIAVSELVSRFKIISVA
DEIDIKPLITLKPKNAILKFTKR

>CYP1103A12(LPB144_08270)**Gramella salexigens**

MGESRKIPEVPLFRFLKHSANILKNPLPFHHRNFEEKGDTFRLKIGYGTSVIFSRDAGLA
EYVLQKNQKNYIKSEIQTKDLAKYVGKGLLTSDGEHWKKQRKLIQPAFHKKQLANLLNSI
KEAIHAEYQKIETDKEVDIFPVFNDLAFQTVVKSLFSSAANQEEINRLQYITEAAQKMLV
KELRQPYLGWWFKMSGKIDKYLDLTNEARLILKRIVAKRRNSEERYDDLLDMLLDARYED
GEFMDEEQLIDEILILFTAGHETTSNALTFTTELLALHPEWQEKIFKEVSELKKKDEDLM
SLVTGAKYTQQVLEEAMRLYPPAYFIDRVNVERDEFEGKEFKAGSSLLFSIYEIHRHPAL
WDRPDDFLPERFEEGGRQYSSQYFPFGAGPRKCIGNNFAMFEMIIAIGELVSRFKITHIS
GAIDIKPLITLKPKNAILKFKKR

>CYP1103A15(GRFL_1964)**Gramella flava**

MGKNDHIPEVSLFRFLKHSLEILKNPLPFHHQNFEQKGDTFRLNIGPGKSVIFSRDPALA
EYVLQKNQKNYSKTEIQTKDLAKYIGRGLLTAEGDHWKRQRKLIQPAFHKKQLAQLLDSV
QRAIQQELRRIEPEKSIDIFPIFNDLAFQTVVKSLFKDAASKEEINRLQFITEEAQKMLV
RELRQPYLGWYFKLSGKLESYLALTRESREILKKIVHDRRESGKKYDDLLDMLLDARYED
GNFMDEEQLIDEILILFTAGHETTSNALSFIAQLLAHHPEWQDKIFAEYAKLSSENDLMS

LVTQTPSTQMVIEESMRLYPPAYFIDRVNIEKDEFQNMKFEKGSNLLLSIYEMHRHPDFW
EDPDDFKPERFGGNSMKYSSQYFPFGAGPRKCIGNNFAMFEMILAVLEIISKYKIKPVKP
QIDIKPLITLKPKNAILKFEQRS

>CYP1103A16(C7S20_08530)Gramella fulva

MKQNELSMKKENNIPEVPLSKFLKHSANILKNPLPFHHENFEEKGDTFRLNIGIGKSVIF
SRDAAFAEYVLQKNQKNYIKSTIQTKDLAKYIGRGLLTSDGEHWRKQRKLIQPAFHKKQL
AKLLGAIKQAILEEIPKLKTDETFDVFPFFNDLAFQTVVKSLFSSAATQEEINRLQFITE
SAQKMLVRELRQPYLGWWFKISGKIEHYLKLTRESRQILQKIVDERRRSDEKYDDLLDML
LEARYDDGNSMDDEQLIDEILVLFIAGHETTSNALSFISQLLALNPEWQDKIFEETQNQA
SEDLMAVVTNSPVTQQVIEEGMRLFPPAYFIDRVNIEDDEFNGKFFPAGSNLLFSIYEIH
RHPDLWQRPDEFLPQRFSENPKQYSSQYFPFGAGPRKCIGNNFAMFEMIIAVQEIISKYT
ILPVKDEIEIKPLITLKPKNALLKFMNRT

>CYP1099A4(Fjoh_1643)Flavobacterium johnsoniae UW101

MSEKQKHTYPEKLSILRFFRDAEGVRRNPIPFHKKYFDKLGDSFSIRIGFSKYIILSRDN
EIAQYILVKNQKNYHKSKFQSVYLSKYLGKGLLTSDGDFWLKQRRLIQPAFHKQKMNQLV
DNMNAVIALELENLIEEKPIDLFPVMSNLAFNVVAKSLFQLSTAENKFQRIKFIIEEVQN
FLIKEIRLPHKAWWFSLSGQVKKHLKLAEENNHIIQEIIEERKASGEEINDLLNMLLETR
YEDTGESMSVEQLIDEIKVLFIAGHETTANALTFTLHLLGRNPEVQQKIFEEIIEIESQT
DNVIEQLQKMTYTNAVLNESMRLYPPAWITDRQNLEDDSLAHFKIKKNTLIGVSFYELHR
NPKYWKNPDEFIPERFLGDQKKESMQYFYPFGAGPRMCIGTGFAIYEMCLTIAQVVKKYI
IKSNNDVIQFNPLITLKPVNVEVSFFKR

>CYP1103A18(ZPR_0586)Zunongwangia profunda

MILIMKREIPEVSTLTFLKNAARIVKNPLPFHHENFQKHGDIFRLNIGPKKSVIFCRDAE
FAQYVLQKNQKNYIKSEIQTKDLVKYVGNGLLTSNGEHWQKQRKLIQPAFHKKHIANLLD
TVLEAIRVEYVKIRAGKTIDIFPVFNDLAFQTVVKSLFSSAASQEEINRLQYVTEENQKM
LVKELRQPYKRLWFSLSGKLKYHLKLSEESRLILRNIVHKRKQNPKRYDDLLDMLLDARY
EDGQPMREEQLIDEILILFVAGHETTSNSLSFTTQLLAQNPQIQDKIYSEVKVASEATTS
LMEFVQGCPYTQNVILESMRLYPPAYFMDRVNLEEDEFNGIKLDKGSDLLFSFYEIHRSE
KHWEQPLEFNPDRFTSEKNPMAYFPFGAGPRKCIGSNFAMYEMILAVSELILNFKILSVK
EDIEILPLITLKPKNAYVEFEKRA

>CYP152AQ1(ZPR_3345)Zunongwangia profunda

MVEESSIGFWFKGYDYFLDQFNATKNNVLKRRLLFKKYYILRGEEATALFYDQERFSRHK
ATPKRFKKTLFGLNGVQGLDGEQHKARKQLFMHCMNVDSLNQLNTYFDEAWFEAVEDWKA
KNTINYFDEMERILFKCACKWIGLPFTKKDIVNQVPRISRMISSAGKIGIQHWRGRSARK
ATENWVKSLIKNYRNKKKNSDTIFDHFIGYKNPKGKHLPDQIVAVEILNLLRPIVAVARY
MTFNLKALEDYPSYKEKLRENYEEYGENFIQEVRRFYPFFPVISGRIRKNFEFKGHKFTK
GQRVLLDLYATNHYAKLWKAPERFYPERFMNAQISPFNLIPQGGGDHYKNHRCAGEWITI
NLMKRNLYFLIYDINYTVKSKNLRIKKNQFPPLPANGIKISKITKLRLME

>CYP236A34(YQ22_01795)Maribacter sp. 1_2014MBL_MicDiv

MFSDAFKDTRVSKGTCPFNDQDDPVTMLLRFKDVRKTAHNAKDFSSAAKPGRIVVPSEVN
IRDARQIPFEVDPPEHTEYRALVEDWFKRPLDIDYEFKLTRIVEAEIDRVLQKGAIEAIS
ELSLPIQSKALTLLLNISLSEAETWISWGTHVFRSEDSDLDADKASILYDYIDAEIDKAI
AAPDDSLYSVLLNSEYQGRKMTKEEVKGVLILTFAGGRDTVINYLTNTISYLAEHPDAIE
KLNDNPALVNTAIEEFVRYFSPLTHMGRVVKEEQEISGHIAKEDTRVSLCWASANRDESI
FENANKIVLDRKMNPHVAFGFGAHKCLGATHARQLMKVFLRVLTEKVSTIEIIDAVENIE
DWGAHKRKVGFDKLTIRLNTK

>CYP236A24(EJ994_03085)Maribacter sp. MJ134

MDESVFKDPFKTARIEEGIGHMTDQNEKVKMILRLKDLRKTAHNWKTFQSGGKEIGRIVV
PSEEKIRKTRQIPFEVDPPEHGQYRELLEAWFKRPLDSEYEEKLRVQIATIIDSVIKKDR
VEIVSELALLLQSRALTLLLNIPLEESEVWINWGTHVFRSEDTALDKDKANILYDYIDHQ
IDKAIANPGEDLYSVLLDADFQGRKLNKEEIKGVMILIFAGGRDTVINAITNTIAYFAEH
PEALRRLNEEPDVIGKAVEEFIRYFSPLTHMGRVTTEDTQICEHAVKANSKVSLCWASAN
RDETVFEKPNDILLDRKMNPHVGFGFGHHKCLGATHARQLLKILISVLVQKIDNIQILDY
KENIEDLEQFERKVGFDNIHVKFNSIQ

>CYP1103C14(EJ994_09855)Maribacter sp. MJ134

MKKLPVVSRTHVFKNRKQILKNPIPFHHDNFNRLGDTFEVAVGLKKRIIFTRQAKFIKQI
LQTKQKHYQKSSLQTVDLAKYIGRGLLTSNGEHWRNHRRMIQPAFHKRKLQGLLQIMYQA
IKGELSKIKAGEIQDIYPLMGDLAFQVVATSLFSRDDIQVQMSALKEITEANQRMLIQEM
RQPYLNWWYKFSGKINRHIKLSLEARAILSSIIKERLAQDSEKDDLMDMLLKATYEDGSR
MPEKQLIDEILILFAAGHETTANALGFILFLLAKHPEKQGKAFAEINAIDWTEGIEMESL
RKLGYVKQCIEEGMRLYPPAYYIDRESIIKDEIEDYEIPENSMLLLAIYELHRDSRFWES
PNAFKPERFDPANKRDYSDYYFPFGGGPRMCIGNNFAMQEMILTVAEILRSYTLRPVDDE
VLINPLISLKPVSVKLHFIPR

>CYP236A26(IX49_12470)Cellulophaga lytica HI1

MAKCKFADAFKEEREKSGIGKMDDQGDPVVMLLNHKDVRKAAHNYKAFSSEAVPGRIVVP
SEVNIRTTRQIPFEVDPPMHKEYRSLLEAWFKRPLQDTYKEKLTTIIGTVLDDVLSKDKV
DVVSDLALVIQSRALTLLLNVPFDKSVKWISWGTHVFRSEGEALDGDKAAILYNYIDEEI
DNAIANPTDNLYSTLLAAEVNGKKLTKEEVKGVLILTFAGGRDTVINALTNMMSYFSANK
KALDYIRSKPEIIKTAVEEFIRYFSPLTHMGRVVTEDTTVCEHAVKADSRISLCWASANR
DEKVFENPDEVILERKVNPHVGFGFSHHNCLGATHARQIMRITLKLLAEKVSDITVLEAE
ENIENLGEFTRKVGYNSLKATFKKR

>CYP1103C15(M667_07630)Cellulophaga baltica NN016038

MQKTPIVSQFEVFKNSRRILKNPLPFHRENFEKYGDVFRVKIGVKEELAFTRSPQLIKHI
LQKQHKKYHKSSLQTKDLAKYIGHGILTSNGEHWRTHRRMVQPAFHKKKLQNLMATIREA
ILLEIKNIPVTHPIDVYPIMSNLAFQVVAKSLFSRDDIQEEMSQLQYITETNQRMLIKEM
RQPYLNWWFQLSGQIRKHLNLAKDGQAILHQLIEGRRNSAVEKDDLLDMLLQARYEDGTA
MAKEQLIDEVLILFTAGHETTANALSFLVFLLAKHPEIQEKAYQEMKNITLEGDDVLIQL
LELKYVQQCIEEAMRLYPPAYIIDRVAIEDDEFEGLNIPKDSLVLMSIYELHRNENFWEA
PNEFNPERFDASKKKEYQEYYYPFGAGPRMCVGNNFAMYEMIIAATELLQKYEMTTALPE
VTINPLISLKPENVLISFTARN

>CYP236A32(M667_11705)Cellulophaga baltica NN016038

MKESIFTDPFKKARQETGIGEMDDQNDPVAMILGHKEVRRAAHNWKSYSSGAIPGRIVIP
SEVNIRSIRQIPFEVDPPQHGAYREVVEKWFKRPLEKKYEEELTLQIEALVIEVLEQDSF
EVIHDFALVLQSQALTLLFNVPFEEAQTWISWGTHVFRSKDSALDGAKANVLYDYIDQKL
AESSSNPGKDFYAVLLAAEVDGKKLSAEEAKGVLILTFAGGRDTVINAVTNSIAYFAEHP
KSLEWLRKEPELLPTAIEELIRYFSPLTQMGRVATQDTMVCQHAVKADSRISLCWASANR
DETVFENADEVVLDRKANPHVAFGFGTHNCLGATHARQVMKVLLRTLIAKVKSIDIHDAK
DNIEDLGDFKRKVGFDSLTVKFNKI

>CYP1103C15(M666_07665)Cellulophaga baltica 18

MQKTPIVSQFEVFKNSKRILKNPLPFHRENFEKYGDVFRVKIGVKEELAFTRSPQLIKHI
LQKQHKKYHKSSLQTKDLAKYIGHGILTSNGEHWRTHRRMVQPAFHKKKLRNLMATIREA
ILLEIKDIPVTHPIDVYPIMSNLAFQVVAKSLFSRDDIQEEMSQLQYITETNQRMLIKEM
RQPYLNWWFQLSGQIRKHLNLAKDGQAILHQLIEGRRNSAVEKDDLLDMLLQARYEDGTA
MAKEQLIDEVLILFTAGHETTANALSFLVFLLAKHPEIQEKAYQEVKNITLEGDDVLIQL
LELKYVQQCIEEAMRLYPPAYIIDRVAIEDDEFEGINIPKDSLVLMSIYELHRNENFWEA
PNEFNPERFDASKKKEYQEYYYPFGAGPRMCVGNNFAMYEMIIAATELLQKYEMTTALPE
VTINPLISLKPENVLISFTARN

>CYP236A32(M666_11750)Cellulophaga baltica 18

MKESIFTDPFKKARQETGIGEMDDQNDPVAMILGHKEVRRAAHNWKSYSSGAIPGRIVIP
SEVNIRSIRQIPFEVDPPQHGAYREVVEKWFKRPLEKKYEEELTLQIEALVTKVLEQDSF

EVIHDFALVLQSQALTLLFNVPFEEAQTWISWGTHVFRSKDSALDVAKANVLYDYIDQKL
AESNSNPGKDFYAVLLAAEVDGKKLTAEEAKGVLILTFAGGRDTVINAVTNSIAYFAEHP
KALEWLRKEPELLPTAIEELIRYFSPLTQMGRVATQDTMVCQHAVKADSRISLCWASANR
DETVFENADEVVLNRKANPHVAFGFGTHNCLGATHARQVMKVLLRTLIAKVKSIDIHDAK
DNIEDLGDFKRKVGFDSLTVKFNKI

>CYP1103A9(Krodi_1564)Dokdonia sp. 4H-3-7-5

MPDKIPAVSVFKFLANAGSILKNPLPFHKENFESKGDTFKLKLGFGNDVVFSRDPEFAKY
ALQKNQRNYSKSPIQTKDLAKYVGEGLLTAEGDHWKKQRKLIQPAFHKKQLAQLLDAMHE
VIKEELLRIETHKPFDVFEVFNDLAFMTVVKALFKTDVDRDKINRLQHITEQAQKMLVKE
LRQPFKAWYFKYGGAIDKHLALTKEARVILKELVKERKDSGDKPGDLLDMLLDSQYEDGG
FMDNKQLIDEILILFSAGHETTSNALTFTAELLARNPIWQDKIYEEYAFAKANSTNLNEF
LRHCTITKQVLEETMRMYPPAYFIDRVNIEDDDHNGMEVPAGSNLLFSVIEIHKHKDFWE
RADQFDPTRFDDNAGMKHPAYFPFGAGPRMCIGNNFAMYEMILAITEMVSMYKITPKNTP
IEILPLITLKPKNALLEFIPR

>CYP1103A8(MED134_11416)Dokdonia sp. MED134

MSNDIPAVSVLKFLANAGSILKNPLPFHHDNFESKGDTFKLQLGFGNEVVFSRDPGFAKY
ALQKNQRNYTKSPIQTKDLAKYVGEGLLTSEGSLWKKQRKLIQPAFHKKQLAQLIEAMHT
VIKEELQSIKTGEAFDVFEIFNDLAFNTVAKSLFQTEVDRKKINRLQYITEQAQKMLVKE
LRQPYKSWYFKYAGPIDKHLALTQEARDILMELVEERRASQQKVGDLLDMLLESTYEDGT
GMDDEQLIDEILILFTAGHETTSNALTFAVSLLARHPEWQDKIYEEYAFAKAYSTSLNEF
LRHCTVTKQVLEETMRLYPPAYFIDRVNIEEDEYMGMKIPAGSNLLFSCIEIHKHNDFWE
DPLQFNPTRFDDNAGMYHDAYFPFGAGPRMCIGNNFAMYEMILAVTEMVSRFKITPINTP
IEILPLITLKPKNAILEFTAR

>CYP1103A9(DCS32_04110)Dokdonia sp. Dokd-P16

MPDKIPAVSVFKFLANAGSILKNPLPFHKENFELKGDTFKLKLGFGNDVVFSRDPEFARY
ALQKNQRNYSKSPIQTKDLAKYVGEGLLTAEGDHWKKQRKLIQPAFHKKQLAQLLDVMHE
VIQKELLRIVMHKPFDVFEVFNDLAFMTVVKALFKTDVDRDKINRLQHITEQAQKMLVKE
LRQPFKAWYFKYGGPIDKHLALTKEARVILKELVQERKDSGDKPGDLLDMLLDSQYEDGG
FMDDKQLIDEILILFSAGHETTSNALTFTAELLARNPIWQDKIYKEYAFAKANSISLNEF
LRHCTITKQVLEETMRMYPPAYFIDRVNIEDDDHNGMEVPAGSNLLFSVIEIHKHKDFWE
RADQFDPTRFDDNAGMKHPAYFPFGAGPRMCIGNNFAMYEMILAITEMVSMYKITPKNTP
IEILPLITLKPKNALLEFIPR

>CYP236A2(ZOBELLIA_4677)Zobellia galactanivorans

MKKSELPDPFEKARESKGYGEMNDQDDPVTMLLRHKDVRKSAHNYKTFQSGAVPGRIVIP
SEVDIRDTRQIPFEVDPPVHGVYRAIVEPWFKRPLQAEYQEKLTAQISEIVEETLLKGSV
EVVTDFALRLQSRALTLLLNTPFSESETWISWGTHVFRSEGEALDGDKANILYHYIDEQI
DRASENPGDDMYSVLLNSEFEGRKLTKEEVKGVMVLTFAGGRDTVINAVTNSIAYLAEHP
EALERLRKEPEITGRAVEEMIRYFSPLTQMGRVVTEDTHVCEHAVKADSRISLCWASANR
DAAVFENPNEIVLDRKVNPHVGFGFSHHNCLGATHARQILKILLQTLAQKVASFEILDYK
ENIEDLDHFQRKVGFHNIQIKFNPLTK

>CYP1103A10(Aeqsu_0891)Aequorivita sublithincola

MKAKKIPSVSAFRFLTHSIQILKNPLPFHHKNFETKGDTFRLKLGFGKSVIFSRDAGLAQ
YALQKNHRNYTKSPIQTRDLAKYVGHGLLTSEGELWQKQRKLIQPAFHKKQLINLLDTIN
SAIKLELTKIETGKPKDIFPVFNDLAFQTVVKSLFSSAVNQKEINKLQNITEAAQQMLVK
ELRQPYLIWWFKLSGTIKKHIAETEEARAILMKLVYERRNSGKREDDLLDMLLDARYEDG
SVMEDRQLIDEILILFTAGHETTSNALTFTCELLARNPDIQEKLFEEVIFAEVNSETLMD
FIKNLSFTKNVIEESLRLYPPAYFIDRVNIENDEFDGMFIPKNSNLLFSLLEIHTNPANW
EEPQKFRPERFSDVNPNHFSGQYFPFGAGPRMCIGNNFAMYEMILAIAEIIKTYKIAEKK
TSIEMKPLITLKPKNAILEFNFR

>CYP1144A9(P700755_002170)Psychroflexus torquis

MTSSTHFPEVPVIEFLKHAGNILKNPLPFHQKNFEALGNTFRLNLGLGNSVVFSRDPEFA
IYALRDNQKNFKKTDIQTKDLAKYVGHGLLTSEGQKWKSQRKLIQPAFHKKNIYSLLDVM
VEAIDKELERIEVDTPIDIFPIFNDLAFKVVVKSLFSDAINAEEISRLQYITEETQKMLV
KELRQPYKKWWFVLSQELKRNLNLSREARDLLIRIIKRRQNSNSESKDLLDMLLALTYED
GSKMNQEQLIDEILILFIAGHETTSNALSFTVQLIGQDEHVQEKLVSEIDLLEDHSFFDI
LKDSKYTENVIQESMRLFPPVYFIDRQNIERDTFKGFEIPQGTTLLFSVHQIHRNATNWE
NPNKFMPERFQDSRSVSNFYFPFGAGPRKCIGNNFAMYEITLAINRLLKTYKIEQVNKEI
EIQPLISLKPKNAIVKFSKR

>CYP1103B3(DDD_1081)Nonlabens dokdonensis

MKQIEQVPTLTFLAKSRQIYKDPLPFHRENFKKYGNTFKISPKPGLLIHFTCDEKLTQHI
LQKNQKNFNKSTLQTEDLGKYIGHGLLTENGEKWRANRKLIQPAFYKKSISSLMNVMDEV
IQEEIGKIKEDVATDVYEIFNDLAFKVVARSLFDFADIDNLDSKISRLQYITEKAQKTLI
KELRIPWMKWYFDREWLSGKKSIPHSLSLIEEAREILRNIINNRRSGNKEPGDLLDMLLH
STYEDGSHMEDDQLIDEILVLFIAGHETTANALSFAAQLLAQHPETITKATQELQHLENE
NLMEGLMSMPYIKQCVEETLRLYPPAYVTDRVALEADSCEDVEIEKGSIWLVSFYEMHRR
QDLWERPEEFIPERFEKEKAKSYRDFYFPFGAGPRMCVGNNFAIFEMVLTSARLLERFEV
KPVHDTIDYHPLITLKPRNAQLIFKRK

>CYP1103B5(AAT17_08830)Nonlabens sp. MIC269

MEIPQVPFIEFFSKSRQIYKDPLPFHRYNFKKYGKTFKIKPKSGLVIHFTCDEDLAQYML
QKNQKNFHKSTLQTKDLGKYIGHGLLTANGEQWKANRKLIQPAFYKKSIATLMNKMEEVV
DEELGKIKPNETQDVFEIFNNLAFKVVAKTLFYIEDIDDKINRLQEITEKAQKMLIKELR
LPFLLWYYKREWLSGSGSIPYHLELINEARKLLSDIIDDRKTSQKHFGDLLDMLLHSTYE
DGSYMSKEQLIDEILVLFIAGHETTANALSFATELLAQHQNAQDLVLQSLQDVDKESDLM
TQIMRSQFTKQCVEETLRLYPPAYVTDRVALKDDECGSIKIDKNTNWLISFYELHRREDL
WESPDDFKPERFNPDKVKEYKNFYFPFGAGPRMCVGNNFAMFEMVMVLNKLVSKYHLVPT
RERIEYNPLITLKPKKAIVMFKER

>CYP1103B2(CW736_02995)Nonlabens sp. MB-3u-79

MRKIEQIPALTFLAKSRQIYKDPLPFHRENFKKYGNTFKISPKPGLMIHFTCDEKITQHI
LQKNQKNFNKSTLQTEDLGKYIGHGLLTENGEKWRANRKLIQPAFYKKSISSLMRTMDEV
IQEEFSKIKTDQAIDVYEIFNNLAFKVVARSLFDLGDIDDLEEKISRLQFITEKAQKMLI
KELRIPWMKWYFDREWLSGDNSVPHALELIEEARDILRNIINNRRSSDRESRDLLDMLLH
STYEDGSYMEDDQLVDEILVLFIAGHETTANALSFAAQLLAQHPETIAKASKEISHLDNE
DLMTGLMAMPYIKQCVDETLRMYPPAYVTDRVALEADSCEDIAIEKGSIWLISFYEMHRR
KDLWKDPEVFNPDRFAPGKAKEYRDFYFPFGAGPRMCVGNNFAVFEMILVIARMLERFDI
TPVHDFIDYHPLITLKPRNAQLLFKKKVTTDY

>CYP1103B4(EJ995_01145)Nonlabens sp. MJ115

MRKIDKVSAFRFLSKSAQIYKDPLPFHRENFQRYGHTFKISPKPGLVIHFTCDTTLTQHI
LQKNQRNYHKSSLQTDDLGKYIGHGLLTANGENWRKNRKLVQPAFYKKQLATLMDSMNKV
ILKELDRIKPDIEVDIHEIFSDLAFKVVAKSLFYLEDMDERINRLQHITEQAQKMLIKEL
RLPFLMWYYDRQWLSGNSSVAYHLKLIDEAREILQDIIDQRRKEQKEYGDLLDMLLQSTY
EDGTHMSDRQLIDEILVLFIAGHETTANVLTFATQLLAHHPDEAALARDQASQVSTPDLM
TQMKELDHIKRVLEETMRLYPPAYVTDRVAVDDDVCGDIELKKDSIWLISFYEMHRRKDL
WDSPDDFVPQRFDADKRKSYSDQYFPFGAGPRMCIGNNFAMFEMMLVIKNILIKYKLEPV
GDTIEYHPLITLRPKNAQVVFKSR

>CYP236A31(BW723_04380)Polaribacter reichenbachii

MKVSEFPDAFKKEREEKGYDSIEDQNDPAMMILGHKDVRKCAHQWQTFQSGAKPGRIVIP
SEVNIRETRQIPFEVDPPEHKSYRNLLEAWFKRPLNEDYKNQLTTQIESIVDEVLNKDAI

EVVGDFSLPLQSRALTLLLNIPYSEADLWISWGTHVFRSEDTALDADKANILYNYIDQEI
DKAIENPSESLYSQLLNSKVNGKKLTKEEVKGVMILTFAGGRDTVINAVTNSIAYFSEHP
NSLKALKENPAMINKAVEELVRYFTPLTQMGRVATKDAEVCEHAVKADSRIALNWASANR
DERVFENPNEVVLDRKVNPHLSFGFGIHNCLGATHARQILRILLATLSKKVTSIDVLEAK
ENIEDLGNFKRKVGFDSLKVQFNK

>CYP2728A1(EAG08_11490)Chryseobacterium sp. 3008163

MISKTMYAYAPEIDSPYRKNVLHGLAARNLKFEKDEMKASAEKFLKECLEKGKFDFINDF
AHQYTFETISRIIGIPEEDIAELTEVVSKLNKGYLKLINNLETSDPAEQDFILFLRKFIQ
KKENSLGKDLASALIESCKVTDENSDFALCLLIFLFYAGHDNMMNFLGNGFLSLYNNPHV
FNELKGKPHFIQNAIDELLRYDSPVQFFTMYTKNDIVIRDTKISAGCQLLVCAGSANRDP
DKFQNPDEIKIDRSPQHLSYGFGAYRCIGARLAQLQSATAFEVLIQNLNIEDLKISNPIW
KNEQYIQRGPKKIIVNTK

>CYP1103C7(SB49_14710)Sediminicola sp. YIK13

MKLVSRFQVLKNARRILKNPLPFHHENFEAHGDSFKVQLTTKETVLFTRNPGLIRHVLQK
QHKKFEKSPLQTVDLAKYIGHGILTSNGEHWRVHRRMVQPAFHKKKLHNLMGVIREAIRF
ELQRIKPNTEEDVFPLMGDLAFQVVAKSLFSSTDIRDKMSQLQHITEENQRMLIKEMRQP
YLRWWYKLNGKIDRHLAMAKEGQKLLLEIIEERRTAGLEKDDLLDMLLKARYEDGSPMSD
EQLIDEVLILFTAGHETTANALSFTLFLLASHPEIQEQVYQEVSKVDFEDPEIDLMKGAM
QLQLVKQCLEEALRLYPPAYVIDRIAIEDDSFEGISLPKGTLVLMSVYELHRYSDFWEKP
SEFIPGRFNTTDKKDYSDYYYPFGAGPRMCVGNNFAMYEMILAVAEIIKKYKIKTSLEKV
EINPLISLKPKCVPLLFEER

>CYP236A21(TJEJU_2941)Tenacibaculum jejuense

MKSEFSDPFEEARKKTGLGHIDDQNDPVAMILRHKDVRKTAHNWKTYQSSAVPGRIVVPS
EVNIRDIRQIPFEVDPPLHKDFRDLLEGWFKRPNREEYQKTLKNQVSNLIDDVLSEDEIE
VVHDFSLKLQSRALTILLNTEYSESDTFISWGTHVFRSEGDSLDASKANVLYDYLDAKIN
GARENLGEDLYSVLLQAEVNGKKLTHDEIKGIMILTFAGGRDTVINAVTNTVAYFADHPK
SLLDIKNNPEKINKAVEELIRYFSPLTHMGRVVTEDTQVCEHAVKNDTRVSLCWASANRD
ETVFENPNQVNFDRKINPHVAFGFSHHNCLGATHARQIMRTLLQLLADKVSSIDIIDSRE
NIEEWGEFKRKVGFHELKVKFNK

>CYP1103A14(CW732_00785)Olleya sp. Bg11-27

MKNNIPEVSLLKFIKHSLEILKNPLPFHNRNFEEKGDVFKLNVGFNSKIYFSRDAGLAQY
VLQKNQRNYVKSKIQTVDLVKYVGEGLLTSEGDKWKKQRKMMQPAFHKKQLQNLLTGMQD
TIISEFSKVETNKTIDLFPLLNDLAFQTVVKSLFTQAANSKDMERLQCITEANQRMLVKE
LRQPYLGWWFKIGGALKKHLKLSEEARTILKGIVAERKASGHRFDDLLDMLLETKYDDGK
GMSETQLIDEILIIFTAGHETTSNALTFTFQLLAKHPEWQDKIYDEWSELGGDDVDLMTR
VSTSKICQQVLEESMRLYPPAYFIDRVNVEADRFNDMVFEPGCNLLFSVYEIHRHPKLWT
NPESFLPERFEDGGRQFSAQYFPFGAGPRKCIGNNFAMFEMIIAVTELVKNYKIRPEFDK
IDITPLITLKPKNAFLRLEKRN

>CYP1103A17(DZC78_08210)Olleya aquimaris

MKKIPEVSLLKFIKHSLEILKNPLPFHNKNFTEKGDIFKLNVSFNSKIYFCRDAAFAQYV
LQKNQKNYTKSNIQTKDLVKYVGEGLLTSEGDKWKKQRKMMQPAFHKKQLQNLLSGMQDT
IVEEFNKIYTNKTIDVFPILNDLAFQTVVKSLFTKPARAKDMERLQFITEANQRMLVKEL
RQPYLGWWFKTGGELKKHLKLSEEARIILKGIVADRKASNKRYDDLLDMLLETKYDDGQG
MSEQQLIDEILIIFTAGHETTSNALTFTFQLLAKHPEWQDKIYNEWLTLGGDDADLMTRV
SNSKICQQVLEEAMRLYPPAYFIDRVNIKPDDFNEYHFESGSNLLFSVYEMHRHPKLWDQ
PDTFLPERFKDGGRQYSAQYFPFGAGPRKCIGNNFAMFEMIIAVTELLKKYKIKPKSETI
EITPLITLKPKNALLRFQKRD

>CYP1099C2(C1H87_09385)Flavivirga eckloniae

MPKYKYPNKVHFFKFLIQASSIAKNPMLFRNKWFNEIGDTFAIKSPFYGHIILTRDAVIT
KHMLQKKHKVYHKSKIQTLYLSKYVGYGLLTSSGDYWLKQRRLIQPAFHKEKIQNLVEII
NKAVNEQVESINAEGFVELYPIMNELAFEVVAKSLFNFSAEKETLKRLQFIIEKLQLFIV
KELRMPYKKLWYTLTGEIKYHMKLVKESRSIINTIIDQRRKSNEEHDDLLDMLLSATYED
GTTMTNEQLIDEILILFVAGHETTANALTFTLKLLAQNKDALTKVETEIKETNTKGLTPL
QELSQLNYTKCCIEESLRLYPPAWITDRVNIEDDTIDDYILKKGTIIGASIYELHRNKNY
WDDPESFKPSRFFEENRKEVLPYYMPFGAGPRLCIGNNFAMYEMILAVSAILKKFNITTD
NDTIKVNPLITLKPVDVRLKFTLKNDC

>CYP236A27(C1H87_18040)Flavivirga eckloniae

MKKSALPDPFEEARVNKGYGEMDDQNDPVTMILGLKDVRKCAHNWKTFQSGAEPGRIVIP
SEVNIRDTRQIPFEVDPPEHKDFRALVDPWFKRPLEADYSATLTQQINDIVDEMLTKDSI
EVVEGFSLRLQSRALTLLLNIPYEESETWISWGTHVFRSEGEALDGDKANILYDYIDEKI
TKAIENPGDDLYSMLLAATVNGKKLTREEVKGVMILTFAGGRDTVINAITNSIAYLAEHP
ESLNRLLNEPEITGRAVEELIRYFSPLTQMGRVVTEDTQVCEHAIKEDSRISLCWASANR
DAKVFENPNDVVLDRKINPHVAFGFSHHNCLGATHARQIMNILLKTLAKKVKSIDIIDYK
ENIEELGEFNRKVGYNSLTVKFNGR

>CYP236A33(C1A40_12590)Tamlana sp. UJ94

MKKSKIDDPFKEARETKGFGTMNDQNDPVTMILGHKDVRKCAHNWKTFQSGGDEIGRIVV
PSEVHIRDTRQIPFEVDPPQHKSYRDLVEPWFKRPLEEAYQEKLTDIINIIVDEALTKGS
MEVVSEFALKLQSRALTLLLNIPFEESELWISWGTHVFRSEDTALDGDKANVLYDYIDAQ
IDKAIANPGDDLYSKLLASEIDGKTLTKEEVKGVLILTFAGGRDTVINAITNTIAYFADN
PQALQQLRDQPEHLNSAIEELLRYYSPLTQMGRVVAEDTQVCEHAVKAQSRISMCWASAN
RDERVFENPNEVVLDRKVNPHVAFGFGTHNCLGATHARQILRILLNTLTQKVGCMQITAA
KENIEDLDEFNRKVGFDSLQVAFHKL

>CYP1099A5(C5O00_03575)Aureitalea sp. RR4-38

MAAKTKYNYPERLPLLKFFLNAEAIRKNPIPFHRQFFNLYGDTFAVKLGRRKHLMLSRDK
DVVQHILQKNHKNYYKSTIQTKYLSKYLGMGLLTANGEFWLKQRRLIQPAFHKKKMDSLL
GLMQKTIAGELDELTTESSIDVFKVMNQLAFNVVAKSLFNLSVSTDQLKRLQYIIQEIQL
FLVKEVRLPHKGWWFKLSGQVKKHKLLSEESREIIGKIIEERKASTQSHDDLLDMLLATR
YEDTREPMSTRQLVDEISILFVAGHETTANALAFTSFLLARHPKVQQRVFDEVLVAVSAT
QDPLEQIKQLPYTRAVIDESMRLYPPAWITDRENLEDDTIGDYHIRKNTLVGVSFYELHR
NPKYWENPDDFVPERFLGEHKKETAGIYYPFGAGPRMCIGMGFAIYEMVLAVSHLVYHYK
LNTQLNEPKVNPLITLKPVDILINFERRDHS

>CYP1103C13(HME9304_02119)Flagellimonas sp. HME9304

MKNLPHVSAFKVLLNSKRILRNPLPFHYENFEKLGDTFRISIPGEGEVLFSRDPELIKQV
LQKKHRYYSKSKLQTKDLAKYIGYGLLTSEGEHWRTHRRMVQPAFHVKKLKGLFGIMRNA
IVDELKRIEPNSQQNVFALMGDLAFQVVAKSLFSSNDIREPMSRLQQITEENQKMLIREM
RQPYFKWWFKASGEIKKHLKMSQIGRDILNDLIEERLTSGQEGQDLLDMLLKATYEDGSR
MPRRQLIDEVLILFTAGHETTANALAFTLYFISKDEELQAKLFAEINTLENENYTLEDLG
KLSLTMSCIKEAMRLYPPVYFIDRVATHENEINGLLMKKGTLVLLSIFELHRHPEFWNKA
TEYIPDRFMKMNMKEASNYYYPFGAGPRMCVGNAFANYEMVMVIMEIIKKFKISTKMTSV
EINPMISLKPKEVTLSFVAR

>CYP236A30(D1818_05265)Aquimarina sp. BL5

MKKSEFLDPFEKARLEKGIGEMDDQDDHVVMALRHKDVRRCAHNWKMFQSGAEPGRIVIP
SEVNIRDIRQIPFEVDPPMHGDYRTLVEAWFKRPLEENYQIELKKIITTIVEESLKKDTV
EVIREFALVLQSRALTLLFNVPFSESETWISWGTHVFRSEETALDADKANVLYKYIDVQI
ENSIKKPGEDFYSKLLESEVNGRKLTKEEIKGVIILTFAGGRDTVINIVSNSIAYFSEHP
KALEKLRSDPASINFAIEELIRYFSPLTQMGRVVTEDAQVCEHAVKADTRISLNWASANR
DESVFENANTVVLDRKINPHVAFGFGVHNCLGATHARQIMKVLIHTLIGKVQSIDLLDSK
ENIENLGEFKRKVGFDSLKVNFNSK

>CYP1103C10(D1818_11500)Aquimarina sp. BL5

MVIPKVSFFQVLKNAKNILSNPLPFHNKNFEKHGDVFEVNLGFGNSVIFTRDAGFAKHML
QNQHRKYHKSPLQSKELARYIGNGLLTSNGDHWLRQRRLIQPAFYKKKIDVIANTIRETI
CEELSRIEPNVSADIYPLMNDLAFKVVAKSLFSYTDTGNTMARLQHITETAQKSLIREIR
QPYKRWWFHLSGQIKSTLKLTQEARDILDNIIEERRKSKDTYDDLLDMLLNSKYEDGSSM
DNERLIDEILILFVAGHETTSNALSFCLSLLALHPEIQNKVFKEASEFDSEELSLMEQFQ
KSKYTTQCVEEAMRLYPPAYFSDRVNIENDEYQDIKLRKGTTVLISFFEIHRHESFWENP
TKFDPDRFHSDNKKEYSNWYFPFGAGPRMCVGSNFAMYEMIYTVSELIKKYKISTSISEI
EIKPLITLKPVNAILKFEKR

>CYP236A25(D1815_13705)Aquimarina sp. AD1

MKNSKFSDPFDKARQEKGYGEMNDQDDPVTMVLRLKDVRKCAHNWKTYQSGAVPGRIVIP
SEVNIRDTRQIPFEVDPPMHKTYRDLIEDWFKRPLLPEYQAELANIIEDIVDEAVTKDGS
VEVVEEFALCLQSRALTLLLNIPHEESKTWISWGTHVFRSEDSALDGSKANILYDYIDAQ
IEKAIRNPGNDLYSILLNSEIDNRKLTKEEVKGVMILTFAGGRDTVINSISNTIAYFAEH
KESLQLLKDNPEIISKTVEELIRYFAPLTQMGRVATQDAAVCEHAIKKYTRISLCWASAN
RDETVFENPNEVVLDRKINPHVSFGFSHHKCLGATHARQIMKILLQALTKKVTSIEIVDA
QENIEELGEFKRKVGFNSLSVKFNK

>CYP1103C9(D1815_19415)Aquimarina sp. AD1

MPVPKVSFYKVLKNAKNILSNPLPFHNKNFEKHGDIFEVNLGFGNSVIFTRDAGFAKHML
QNQHRKYYKSPLQTKDLGKYIGNGLLTSNGDHWLRQRRLIQPAFYKKKIDVIAKTIRETI
CEELSRIEPNVSMDIYPLMNDLAFKVVAKSLFSYTDTGNTMARLQHITEAAQKSLIREIR
QPYKRWWFYLSGQIKSTLTLTQEARDILNTIIEERRRSEDTYDDLLDMLLSSKYEDGSSM
DNERLIDEILILFVAGHETTSNALSFCLSLLALHPEIQESAYTEIAKYDGQELSLMEQFE
KSKYVAQCIEESMRLYPPAYFSDRVNIEKDEFNDIELSKGTTVLISFFEIHRHKDFWENP
TVFDPDRFHPDNKKKYSDWYFPFGAGPRMCVGSNFAMYEMIYAVSELIRKYKISTSISEI
EIKPLITLKPVNAILKFTPRNK

>CYP236A28(D1816_09135)Aquimarina sp. AD10

MEKSKLLDPFEEARLSKGYGKMNDQDDPVTMILRLKDVRKCAHNWKAFQSGATPGRIVVP
SEVNIRDTRQIPFEVDPPLHKDYRSLVEPWFKRPLEKEYKQKLTKQIEAIVDEVIAKKTV
EVVQDFSLRLQSQALTLLLNIPFEESETWIGWGTHVFRSEGESLDADKANLLYDYIDQQI
IKAERNPGNDLYSMLLSSEVNGKKLTHEEVKGVMILTFAGGRDTVINAVTNSIAYFAEHP
ESLKQLRENPDVTGKAVEELIRYFSPLTHMGRVATQDTQVCEHAIKADSRISLCWASANR
DASVFENPNDIVLDRKVNPHVSFGFGTHNCLGATHARQIMNILLVSLAKKVKSIEILDHT
ENIEDLEEFKRKVGYDSIKVNFNAL

>CYP1103C11(D1816_12900)Aquimarina sp. AD10

MSKTIPEVSFLRVLRNAKRILKNPLPFHNENFEKHGDVFEVNLGFGKSAIFTRDAGFAKH
MLQHQHRSYHKSSLQTKDLGRYIGNGLLTSNGEYWLKQRRLIQPAFYKKKLDLIAGTIKE
TIKEELSKITPNQTVDIHPFMSDLAFKVVAKSLFSYTDTGNTIARLQYITETAQNALIKE
IRQPYKRWWFYLSGQMKSSKMLTNEARDILNTIIEQRRNDTATYDDLLDMLLASKYDDGT
SMNNEQLIDEILILFVAGHETTSNALTFSLLLLGMHPEIQEKAYDEVMKSKDEDLSIMDQ
FAKSQYTKQCIEEAMRLYPPVYFSDRVAIESDTYKDLQLPKGTTVLISFFEIHRHLDFWK
EPLVFNPDRFNPENKKEYADWYFPFGAGPRMCVGSNFAMYEMIVVVSELIQQYTITTTSE
EIQVNPLITLKPQGSTLKFTQR

>CYP1103C6(D1013_03865)Euzebyella marina

MKKSDDLVTISQVEVFKNRKRILKNPLPFHQENFEKFGDTFKVNIGIGGRVVFTRDAETI
KYILQKNHKNYYKSSLQTKDLAKYIGNGLLTSNGDFWRAHRRMVQPAFHKRKLQGLLSIM

LSSIEQELERLNQADTIDIFPIMGDIAFQVVAQSLFSADNLRKRMRKLQHITETNQEMLI
KEMRQPYLKWWFQLSGKTNRHLAKAEEARNLLNEIIEERVDLAEEKDDLLDMLLKARYED
GSPMSRRQLIDEVLILFTAGHETTANALSFTLFLLAKHPQWQEDIFDEIKELNFSEDIMS
AIGQAQLVKNCIEEAMRLYPPVYVIDRVSLGDDTIKNRSFEKGTVWLMSMYELHRSKKFW
REPEAFDPTRFNELNSKDYSDFYYPFGAGPRMCVGNNFAMFEMIMVISTILKDYRVSAAS
EKVEINPLISLKPQNVFLKLEKR

>CYP1103C8(EQY75_01290)Muriicola sp. MMS17-SY002

MRQLTTVSQWEVIRNRKRILANPLPFHSENFQKYGDTFRVKVGPHQTIVFTRDPEIVQQV
LQKQQKKFRKSTLQTKDLAKYIGQGLLTAEGDHWKTQRRMIQPAFHKKKLVGLISTMHEA
IKEELGRIRLNEEQDIFSLMGDLAFQVVAKSLFSKSDIREKMRELQHITETNQKMLIREM
RQPYLVWWFKLSGQLRKHLNYSEDSRVLLNELIEDRVRRGGEEDDLLDMLLNATYEDGTH
MERKQLIDEVLILFTAGHETTANALSFALFLLAKHPEIQEQVYEEVSSNEITHDDYLSWL
SKFNLTQQCIEEAMRLYPPVYVIDRMAKEDIEISGYRFKKGSMLLMSVYELHRYKAFWES
PESFQPERFEKEKKKEMGPYYYPFGAGPRMCIGNNFAMYEMVITISEIVKKYKISTPYPE
VEVVPLISLKPKAVSIRFEKRQ

>CYP1252A6(Oweho_1887)Owenweeksia hongkongensis

MDTSQRNINDLPSPKGQPIVGHLLQFNSEAKHTVLENWVKEVGDVFRINMLGKPFLVSAN
PDINLEILKSRPDKFQRFFKINEILTEMGVVGVFNAEGEQWKQHRELTAEALNVKNVRSF
FSTLQMMTERLYGRWCAFAKAKKEIDVQKEMVRYTVDITTSIAFGYDVNTLEKEGDVIQD
HMEKIFPMINSRITAPIPIWRLIKSKKDKELDVALKAIEDLVHQFIEEAKARLHDNSELQ
ENPTNFLEALLVEQKKNPDFTDQEVFGNVFTILLAGEDTTSNSISWTLFYLAQHPEVYQK
VREEAQQVFGSTRCATSHEQVAELKYTEAVCMEAMRLKPVTPNLYMQAKEDVVIQDLAIP
KGTTIMMQNKVGQTDEAHFTDADQFIPERWIANGCPHHTAHSPQMMRAFGAGPRFCPGRN
LAIQEMKMAISMICKNFDLELAVKPEEVKEIFTFTMFPEGLVVKLGGV
