# Supplementary material for: Contrasting Health Effects of Bacteroidetes and Firmicutes Lies in Their Genomes: Analysis of P450s, Ferredoxins, and Secondary Metabolite Clusters
Source: Int J Mol Sci. 2022 May 2;23(9):5057. doi: 10.3390/ijms23095057 (PMC9100364; doi:10.3390/ijms23095057)
Supplement: Supplementary file 1 [file ijms-23-05057-s001.zip › Supplementary Dataset S2.pdf]

Article

Contrasting Health Effects of *Bacteroidetes* and *Firmicutes* Lies in Their Genomes: Analysis of P450s, Ferredoxins, and Secondary Metabolite Clusters

Bridget Valeria Zinhle Nkosi [1], Tiara Padayachee [1], Dominik Gront [2], David R. Nelson [3,*] and Khajamohiddin Syed [1,*]

[1] Department of Biochemistry and Microbiology, Faculty of Science and Agriculture, University of Zululand, KwaDlangezwa 3886, South Africa; brilenhle@gmail.com (B.V.Z.N.); teez07padayachee@gmail.com (T.P.)
[2] Biological and Chemical Research Center, Faculty of Chemistry, University of Warsaw, Pasteura 1, 02-093 Warsaw, Poland; dgront@gmail.com
[3] Department of Microbiology, Immunology and Biochemistry, University of Tennessee Health Science Center, Memphis, TN 38163, USA; drnelson1@gmail.com
* Correspondence: drnelson1@gmail.com (D.R.N.); khajamohiddinsyed@gmail.com (K.S.); Tel.: +1901-448-8303 (D.R.N.); +27-035-902-6857 (K.S.)

Supplementary Dataset S2: Ferredoxin sequences identified and annotated in *Bacteroidetes* species are presented according to their cluster type and subtype.

2Fe-2S
Subtype 1

>2Fe-2SST1(2506619982)Niastella koreensis GR20-10, DSM 17620

MTEREKNITLFIEYNGNHQTVETFAGEYRNLMVLIKDQVWVDGMGECGGQARCGTCMVELLNAGENLVVATDTKEAAVIRRMMTTQS
IIRLSCQILVDETLNGLKIRVMEP

>2Fe-2SST1(D1815_13710)Aquimarina sp. AD1
MPKITFITSENKTISVEGTLGSVMQLAVDNGIKGIDGDCGGVCSCATCHVFVHPDHFEKT
GAPREIEKDMLELDDNVTEYSRLCCQLEISDSLDGLIVTVAQ

>2Fe-2SST1(EJ994_03090)Maribacter sp. MJ134
MAKITFITKENQEITAEGTSGSVMELAVNNSVKGIDGDCGGVCSCATCHVHVRSEDMKVV
GAAGEIEADMLELDENVTEHSRLCCQIEITDALDGIVLEVAN

>2Fe-2SST1(D1816_09130)Aquimarina sp. AD10
MAKITFITTENKSIILEGTTGSLMQLAVDNSIPGIDGDCGGVCSCATCHVHVHPEHVEKT
GEASEIETDMLELNDHYNEYSRLCCQIEINEKLNGVVLQVAESE

>2Fe-2SST1(C1H87_18045)Flavivirga eckloniae
MAKITFITTDNKNITVEGTSGSVMQLAVDNSVPGIDGDCGGVCSCATCHVHVAPEHVEKT
GTASEIEEDMLDLNDYSNEYSRLCCQLEISDKLDGLVLQVAESD

>2Fe-2SST1(MY04_2084)Flammeovirga sp. MY04
MAKITFITKDKEEITVEGTSGSVMELAVKNNVKGIDGDCGGVCSCATCHVQVDAGHTDKT
GVASEIESDMLELDDDANEYSRLCCQLQVTEAMDGVVLNVVK

>2Fe-2SST1(Celly_2486)Cellulophaga lytica DSM 7489
MAKITFITSSNEKITVAATEGSVMELAVNNNVQGIDGDCGGVCSCATCHVFVAPEDVDKV
GVASEIETDMLELADDANEYSRLCCQIPVTDAIDNVVFTVAK

>2Fe-2SST1(ZOBELLIA_4676)Zobellia galactanivorans
MAKITFITSDDETITLEGTSGSVMALAVENGVPGIDGDCGGVCSCATCHVHVTPEDMVKT
GSASEIETDMLELDDNADEYSRLCCQIEISDAIDGVVLKVAK

>2Fe-2SST1(2620658538)Zobellia galactanivorans DsijT

MAKITFITSDDETITLEGTSGSVMALAVENGVPGIDGDCGGVCSCATCHVHVTPEDMVKTGSASEIETDMLELDDNADEYSRLCCQI
EISDAIDGVVLKVAK

>2Fe-2SST1(2758560153)Tenacibaculum jejuense KCTC 22618

MANITFITSDNEKIILEGDSGNVMQLAVNNSVNGIDGDCGGVCSCATCHVFVAEKDIDKVGKASSIEVDMLELADDANEFSRLCCQI
EITEAIDGVELTVAK

>2Fe-2SST1(TJEJU_2940)Tenacibaculum jejuense
MANITFITSDNEKIILEGDSGNVMQLAVNNSVNGIDGDCGGVCSCATCHVFVAEKDIDKV
GKASSIEVDMLELADDANEFSRLCCQIEITEAIDGVELTVAK

>2Fe-2SST1(2718723938)Wenyingzhuangia fucanilytica CZ1127

MAKITFITSDNEKVTLEGTSGNVMQLAVNNGVSGIDGDCGGVCSCATCHVFVAASDMEKVGVASEIETDMLELADDANEFSRLCCQI
EISEAIDGVELTVAK

>2Fe-2SST1(Celal_3019)Cellulophaga algicola
MAQITFISTDNKSTVVSGNSGNLMELALQNKIEGIEGNCGGVCSCATCHIYVQKEDWTKI
GPPNEMESDMLEFDDKTTNYSRLSCQIQVTDAIDGIVVNVAK

>2Fe-2SST1(649896958)Cellulophaga algicola IC166, DSM 14237

MAQITFISTDNKSTVVSGNSGNLMELALQNKIEGIEGNCGGVCSCATCHIYVQKEDWTKIGPPNEMESDMLEFDDKTTNYSRLSCQI
QVTDAIDGIVVNVAK

>2Fe-2SST1(C1A40_12585)Tamlana carrageenivorans
MKTITFITSDNDIIETQANFGSLMELAVKNKVKGIDGDCGGVCSCATCHVHVNQEFWNAV
GGPSELESDMLEFDDNVSDYSRLSCQLKVSQVPDGLILKVAK

>2Fe-2SST1(D1818_05260)Aquimarina sp. BL5
MAKITFVTSDDIKTTVEATTGNIMELAVQNKIKGIDGNCGGVCSCATCHVHVQPEDWSKI
GEPSELEKDMLEFDENVSEYSRLSCQIKVSDAIDGIVLKVAN

>2Fe-2SST1(DJ013_00495)Arcticibacterium luteifluviistationis
MAKITFITQNEEEITLEADSGSVMELAVENGVEGIDGDCGGVCSCATCHVHVLPEFVSKT
GEASEIEKDMLELDDKVNEYSRLSCQMEISEDLDGIILKVAN

>2Fe-2SST1(BW723_04385)Polaribacter reichenbachii
MAKITFITSDNETITLEGTSGSVMELAVNNNVKGIDGDCGGVCSCATCHVHVAPEDFAKT
GGPEELENDMLEFDDLTNEYSRLSCQLQISEALDGIVLKVAK

>2Fe-2SST1(2758621181)Polaribacter sp. SA4-12

MAKITFITSDNESVTVEGTSGSVMELAVNNNIKGIDGDCGGVCSCATCHVYVAPDSVAKTGEASELENDMLEFDDLTNEYSRLSCQL
QVSEDLDGVIFKVAK

Subtype 2
>2Fe-2SST2(Emtol_3252)Emticicia oligotrophica
MPVITYIENNGNQRQVELPMGASIMEGAIQNDVKGIVAECGGSCMCATCHVYVDEQFIDL
LPEMQEEEDEMLEAANAPRQANSRLGCQVRVTKAMDGLVVRIPERQ

>2Fe-2SST2(2506488740)Emticicia oligotrophica GPTSA100-15, DSM 17448

MPVITYIENNGNQRQVELPMGASIMEGAIQNDVKGIVAECGGSCMCATCHVYVDEQFIDLLPEMQEEEDEMLEAANAPRQANSRLGC
QVRVTKAMDGLVVRIPERQ

>2Fe-2SST2(Runsl_0439)Runella slithyformis
MPNITYIEPNGTAKTFDLPMGATLMEGAVQNGVHGIVAECGGSCMCATCHIYVDEAFVDI

LPEMEEEEDEMLEGATAERQPNSRLGCQVRATQKLDGLIVRIPEIQ

>2Fe-2SST2(2505789681)Runella slithyformis LSU4, DSM 19594

MPNITYIEPNGTAKTFDLPMGATLMEGAVQNGVHGIVAECGGSCMCATCHIYVDEAFVDILPEMEEEEDEMLEGATAERQPNSRLGC
QVRATQKLDGLIVRIPEIQ

>2Fe-2SST2(DR864_05020)Runella sp. HYN0085

MPKVIYIENNGTEHQVDLPLGATLMEGAVQNDIKGIVAECGGSCMCATCHIYVDEQFMGL
LPEMEEEENEMLEGATAERMSNSRLGCQVRISNKLDGVIVRIPEKQ

Subtype 4
>2Fe-2SST4(644928285)Dyadobacter fermentans NS114, DSM 18053

MINIFIENDLGERQALEVPTDMGFNLMELLKAYEYDIQATCGGMALCATCHIEVLEGKENLPESNDQELDTLDTLPNADANSRLSCQ
LRPSPAMDGLVFRLKALQEA

>2Fe-2SST4(Dfer_0408)Dyadobacter fermentans
MINIFIENDLGERQALEVPTDMGFNLMELLKAYEYDIQATCGGMALCATCHIEVLEGKEN
LPESNDQELDTLDTLPNADANSRLSCQLRPSPAMDGLVFRLKALQEA

>2Fe-2SST4(2598915479)Sphingobacterium sp. ML3W

MENIIEIEIEDRDGSTQKIEVPTDVNLSLMELLKATNYEVLATCGGIALCATCHVQIKSGAENLSEPQEQELDMLDTLPDADDDSRL
ACQLWLKNENDGLRIKIKGALQ

>2Fe-2SST4(Sph21_2968)Sphingobacterium sp. 21
MIHITVEDRTGSVETIEIPEDINLSLMEVLKASEYNILATCGGMALCATCHVEVLSGMER
LHEASDDELNMLDTLPDADENSRLACQIRLNEQLDGLHIKIKGSLD

>2Fe-2SST4(D3H65_05005)Paraflavitalea soli
MINITVLNKAGEERLLEVPEDMGLNLMEILKANEYNVLATCGGMAMCGTCHVQVLEGMDN
LGAHSDAELDMLDTLPDAESNSRLSCQLQINENMDGAVFKLMAEEDIAV

>2Fe-2SST4(CWM47_06365)Spirosoma pollinicola
MISFTVEDRNGERQPIEIPEGINLSLMEVLKASDYTILATCGGLAICATCHVQVLNGLDA
LPEPQDAELDMLDTLPDADSDSRLSCQIRIDETVDGAIFRIKSEEPI

>2Fe-2SST4(646496845)Spirosoma linguale DSM 74

MINFTIEDRNGERQDLEIPEGINLSLMEVLKASDYKILATCGGMALCATCHVQVLNGFDDLPAAQDAELDMLDTLPDADFDSRLACQ
IRVNEAIEGAVFRIKSDEPD

>2Fe-2SST4(Slin_3269)Spirosoma linguale
MINFTIEDRNGERQDLEIPEGINLSLMEVLKASDYKILATCGGMALCATCHVQVLNGFDD
LPAAQDAELDMLDTLPDADFDSRLACQIRVNEAIEGAVFRIKSDEPD

>2Fe-2SST4(SD10_17460)Spirosoma radiotolerans
MITFTVEDRDGEQKPVEVPEGISLSLMEVLKASDYNILATCGGMALCATCHVQVVNGFDN
LPPAQDLELDMLDTLPDADSDSRLACQIRVDEAVEGALFRIKSEEPS

>2Fe-2SST4(2600232563)Spirosoma radiotolerans DG5A (Spirosoma radiotolerans genome sequence)

MITFTVEDRDGEQKPVEVPEGISLSLMEVLKASDYNILATCGGMALCATCHVQVVNGFDNLPPAQDLELDMLDTLPDADSDSRLACQ
IRVDEAVEGALFRIKSEEPS

>2Fe-2SST4(FLA_4789)Filimonas lacunae
MMINITVENRDGERRNLEIPDDMNLSLMEALKAYEYNILATCGGMALCATCHVQVLQGLP
GLPPMKDAEMDMLDTLPDAASDSRLACQLRVDETMEGAIFKIMGEEE

>2Fe-2SST4(Runsl_3439)Runella slithyformis
MASMRFKDFIEHPPMIQFTIEDRGGERQLLEIPEGIGLNLMEVLKASDYNILATCGGMAL
CATCHVEVLEGGDSLPSVSDAELDILDTLPAATSCSRLACQLRVDEAMEGTTFKIRGEEH

>2Fe-2SST4(2505792702)Runella slithyformis LSU4, DSM 19594

MASMRFKDFIEHPPMIQFTIEDRGGERQLLEIPEGIGLNLMEVLKASDYNILATCGGMALCATCHVEVLEGGDSLPSVSDAELDILD
TLPAATSCSRLACQLRVDEAMEGTTFKIRGEEH

>2Fe-2SST4(DR864_08635)Runella sp. HYN0085
MIQFTIEDGIGERQTLEIPEGIGLNLMEVLKASEYNILATCGGMALCATCHVEILDGGDS
LAPVSDAELDILDTLPSATSCSRLACQLRVDETMVGTTFKIRGEEH

>2Fe-2SST4(DTQ70_17720)Runella sp. SP2
MIQFTIEDRTGERQALEIPEGIGLNLMEVLKASEYSILATCGGMALCATCHVGVLDGGEN
LPSVSDAELDILDTLPSATSCSRLACQLRVDETLQGTTFKILGEEE

>2Fe-2SST4(ZPR_0508)Zunongwangia profunda
MSDIKITIIDREGEAHTVDAPTDMNMNLMEVIRSYELAPEGTIGICGGMAMCASCQCYVL
NLEHMLPEQSFEEEDMLDQAFFVEDNSRLSCQIPITEDLDGLEVKLAPEST

>2Fe-2SST4(646744637)Zunongwangia profunda SM-A87

MSDIKITIIDREGEAHTVDAPTDMNMNLMEVIRSYELAPEGTIGICGGMAMCASCQCYVLNLEHMLPEQSFEEEDMLDQAFFVEDNS
RLSCQIPITEDLDGLEVKLAPEST

>2Fe-2SST4(AO058_02530)Salegentibacter sp. T436
MSDIKITIIDREGEAHVVDAPTDMNMNLMEVVRSYELAAEGTIGICGGMAMCASCQCYIL
NFEHMLPEQSIEEEDMLDQAFFVEDNSRLSCQIPITEELDGLEVKLAPEAP

>2Fe-2SST4(C7S20_08180)Gramella fulva
MSDVKITIIDREGEAHTIDAPTDMNMNLMEVIRSYELAPEGTIGICGGMAMCASCQCYML
NLEHMLPEKSIEEEDMLDQAFFVEDNSRLSCQIPITHELDGLEIRIAPVSEE

>2Fe-2SST4(GFO_0126)Gramella forsetii
MSDVKITIIDREGEAHTIDAPTDMNMNLMEVIRSYELAPEGTIGICGGMAMCASCQCYML
NFEHMLPEMSIEEEDMLDQAFLVEDNSRLSCQIPITEGLDGLEVKIAPSSE

>2Fe-2SST4(639716986)Gramella forsetii KT0803

MSDVKITIIDREGEAHTIDAPTDMNMNLMEVIRSYELAPEGTIGICGGMAMCASCQCYMLNFEHMLPEMSIEEEDMLDQAFLVEDNS
RLSCQIPITEGLDGLEVKIAPSSE

>2Fe-2SST4(GRFL_1897)Gramella flava
MSDIKITIIDRQGEAHTIDAPTDMNMNLMEVIRSYELAEEGTIGICGGMAMCASCQCYIL
NFEHMLPEKSIEEEDMLDQAFFVEDNSRLSCQIPITEDLEGLEIRIAPALE

>2Fe-2SST4(LPB144_08575)Gramella salexigens
MADVKITIIDREGEAHTIDAPTDMNMNLMEVIRSYELAPEGTIGICGGMAMCASCQCYIH
NFEHMLPEKSYEEEDMLDQAFFVEDNSRLSCQIPITEDLEGLEVKIAPASE

>2Fe-2SST4(2719632093)Gramella sp. LPB0144

MADVKITIIDREGEAHTIDAPTDMNMNLMEVIRSYELAPEGTIGICGGMAMCASCQCYIHNFEHMLPEKSYEEEDMLDQAFFVEDNS
RLSCQIPITEDLEGLEVKIAPASE

>2Fe-2SST4(FLA_1756)Filimonas lacunae
MYQIKVNFEQKGLAPVVLENIASNQSLLEICLDNGIELHHNCGAVCACSTCHVYIDKGEE
FIPEITDREEDFIDRAVNPRIHSRLGCQCVLKPGSGSLEITLPDQTQFLGE

>2Fe-2SST4(AQ505_05245)Pedobacter sp. PACM 27299
MSIFKLKINFEEKGKEPIELPIAGGESVLDVCHDHGIELQHNCGGVCGCSTCHVYVTKGM
DNIQEISDKEEDFIDRAVRPRITSRLGCQCVVISGDIEVTIPDQSEFLGH

>2Fe-2SST4(644936868)Pedobacter heparinus HIM 762-3, DSM 2366

MSIFKLKINFEEQGKETIELPIAGGESVLDVCLDHGIELQHNCGGVCGCSTCHVYVTRGMDDIQEISDKEEDFIDRAVRPKISSRLG
CQCVVISGDIEVTIPDQSEFLGH

>2Fe-2SST4(AY601_0102)Pedobacter cryoconitis
MNIYQLTVNFEEKGKAQIQLPIAGGESVLEVCLDNGIDLQHNCGGVCGCSTCHVYVTKGM
DNIQEISDKEEDFIDRAVSPKISSRLGCQCIVINGNIEVTIPNQSEFLGH

>2Fe-2SST4(CA264_18685)Pontibacter actiniarum
MTFKFADGSPDETHPAVEGESVLDVALNNDIKLQHNCGGVCGCSTCHVYVEAGMDDLPEI
SDKEEDYIDRAVDPRINSRLGCQCVVQGNEDIVVTIPEQDFLGH

>2Fe-2SST4(PKOR_07765)Pontibacter korlensis
MKVVNITFKFADGSPDETHPAVEGESVLDVALNNNIKLQHNCGGVCGCSTCHVYIEAGMD
DLPEISDKEEDYIDRAIDPRINSRLGCQCVVQGNEDVVVTIPEQDFLGH

>2Fe-2SST4(2629378787)Pontibacter korlensis X14-1T

MKVVNITFKFADGSPDETHPAVEGESVLDVALNNNIKLQHNCGGVCGCSTCHVYIEAGMDDLPEISDKEEDYIDRAIDPRINSRLGC
QCVVQGNEDVVVTIPEQDFLGH

>2Fe-2SST4(N008_05540)Hymenobacter sp. APR13
MKAVNITFQFQDGQPEQTHVAAEGESVLDVALNNGIQLQHNCGGVCGCSTCHVYVQRGEA
DLPEISDKEEDFIDRAVNPRINSRLGCQCVVQASSQDLVILIPAQEFLGH

>2Fe-2SST4(D3Y59_10430)Hymenobacter oligotrophus
MKAVNITFKFSDGQPEQTHVAAEGESVLDVALNNGIQLQHNCGGVCGCSTCHVYVLQGGD
ELPEISDKEEDFIDRAVNPRLNSRLACQCVVQTTSENLVIEVPPQDFLGH

>2Fe-2SST4(PK28_04285)Hymenobacter sp. DG25B
MKAVNITFKFEDGQPEQTHVAAEGESVLDVALNNGIQLQHNCGGVCGCSTCHVYVVQGEN
DLPEISDKEEDFIDRAANPRINSRLGCQCVVQGHEDLVILVPPQDFLGH

>2Fe-2SST4(AM218_11920)Hymenobacter sp. DG25A
MTFKFEDGQPEQTHVAAEGESVLDVALNNGIQLQHNCGGVCGCSTCHVYVLQGEADLPEI
TDKEEDFIDRAANPRINSRLGCQCVVQGHEDLVILVPPQDFLGH

>2Fe-2SST4(A0257_17220)Hymenobacter sp. PAMC 26554

MIFQFQDGQPAQTHVAASGESVLDVALNNGIQLQHNCGGVCGCSTCHVYIDQGGDELPEI
SEKEEDFIDRAINPRISSRLACQCVLPNEALKVVVTLPPQHFLGH

>2Fe-2SST4(AUC43_01490)Hymenobacter sedentarius
MTFQLSDGQPAQTHVAAPGESVLDVALNNGIQLQHNCGGVCGCSTCHVYINSGGDDLPEI
SDKEEDFIDRAENPRINSRLACQCVVQAGTQLVVTIPPQHFLGH

>2Fe-2SST4(DDQ68_19025)Hymenobacter nivis
MGPLPNPGPGRAVEPQRLAGFPAAFSFTKDPKFVPATTITFQFKDGQPAQTHVAAAGESV
LDVALNNAIQLQHNCGGVCGCSTCHVYVDRGGDDLPEISDKEEDFIDRAENPRINSRLAC
QCVVEAGMELTVTVPPQHFLGH

>2Fe-2SST4(AXW84_03155)Hymenobacter sp. PAMC 26628
MTFQFKDGQPAQTHVAAAGESVLDVALNNAIQLQHNCGGVCGCSTCHVYVDRGGDDLPEI
SDKEEDFIDRAENPRINSRLACQCVVNAGMELTVTIPHQHFLGH

>2Fe-2SST4(SGRA_1112)Saprospira grandis
MAIVKFTFEDPNIPPKELEASLGDNISELADDNDIHINHNCGRVCACSTCHVYIEEGEDS
LPEISDREEDFIDRALDPRIESRLACQCIIQEDDAVIEVLVPDQTRIIGHEH

>2Fe-2SST4(2504769607)Haliscomenobacter hydrossis O, DSM 1100

MATVKFTFEDKKITQPIVVEGVTEGTSILDVTEDYDIHLNHNCGGVCACSTCHVYVHRGEDDLEEISDKEEDFIDRAHNPRLNSRLG
CQCIILDGEAYIEIEIPDQSRIIGHEH

>2Fe-2SST4(DJ013_11770)Arcticibacterium luteifluviistationis

MDKISITVLRNGLERKIETKPNEYKNLMFLLKDHCYPDDFGECGGMGRCATCIIKASGLKGAALEKDRNEPATLEKFRQSDPNLRLA
CGLHISSDLEGAIIELIEI

>2Fe-2SST4(AY601_4392)Pedobacter cryoconitis
MEENNITVHVQNPDGTLTSLEAPVDMGLSLMEYLKACEYDILATCGGMALCATCCVDVLE
GEDKLKEMTDDEYAMLDTLPDLLPNSRLACQLQLSHEMDGLVVKLHGTD

>2Fe-2SST4(AQ505_01285)Pedobacter sp. PACM 27299
MEENNITLHVQNPDGSRTTLEAPVDMGLSLMEYLKACEYDILATCGGMALCATCCVDVLE
GEEKLNEMTDDEYAMLDTLPDLLPNSRLACQLQLSPAMDGLVVKLH

>2Fe-2SST4(644937785)Pedobacter heparinus HIM 762-3, DSM 2366

MEENNITLHVQNPDGSRTTLEAPVDMGLSLMEYLKACEYDILATCGGMALCATCCVDVLEGEDKLNEMSDDEYAMLDTLPDVLPNSR
LACQLQLNPAMDGLVVKLHHAE

Subtype 18
>2Fe-2SST18(EAG08_15815)Chryseobacterium sp. 3008163
MNDINIRITDREGLTHDIVAPTDMSMNLMEIIRSYELAEEGTIGVCGGMAMCASCQVYVI
KDPGLEPMGDEEDAMLGEAFHVEPNSRLGCQLHMAMEMEGLEVQIAPYP

>2Fe-2SST18(EQY75_11450)Muriicola sp. MMS17-SY002
MSDIQVTITDRAGKTHKVSAPTDMNMNLMELLRSYELAPEGTIGICGGMAMCASCQVYII
SSHEMEAKSDEEEAMLSEAFYVKDNSRLSCQIPLEDSLDELVVELAPES

>2Fe-2SST18(PKOR_21535)Pontibacter korlensis
MKDAINIYVEQESGERIELEAPLDMNLSVMEVLKANEFPVQAVCGGMAICATCHVEVLES
GPLPEMNDDEAYMLETLPHATDSSRLSCQLRVNPELDGLVVRIMPEA

>2Fe-2SST18(2629376846)Pontibacter korlensis X14-1T

MKDAINIYVEQESGERIELEAPLDMNLSVMEVLKANEFPVQAVCGGMAICATCHVEVLESGPLPEMNDDEAYMLETLPHATDSSRLS
CQLRVNPELDGLVVRIMPEA

>2Fe-2SST18(CA264_09220)Pontibacter actiniarum
MKDAINIYVEQDGGERIELEAPLDMNLSVMEVLKANEFPVQAVCGGMAICATCHVEVLQS
GELPDMSDDEAYMLETLPHATDTSRLSCQLRVTPELDGLVVRIMPEA

>2Fe-2SST18(D770_13065)Flammeovirgaceae bacterium 311
MENMINLFVQQENGERLKLEAPTDMGLSVMEVLKAHELEVQAMCGGMAICATCHVEVLES
GTLPDQSEDEAYMLESLPHAVSGSRLSCQLRVSPELDGLVVRIMPEA

>2Fe-2SST18(D3Y59_17120)Hymenobacter oligotrophus
MSLEDITEVRVYVEDAPGQRTELVAPTDMSLSLMEVMKASGYDIQATCGGMALCGTCHVE
VLAGPELPEPGDDEMAMLESLPIMSSGSRLSCQIRITSRLDGLVVRLMPQNA

>2Fe-2SST18(PK28_14650)Hymenobacter sp. DG25B
MDVTEVRVYVEEEPGHRREVVGPTDMGLSLMELLKADGYDIQATCGGMALCGTCHVEVLA
GPELPEPGDDELAMLESLPVMTQGSRLSCQIRLNARLDGLVVRLMPQGT

>2Fe-2SST18(AM218_01780)Hymenobacter sp. DG25A
MSLDVTEVRVYVEEEPGHRREIVGPTDMGLSLMELLKADGYDIQATCGGMALCGTCHVEV
LAGPELPEPSDDELAMLESLPVMTQGSRLSCQIRINARLDGLVVRLMPQGT

>2Fe-2SST18(A0257_09945)Hymenobacter sp. PAMC 26554
MTDIRIYVEEAPGQRREIEGPTDMGLSLMELLKASDYPIQATCGGMALCATCHVEVLAGP
PLPEPSEDEWAMLDTLPVLHETSRLSCQIRLAPNLDGLVVRIADPA

>2Fe-2SST18(AUC43_08825)Hymenobacter sedentarius
MEEDIRIYVEEAPGQRREIIAPTDMGLSLMEVLKASGYDIMATCGGMALCATCHVEVLAG
PALFEPNDDELDMLETLPVIHPGSRLSCQIRLTPQTDGLVVRLAPTGA

>2Fe-2SST18(DDQ68_11735)Hymenobacter nivis
MENDIRIYVEEAPGQRRELEAPTDMGLSLMELLKANDYPIQAACGGMALCATCHVEVLAG
PALPEPQDAELDMLETLPHVYEGSRLSCQIRLHPHTDGLVVRIAG

>2Fe-2SST18(AXW84_16910)Hymenobacter sp. PAMC 26628
MENDIRIYVEEAPGQRRELEAPTDMGLSLMELLKANDYPIQATCGGMALCATCHVEVLAG
PVLPEPQDAELDMLETLPHVYEGSRLSCQIRLEPRNDGLVVRIAG

>2Fe-2SST18(2620656227)Zobellia galactanivorans
MSLKKLSLTYIDEFGETCQADFNNHEYHSLMELLFDKYLQDWGDCKGRAWCGTCHIQLHSDRNLEKMDINEENTLSNITGRTKTSRL
ACQIPLDSNLDGIVFSILKDDAV

>2Fe-2SST18(IX49_04190)Cellulophaga lytica HI1
MSDIKLKITDREGVLHEVDAPTDMNMNLMEVVRSYELAPEGTIGICGGMAMCASCQCYVKSDHELPEMSDDEDAMLAEAFNVEDNSR
LGCQIHMTPDLDGLEVELAPES

>2Fe-2SST18(P700755_002323)Psychroflexus torquis
MDVKITIVDREGTSHLVDAPTDMAMNLMEIIRSYELAPEGTIGICGGMAMCASCQCYIQS
ITPLPEKGDEEEAMLAEAFYVEDNSRLSCQLPITESMEGLKIELAPSEE

>2Fe-2SST18(2758371622)Psychroflexus torquis ATCC 700755

MDVKITIVDREGTSHLVDAPTDMAMNLMEIIRSYELAPEGTIGICGGMAMCASCQCYIQSITPLPEKGDEEEAMLAEAFYVEDNSRL
SCQLPITESMEGLKIELAPSEE

>2Fe-2SST18(Aeqsu_1584)Aequorivita sublithincola
MKDIKITITDRQGVKHQVDAPTDMNMNVMELVRSYELAPEGTIGICGGMAMCASCQCYIL
SDHELPEMSYDEDLMLAEAFNVKDNSRLSCQIFIKEELHGLEVELAPEV

>2Fe-2SST18(2509582627)Aequorivita sublithincola QSSC9-3, DSM 14238

MKDIKITITDRQGVKHQVDAPTDMNMNVMELVRSYELAPEGTIGICGGMAMCASCQCYILSDHELPEMSYDEDLMLAEAFNVKDNSR
LSCQIFIKEELHGLEVELAPEV

>2Fe-2SST18(DZC78_08715)Olleya aquimaris
MEQDINIKITDRDGVQHSIEAPTDMAMNLMEVVRSYELAPEGTIGICGGMAMCASCQCYV
ISDTTLPEMQDDEEAMLSEAFNVQDNSRLGCQIQITPEMEGLEVILAPEE

>2Fe-2SST18(C1H87_15020)Flavivirga eckloniae
MEDINIKIKDREGTLHDIVAPTDMAMNLMEVVRSYELAPEGTIGVCGGMAMCASCQCYVL
SQTDLPEMSDDEEAMLAEAFDVKNNSRLGCQIQITPEMQGLEVELAPES

>2Fe-2SST18(CW732_01385)Olleya sp. Bg11-27
MEQDINIKITDRDGVTHQIVAPTDMAMNLMEIVRSYELAPEGTIGICGGMAMCASCQCYV
LSDTPLPEMQDDEEAMLSEAFDVKDNSRLGCQIQMTPEMEGLEVELAPES

>2Fe-2SST18(C1A40_09385)Tamlana carrageenivorans
MEDINIKITDRDGVTHDIVAPTDMAMNLMEVVRSYELAPEGTIGVCGGMAMCASCQCYVL
SDTVLPEMSDDEEAMLSEAFDVKDNSRLGCQIQMTPEMEGLEVELAPES

>2Fe-2SST18(M667_02915)Cellulophaga baltica NN016038
MADIKIKITDREGVTHEVDAPTDMNMNVMELVRAYELAPEGTIGVCGGMAMCASCQCYVL
NDVALPEKSDDEDAMLAEAFYVKDNSRLGCQIHLSEDLEGLELELAPES

>2Fe-2SST18(M666_02895)Cellulophaga baltica 18
MADIKIKITDREGVTHEVDAPTDMNMNVMELVRAYELAPEGTIGVCGGMAMCASCQCYVL
NDVALPEKSDDEDAMLAEAFYVKDNSRLGCQIHLSEDLEGLELELAPES

>2Fe-2SST18(649894956)Cellulophaga algicola IC166, DSM 14237

MADIKIKIIDREGVAHEVDAPTDMNMNVMELVRAYELAPEGTIGVCGGMAMCASCQCYVLNDVALPEKSDDEDAMLAEAFYVKDNSR
LGCQIHLTDDLEGLELELAPES

>2Fe-2SST18(Celal_1007)Cellulophaga algicola
MADIKIKIIDREGVAHEVDAPTDMNMNVMELVRAYELAPEGTIGVCGGMAMCASCQCYVL
NDVALPEKSDDEDAMLAEAFYVKDNSRLGCQIHLTDDLEGLELELAPES

>2Fe-2SST18(BW723_08810)Polaribacter reichenbachii
MSVDINIKITDRNGETHEIVAPTDMAMNLMEVVRSYELAEEGTIGICGGMAMCASCQCYV
KSDHELPEMTDDEDAMLAEAFNVEDNSRLGCQIQMTPELEGLEVELAPES

>2Fe-2SST18(2758620850)Polaribacter sp. SA4-12

VQDINIKITDRNGETHEVVAPTDMAMNLMEVVRSYELAEEGTIGVCGGMAMCASCQCYVTSETELPEMSDDEDAMLAEAFNVEDNSR
LGCQIQMTPAMEGLEVILAPES

>2Fe-2SST18(2718723467)Wenyingzhuangia fucanilytica CZ1127

MSDITIKIKDREGVVHELQAPTDMAMNIMELCKAYELPVEGTCGGMAMCASCQCYVLSDHELPEMGDAEEDMLDQAFYVEDNSRLGC
QLPITEDLDGLEIELAPESEV

>2Fe-2SST18(2758560729)Tenacibaculum jejuense KCTC 22618

MDINIKIKDREGVIHDVVAPTDMAMNLMEVVRSYELAPEGTIGICGGMAMCASCQCYVTSNHELPEMSDDEDAMLAEAFDVEDNSRL
GCQIQMTPDLEGLVVELAPES

>2Fe-2SST18(TJEJU_3977)Tenacibaculum jejuense
MEDQDITIKITDREGVTHEVQAPTDMNMNLMEVIRSFELAPEGTVGICGGMAMCASCQCY
VKSEHELPEMSDDEDAMLAEAFYVEDNSRLGCQIHMTPELDGLEVEMAPES

>2Fe-2SST18(2758561177)Tenacibaculum jejuense KCTC 22618

MEDQDITIKITDREGVTHEVQAPTDMNMNLMEVIRSFELAPEGTVGICGGMAMCASCQCYVKSEHELPEMSDDEDAMLAEAFYVEDN
SRLGCQIHMTPELDGLEVEMAPES

>2Fe-2SST18(SB49_13885)Sediminicola sp. YIK13
MSEIKIKIKDREGVLHEVDAPTDMNMNLMEIVRSYELAPEGTIGICGGMAMCASCQCYVN
SGHELPEMSDDEDAMLSEAFYVKDNSRLGCQIHMTDDLNGLEVELAPES

>2Fe-2SST18(2723589690)Cellulophaga lytica DAU203

MSDIKLKITDREGVLHEVDAPTDMNMNLMEVVRSYELAPEGTIGICGGMAMCASCQCYVKSDHELPEMSDDEDAMLAEAFNVEDNSR
LGCQIHMTPDLDGLEVELAPES

>2Fe-2SST18(Celly_0878)Cellulophaga lytica DSM 7489
MSDIKLKITDREGVLHEVDAPTDMNMNLMEVVRSYELAPEGTIGICGGMAMCASCQCYVK
SDHELPEMSDDEDAMLAEAFNVEDNSRLGCQIHMTPDLDGLEVELAPES

>2Fe-2SST18(IX49_04190)Cellulophaga lytica HI1
MSDIKLKITDREGVLHEVDAPTDMNMNLMEVVRSYELAPEGTIGICGGMAMCASCQCYVK
SDHELPEMSDDEDAMLAEAFNVEDNSRLGCQIHMTPDLDGLEVELAPES

>2Fe-2SST18(649969953)Cellulophaga lytica LIM-21, DSM 7489

MSDIKLKITDREGVLHEVDAPTDMNMNLMEVVRSYELAPEGTIGICGGMAMCASCQCYVKSDHELPEMSDDEDAMLAEAFNVEDNSR
LGCQIHMTPDLDGLEVELAPES

>2Fe-2SST18(D1013_19300)Euzebyella marina
MSDIKIKITDREGVTHEVDAPTDMNMNLMEVVRSYELAPEGTIGVCGGMAMCASCQCYVK
SDHVLPEMSDDEDAMLAEAFNVEDNSRLGCQIHMTEDLDGLEVELAPES

>2Fe-2SST18(2620655324)Zobellia galactanivorans DsijT

MSDIKIKITDRDGVTHEVDAPTDMNMNLMEVVRSYELAPEGTIGICGGMAMCASCQCYVQSDHQLPEKSDDEDAMLAEAFNVEDNSR
LGCQIHMTPDLDGLVVELAPES

>2Fe-2SST18(ZOBELLIA_1286)Zobellia galactanivorans
MSDIKIKITDRDGVTHEVDAPTDMNMNLMEVVRSYELAPEGTIGICGGMAMCASCQCYVQ
SDHQLPEKSDDEDAMLAEAFNVEDNSRLGCQIHMTPDLDGLVVELAPES

>2Fe-2SST18(C5O00_06810)Pukyongia salina
MQDIKITIIDREGEEHLVDAPTDMNMNVMEIVRSYELAPEGTIGVCGGMAMCASCQCYVL
SNHELPKKSDEEEAMLAEAFYVEDNSRLGCQIRITPQLDGLKVQLAPES

>2Fe-2SST18(MED134_10975)Dokdonia sp. MED134
MSADIKITIIDREGVSHAIDAPTDMNMNLMEIVRSYELAPEGTIGVCGGMAMCASCQCYV
ESDHELPEMSDDEDAMLAEAFYVEDNSRLGCQLHISPEMEGLQVRLAPES

>2Fe-2SST18(EJ994_15770)Maribacter sp. MJ134
MTDIKIKITDREGVLHEIDAPTDMNMNLMEVVRSYELAPEGTIGICGGMAMCASCQCYIE
SNHELPEKSDDEEAMLAEAFNVEENSRLGCQLHITEDMAGLEVVLAPEES

>2Fe-2SST18(YQ22_06105)Maribacter sp. 1_2014MBL_MicDiv
MTDIKITIIDRDGVSHEIDAPTDMNMNLMEVVRSYELAPEGTIGICGGMAMCASCQCYVE
SDHELPEMSDDEEAMLSEAFNVKDNSRLGCQLHISEDMDGLKVELAPEDV

>2Fe-2SST18(2597962883)Dokdonia sp. PRO95

MIDIKITITDRDGVAHEIDAPTDMNMNLMEVVRSYELAPEGTIGICGGMAMCASCQCYVESDHDLPEMGDDEEAMLAEAFHVQDNSR
LGCQLHIHQDMDGLRVTLAPEE

>2Fe-2SST18(Krodi_1475)Dokdonia sp. 4H-3-7-5
MTDIKITITDRDGVAHEIDAPTDMNMNLMEVVRSYELAPEGTIGICGGMAMCASCQCYVE
SDHVLPEMSDDEEAMLAEAFHVEDNSRLGCQLHIHRDMDGLRVTLAPEE

>2Fe-2SST18(DCS32_04550)Dokdonia sp. Dokd-P16
MTDIKITITDRDGVAHEIDAPTDMNMNLMEVVRSYELAPEGTIGICGGMAMCASCQCYVE
SDHVLPEMSDDEQAMLAEAFHVEDNSRLGCQLHIHPEMDGLRVTLAPEE

>2Fe-2SST18(644740727)Flavobacterium johnsoniae UW101, ATCC 17061

MDVLIKIKDREGVIHELQAPTDMAMNIMELCKAYELPVEGTCGGMAMCASCQCYVLNDVALPEMGDEEEAMLSEAFYVKSNSRLGCQ
IPITEELEGLELELAPEY

>2Fe-2SST18(Fjoh_1324)Flavobacterium johnsoniae UW101
MDVLIKIKDREGVIHELQAPTDMAMNIMELCKAYELPVEGTCGGMAMCASCQCYVLNDVA
LPEMGDEEEAMLSEAFYVKSNSRLGCQIPITEELEGLELELAPEY

>2Fe-2SST18(AXE80_01550)Wenyingzhuangia fucanilytica
MSDITIKIKDREGVVHELQAPTDMAMNIMELCKAYELPVEGTCGGMAMCASCQCYVLSDH
ELPEMGDAEEDMLDQAFYVEDNSRLGCQLPITEDLDGLEIELAPESEV

>2Fe-2SST18(2718723467)Wenyingzhuangia fucanilytica CZ1127

MSDITIKIKDREGVVHELQAPTDMAMNIMELCKAYELPVEGTCGGMAMCASCQCYVLSDHELPEMGDAEEDMLDQAFYVEDNSRLGC
QLPITEDLDGLEIELAPESEV

>2Fe-2SST18(D1816_11805)Aquimarina sp. AD10
MSDITIKIKDREGVVHELQAPTDMAMNLMEVCKAYELPVEGTCGGMAMCASCQCYVLSDH
QLPEMGQDEDLMLAEAFYVEDNSRLGCQIPITTDLDGLEIELAPES

>2Fe-2SST18(D1818_10405)Aquimarina sp. BL5
MSDVTIKIKDREGEIHEVQAPTDMAMNLMEVCKAYELPVEGTCGGMAMCASCQCYVLSDH
KLPEMSQDEDLMLAEAFYVEDNSRLGCQIPITPELDGLEIELAPES

>2Fe-2SST18(D1815_20620)Aquimarina sp. AD1
MSDVTIKIKDREGEIHEVQAPTDMAMNLMEVCKAYELPVEGTCGGMAMCASCQCYVLSDH
ELPEMSQDEDLMLAEAFYVEDNSRLGCQIPITPELDGLEVELAPES

>2Fe-2SST18(AAT17_05370)Nonlabens sp. MIC269
MAIDVTITVIDREGEKHELLAPTDMNMNVMEICKSYELPVQATCGGMAMCATCQCYIISD
HDLGERNDDEEAMLWEASFVKDNSRLGCQIPITEELDGLVIELAPEE

>2Fe-2SST18(EJ995_04640)Nonlabens ponticola
MSDIKMTIIDRDGQRHEVDAPTDMNMNVMEVCKAYDLPVQAVCGGMAMCATCQCYIISDH
DLGERNDDEEAMLWEAQNVKDNSRLGCQIPITEDLEGLVIELAPEE

>2Fe-2SST18(2753204332)Nonlabens spongiae JCM 13191

MNDIKMTIIDREGVRHEVEAPTDMNMNVMEVCKAYELPVQAVCGGMAMCATCQCYIISDHDILERNDDEEAMLWEAENVKDNSRLGC
QIPITEDLEGLVIELAPEAD

>2Fe-2SST18(DDD_1815)Nonlabens dokdonensis
MEDINITIIDREGIEHKVLAPTDMNMNLMEVCKSYELPVQAVCGGMAMCATCQCYIISDH
DLGERNDDEQAMLWEADHVKDNSRLGCQIPITEDLDGLIVELAPED

>2Fe-2SST18(2540717113)Nonlabens dokdonensis DSW-6

MEDINITIIDREGIEHKVLAPTDMNMNLMEVCKSYELPVQAVCGGMAMCATCQCYIISDHDLGERNDDEQAMLWEADHVKDNSRLGC
QIPITEDLDGLIVELAPED

>2Fe-2SST18(CW736_06325)Nonlabens sp. MB-3u-79
MSDVNITIIDRVGAAHQVLAPTDMNMNLMEVCKAYELPVQAVCGGMAMCATCQCYIISDH
DLGERNDDEQAMLWEAENVKDNSRLGCQVPITEDLEGLIVQLAPED

>2Fe-2SST18(2629378807)Pontibacter korlensis X14-1T

MPKLKVQNLANLEVEVAEGQTLLKALQAQSTDWMHACGGKGCCTTCRIIILQGMEHTAPLTAAEIRYRDRGRLKDNERLTCQCTLTS
GEITGKVPEQTKLPHMSYSS

>2Fe-2SST18(SGRA_2393)Saprospira grandis
MSDTIKVTVIDRNGQAHELDAPTDMNMNMMELCKAYELPVEGTCGGMALCASCHMYIESD
HELNEPSDDEEDMLDQAFFVEDNSRLGCQIHLRPELEGLVVRLAAADD

>2Fe-2SST18(2504769771)Haliscomenobacter hydrossis O, DSM 1100

MDNMINITLIDREGVEHQLEGPTDMNMNLMELCKAYELPVKGTCGGMALCSTCHVYVLSDHDLHDMSEDEENILDQAFFVKDNSRLG
CQLHLSDELEGLTVQLAPESEN

>2Fe-2SST18(D3Y59_10360)Hymenobacter oligotrophus
MPTLTVQNLPGGPINVSSGQTLLRAVQDAGHDWMHACGAKGRCTTCRIELISGAENLAPPTAHELRYLNTGRLAPNCRLSCQAQLPQ
GEVVGRVPDATQLPHVQYLGDR

>2Fe-2SST18(D770_16920)Flammeovirgaceae bacterium 311
MRVSFVAQITMPSITIQNLDNKTIQVTEMIQSVLQAMGAAGVDWMHACGGKGRCTTCAMRVVEGEENLSTFSEGELRMQRQGRLPKA
YRLACQCIVEKGNVVVRVPETSKLPHMPYTD

Subtype 19
>2Fe-2SST19 (Runsl_2949)Runella slithyformis

MKYKKHVFICTNQKDGGKKCCGAEHGAALVDAFKTSLKEKNLHIDIRAQKSGCLDVCAFG

PALVVYPEGVFYGKVELADVEEIIESHLINDVPVERLALKF

3Fe-4S

Subtype 8
>3Fe-4SST8(EAG08_15720) Chryseobacterium sp. 3008163
METHKYSNGNINVIWQPKKCIHAGVCVKMLPKVYNPKDRPWIKAENATSDELQNQINQCP
SGALTYELNTEK
>3Fe-4SST8(AREALGSMS7_03851) Arenibacter algicola
MDKKENSKEYTNGEITVVWKPSKCIHAGVCVKTLPQVYDPKGKPWIKPEMATTEQLKSQI
ANCPSGALSFYMNGKS
>3Fe-4SST8(D1818_01260) Aquimarina sp. BL5
MRKEYTNGELTIVWKPGKCIHAGVCVKTLPEVYQPNEKPWIQPEMAATEALKSQIDACPS
KALSYYMNTK

>3Fe-4SST8(C5O00_13685) Pukyongia salina
MKLYDNEFTNGEITVNYDPNKCIHAEICAKGLSEVFRTTVIPWIHMDGADSKTIIEQVKK
CPSGALSFCYNKELAAVK

>3Fe-4SST8(KORDIASMS9_04127) Kordia sp. SMS9

MEEKEIIKEYSNEDITVVWKPKTCTHSKNCWKGLLQVFNPQNRLWINMDGASTERIKKQI
AACPSGALSYKSNKEGDQEALQLETKVEALENGPLLVYGTLNITNSDGTKEKRNKTTAFC
RCGASQNKPFCDGAHTDAGFEG
>3Fe-4SST8(DJ013_11780) Arcticibacterium luteifluviistationis
MKSKTYSNNDVTILWQADKCTHAGVCVRTLPNVYDPKVRPWIKPENASGEELIAQVSKCP
SGALSIVNE

4Fe-4S

Subtype 13

>4Fe-4SST13(2758619994)Polaribacter sp. SA4-12

MVVITLQRKKCIGCNYCVELAPAQFQMSKKDGKTVLLHSIEKKGFFTIKSFDESIFDCSNEAKKACPVNIIEVKQI

>4Fe-4SST13(Fjoh_0902)Flavobacterium johnsoniae UW101
MVIVTLQRDKCIGCNYCVEMDPVHFQMSKKDGKSVLIHSQNAKGFYTLKSPNHTIVESCE
LAAKACPVKIITVKET

>4Fe-4SST13(644740302)Flavobacterium johnsoniae UW101, ATCC 17061

MVIVTLQRDKCIGCNYCVEMDPVHFQMSKKDGKSVLIHSQNAKGFYTLKSPNHTIVESCELAAKACPVKIITVKET

>4Fe-4SST13(C7S20_06185)Gramella fulva
MVVVTLQRQKCIGCNYCVEMAPGQFQMSKKDGKSVLLNSNEKKGFFTLKSHDNSIFDPCL
QAQKACPVKIITAKIL
>4Fe-4SST13(C1A40_07150)Tamlana carrageenivorans
MVVVTLQRKKCIGCNYCVELAPNQFQMSKKDGKTVLLHGQDKKGFFTLKSNDELIFDACD
NAAKACPVKIISVKQV
>4Fe-4SST13(C1H87_11645)Flavivirga eckloniae
MVVITLQRNKCIGCNYCVELAPNQFQMSKKDGKSVLLHSKDKKGFFTLKSNDDVIFDDCD
KASKACPVKIISVKSI
>4Fe-4SST13(BW723_03290)Polaribacter reichenbachii
MVVITLQRNKCIGCNYCVEVAPNQFQMSKKDGKSVLLHSTEKKGFYTIKSFDESIFDSTM
EAKKACPVKIIQVKQV

Subtype 14

>4Fe-4SST14(Emtol_0382)Emticicia oligotrophica
MPKIIHFRKNCIGCNACVEIAYNRWRMSKKDGKSVLLGAKEKKGIHQVDILIDEYDENLR
AAEACPVNVIQVYI
>4Fe-4SST14(MY04_2664)Flammeovirga sp. MY04
MSKITITHQRNKCIGCNYCVEFAPDRWVMSKRDGKATLVGGVNKKGFYTTKVSPIELDEN
IKAAEACPVKIIKVKEF
>4Fe-4SST14(EI427_14510)Flammeovirga pectinis
MAKITITHQRDKCIGCNYCVEFAPDRWVMSKKDGKATLVGGVNRKGFYSLKLSPIELEEN
QKAADACPVKIIQVREY

Subtype 15

>4Fe-4SST15(C1A40_10735) Tamlana carrageenivorans
MKTTEYGNREISITYCPFQCQQSNICTQELSDVFQNSVIPWIDPEGSTTEKIIKQIKKCP
SGALKYKLHKKEMAY

2[4Fe-4S]

Subtype 12

>2[4Fe-4S]ST12(2629375234)Pontibacter korlensis X14-1T

MFSLLRSMWLTFLHAFHKRETILYPEQKPILPTRWRGRIVLTRDPDGGERCVACNLCAAACPVDCIALQPSEDEAGRRYPDFFRINFSRCIFCGY
CEEACPTYAIQLIPDFEMGEYNRQNLVYEKKDLLINGQGKYHGYNYYKVAGMAIGGKDKGESENELPPVDIKSLIP

>2[4Fe-4S]ST12(2506618769)Niastella koreensis GR20-10, DSM 17620

MLSHLRTMWLVFIHLFRKKETIEYPEEKVELHPRYRGRIVLTKDPDGGERCVGCYLCAVACPVDCIALQATEDENGRRYPDFFRINFSRCIFCGF
CEDACPTYAIQLIPDFEMADYNRQNLVFEKEDLLIDSQGKYPGYNFYRVAGLEAGVKKKGEGANEEKPVDVHSLLP

>2[4Fe-4S]ST12(644965991)Chitinophaga pinensis UQM 2034, DSM 2588

MISHLRTMWLVFLHLFHKRETIQYPEEKVALRPRWRGRIVLTKDPDGGERCVGCYLCAAACPVDCIALQATEDENGRRYPEFFRINFSRCIFCGY
CEEACPTYAIQLTPDFEMAEYRRQDLVYEKEDLLIDSQGKYPGYNFYKMAGLSAGVKDKGKGENEEAPVDIKSLLP

Subtype 34
>2[4Fe-4S]ST34(2598916424)Sphingobacterium sp. ML3W

MIFKTVFHALKTASKGLSLTVKHLFGARKSRQELDITKDNYFDKQHGIATVQYPKVKMPIPEVARYQLDVEIDDCIVCDLCAKACPVDCIAIEAI
KSPEVIGKTSDGSVKRLYAAKFEIDMAKCMYCGLCTVVCPTECITMTDQYDRSTTKLTDLIYGFSDMTEEQIAERKSDWTKFQAEKEAAKSK

>2[4Fe-4S]ST34(Sph21_2207)Sphingobacterium sp. 21
MLKRTLHAFGTALKGLSITLKHFFAARKARKELDIKQENYFEKQEGVTTIQYPKQEIPIPEVARYQLDVEIDDCIVCDLCAKACPVD
CISIEAIKATEAIGKTSDGSVKRLYAAKFDIDMAKCMYCGLCTVVCPTECITMTDNYDRSFSNLSDLIYQFSEMTDEEIEEKRAELA
RFQAEKEAAKRKS

>2[4Fe-4S]ST34(CA264_01715)Pontibacter actiniarum
MKEAFRAKTGFWSGVKSLASGMRLTWKHFTGARKRRTPEYVSDENYFKQADGLVTLKYPY
EAIPVPDNGRYRLHNEIDDCIVCDLCAKICPVNCITIESVKATEDIDTTSDGTKKRLYAP
TFDIDLAKCCYCGLCTTVCPTDCLTMTPVYDFSEVDIKNMVYHFTDLTPEQAEEKKQQFA
KQQEEMAAAKAAALAARKQG

>2[4Fe-4S]ST34(PKOR_11440)Pontibacter korlensis
MKQHNGFGYVVKSLLSGLSLTWKHFKNARNRRTPDYVSDENYFKQPDGMATLKYPYESIP
VPDNGRYRLHNEIDDCIVCDLCAKICPVNCITIDSVKATEDIGVTSDGTKKRLYAPTFDI
DLAKCCYCGLCTTVCPTDCLTMTPVYDFSEVNIKNMVYHFTNLTPEQAEEKKQLFAKQQE
EIAAAKAAALAARKSNQ

>2[4Fe-4S]ST34(MY04_1150)Flammeovirga sp. MY04
MIKKIGANSSYFGNIIEAIQTSIHGLKLSIIHLKNALSKRRIGNDSFKKDGYFDPESSMV
TLKYPEETLPVPDNGRYKLDLEIDDCIVCDKCAKVCPVDCIEIDPILSQDVIGETSDGTA
KRIYAAKFDIDMAKCCFCGLCTYVCPTECLTMTKSYDFSELDMRELTYSFAEMSEDEVTA
KKVLFEEAQEKKKAEKLKKDTEQSSKPSTSARPKFSPKVIKKK

>2[4Fe-4S]ST34(EI427_07305)Flammeovirga pectinis
MSKKLGANNTYFGNIKEAIQTSKHGLKLTIDHLKDALVNDRRDPISPNDSNYFEKQNSIV
TLKYPQEVLPVPDTGRYKLDLEIDDCIVCDKCAKVCPVDCIEIEPIKSTEVIGETSDGTP
KRIYAAKFDIDMAKCCFCGLCTYVCPTECLTMTKSYDFSELDMRNLTYGFADMSEQEVKQ
KQKIFDLEQEKKRQAKMATTQPTVSVTTEKEIETESSKDKPKPKFRPRVIKK

2[4Fe-4S]Alv
Subtype 11

>2[4Fe-4S]AlvST11(YQ22_16700)Maribacter sp. 1_2014MBL_MicDiv
MAIIITDECINCGACEPECPNTAIYEGADEWRYSDGTSLEGNVVLPDGKEVDAGEVQEPI
SDEVYYIAPDKCTECMGFHEEPQCAAVCPVDCCVPDDDHVETEEVLLAKQRFMHPE

>2[4Fe-4S]AlvST11(D3Y59_05435)Hymenobacter oligotrophus
MAIMITDECINCGACEPECPNNAIYEGGANWRWADGTTLKGTIEVDGGKQVDATNPQVPVSDEYYYIVSDKCTECVGFHEEPQCAAV
CPVDCCVDDPDYRESQEQLLKKKDWLHIA

>2[4Fe-4S]AlvST11(C1H87_14945)Flavivirga eckloniae
MAIIITDECINCDACIVECPNNAIYEPDQEWAYADETALSGSVTLPNGDEADADEMNDPISDEFYYIVPEKCTECKGFHEEPQCASVCPVDCCVP
DEDHVESEETLLEKKAWLHAE

>2[4Fe-4S]AlvST11(Cpin_5947)Chitinophaga pinensis
MAIKITDECINCGACEPECPNNAIYEGGVEWAMADGTSIKGGFVMMDGSTIDADQRNAPI
AVDSYYIVPNKCTECQGFHEEPQCAAVCPVDCCVPDEMYQETVDDLLAKKEKLHM

>2[4Fe-4S]AlvST11(FLA_4313)Filimonas lacunae
MSIKITEECINCGACEPECPNNAIYEGGVEWAIADGTSVKGAFTTSEGTVINADERFAPI
SVDTYYIVPNKCTECQGFHEEPQCAAVCPVDCCVPDEMYQETIDQLMAKKDRLHV

>2[4Fe-4S]AlvST11(D3H65_15130)Paraflavitalea soli
MSIKITEECINCGACEPECPNNAIYEGGVEWAITDGTNVKGPFTLLDGSVVDADQRFAPI
SVDMYYITPNKCTECQGFHEEPQCAAVCPVDCCVPDEMYQETVDELLAKKEKMHL

>2[4Fe-4S]AlvST11(SGRA_1051)Saprospira grandis
MAIIITDECINCGACEPECPNTAIYEGGVEWSYEEGTEVEGDFELHNGKTANAGLMNEPV
SDDFYFIVSDKCTECVGFHEEPQCAAVCPVDCCVPDPEHVEDEATLLKKKAIMHPSEE

>2[4Fe-4S]AlvST11(C7S20_03465)Gramella fulva
MALTITDECINCDACIAECPNNAIYEPDQNWSYSDETVLSGTVTTPSGKEIDADAENEPL
SDEFYFIIAEKCTECKGFHDEPQCASVCPVDCCVPDEDHRETEEELLAKKEWLHGA

>2[4Fe-4S]AlvST11(Fjoh_2464)Flavobacterium johnsoniae UW101
MAIIITDECINCGACEPECPNTAIYEGADDWRYKDGTSLSGTVILPDGTEVDADDAQTPI
SDEIYYIVPGKCTECKGFHDEPQCAAVCPVDCCVPDDNHVEDDETLLNRQAFLHGE

>2[4Fe-4S]AlvST11(AXE80_05485)Wenyingzhuangia fucanilytica
MAIIITDECINCGACEPECPNNAIYEGAEEWKYSEGTSLEGDFVLPNGNNGNADEEQEPI
SDEVYYIVPDKCTECKGFHDEPQCAAVCPVDCCVPDEDVVETEEELLAKQAFLHKD

>2[4Fe-4S]AlvST11(CW732_13615)Olleya sp. Bg11-27
MAIIITDECINCGACEPECPNTAIYEGADDWRYKDGTSLNGNLVLTDGKEVDADEAQEPI
SDEIYYIVPDKCTECVGFHEEPQCAAVCPVDCCVPDDEHVESKDTLLGKQKFMHPDG

>2[4Fe-4S]AlvST11(DZC78_01635)Olleya aquimaris
MAIIITDECINCGACEPECPNTAIYEGADDWRYKDGTSLDGSLVLPNGKEVDADEAQEPI
SDEIYYIVPDKCTECVGFHEEPQCAAVCPVDCCVPDDDNVEAEDVLLGKQKFMHPNG

>2[4Fe-4S]AlvST11(C1A40_00540)Tamlana carrageenivorans
MAIIITDECINCGACEPECPNTAIYEGADDWRYKDGTSLDGKVVLTNGTEVDADEAQEPI
SDEVYYIVPDKCTECKGFHDEPQCAAVCPVDCCVPDDDHVETEDELLAKQRFMHPDG

>2[4Fe-4S]AlvST11(C1H87_03310)Flavivirga eckloniae
MAIIITDECINCGACEPECPNTAIYEGADDWRYKDGTSLEGAVVLTNGNEVDADEAQEPI
SDEVYYIVPDKCTECKGFHDEPQCAAVCPVDCCVPDEDVVETEDELLAKQRFMHPDG

>2[4Fe-4S]AlvST11(ZOBELLIA_4118)Zobellia galactanivorans
MAIVITDECINCGACEPECPNTAIYEGADEWRYSDGTSLEGDVVLPNGKAVNADEVQEPI
SDEIYYISPDKCTECMGFHEEPQCAAVCPVDCCVPDDEHVESEETLLAKQKFMHPDS

>2[4Fe-4S]AlvST11(D1013_11715)Euzebyella marina
MAIVITDECINCGACEPECPNTAIYEGADEWRYSDGTSLEGDVVLPSGKAVNADEAQEPI
SDEIYYISPDKCTECMGFHEEPQCAAVCPVDCCVPDDDHEESEETLLAKQAFMHPNG

>2[4Fe-4S]AlvST11(EQY75_06745)Muriicola sp. MMS17-SY002
MAIIITDECINCGACEPECPNTAIYEGAEEWRYSDGTSLTGDVVLPDGKKVNADEVQEPV
SDEIYYIAPDKCTECMGFHEEPQCAAVCPVDCCVPDEDHVESEETLLSKQSFMHPDG

>2[4Fe-4S]AlvST11(EJ994_05155)Maribacter sp. MJ134
MAIIITDECINCGACEPECPNTAIYEGADEWRYSDGTSLEGDVVLPNGKAVNADVVQEPI
SDEVYYIAPDKCTECMGFHEEPQCAAVCPVDCCVPDEDNVETEEVLLGKQAFMHPDG

>2[4Fe-4S]AlvST11(Celal_4200)Cellulophaga algicola
MAIIITDECINCGACEPECPNTAIYEGADEWRYSDGTSLKGQVVLPNGKEIDADVVQEPI
SDEVYYISPDKCTECVGFHEEPQCAAVCPVDCCVPDDDHVETEDVLLGKQKFMHPEG

>2[4Fe-4S]AlvST11(M667_17355)Cellulophaga baltica NN016038
MAIIITDECINCGACEPECPNTAIYEGADEWRYSDGTSLKGQVVLPNGKEVDADDVQEPI
SDEVYYISPDKCTECMGFHEEPQCAAVCPVDCCVPDDDHVETEEVLLEKQKFMHPEG

>2[4Fe-4S]AlvST11(M666_17355)Cellulophaga baltica 18

MAIIITDECINCGACEPECPNTAIYEGADEWRYSDGTSLKGQVVLPNGKEVDADDVQEPI

SDEVYYISPDKCTECMGFHEEPQCAAVCPVDCCVPDDDHVETEEVLLEKQKFMHPEG

>2[4Fe-4S]AlvST11(YQ22_16700)Maribacter sp. 1_2014MBL_MicDiv
MAIIITDECINCGACEPECPNTAIYEGADEWRYSDGTSLEGNVVLPDGKEVDAGEVQEPI
SDEVYYIAPDKCTECMGFHEEPQCAAVCPVDCCVPDDDHVETEEVLLAKQRFMHPE

>2[4Fe-4S]AlvST11(Celly_2988)Cellulophaga lytica DSM 7489
MAIIITDECINCGACEPECPNTAIYEGADDWRYADGTSLEGTIVLPNGKEVDAEESQEPI
SDEIYYISPDKCTECKGFHEEPQCAAVCPVDCCVPDDDIVETEEELLAKQRFMHPEG

>2[4Fe-4S]AlvST11(IX49_15060)Cellulophaga lytica HI1
MAIIITDECINCGACEPECPNTAIYEGADDWRYADGTSLEGTIVLPNGKEVDAEESQEPI
SDEIYYISPDKCTECKGFHEEPQCAAVCPVDCCVPDDDIVETEEELLAKQRFMHPEG

>2[4Fe-4S]AlvST11(SB49_04640)Sediminicola sp. YIK13
MAIIITDECINCGACEPECPNTAIYEGADDWRYSDGTSLSGKVVLPNGSEVDADEVQEPI
SDEIYYISPDKCTECMGFHEEPQCAAVCPVDCCVPDDSHVETEEELLAKQRFMHPEG

>2[4Fe-4S]AlvST11(P700755_003846)Psychroflexus torquis
MAIKITDECINCGACESECPNTAIYEGGDDWRHADGTSLKGDIVLPNGKEANAEEAQEPV
SDELYYIAADKCTECMGFHEEPQCAAVCPVDCCVPDDEHVESEETLLGKQSFMHNK

>2[4Fe-4S]AlvST11(EAG08_20415)Chryseobacterium sp. 3008163
MAIKITDECINCGACEPECPNNAIYEGAVDWKASEGTELKGTVTLPSGLTVDADAPQEPV
NDDVYFIVTDKCTECKGFHEEPQCAAVCPVDCCVPDEDHVESEEALLNKKAFLHGE

>2[4Fe-4S]AlvST11(KORDIASMS9_03838)Kordia sp. SMS9
MAIIITDECINCGACEPECPNTAIYEGADDWRYADGTSLTGTIVLPSGKEVDAEEAQEPI
SDEIYYISPDKCTECMGFHEEPQCAAVCPVDCCVPDEDIVETEEELLGKQAFMHPEE

>2[4Fe-4S]AlvST11(TJEJU_3525)Tenacibaculum jejuense
MAIIITDECINCGACEPECPNTAIYEGAEDWNYGQGTSLEGDIELLNGIKLNAEDDQEAI
SDEYYFIVPDKCTECKGFHEEPQCAAVCPVDCCVPDENHVETEEELLNKQQFLHN

>2[4Fe-4S]AlvST11(AREALGSMS7_04246)Arenibacter algicola
MAIIITDECINCGACEPECPNTAIYEGADDWRYSDGTLLKGDVVLPNGKAVNADESQEPV
SDEIYFIVPDKCTECKGFHDEPQCAAVCPVDCCVPDEDHVESEEVLLGKQRFMHPE

>2[4Fe-4S]AlvST11(D1818_03640)Aquimarina sp. BL5
MAIIITDECINCGACEPECPNTAIYEGADDWRYADGTDLEGDVVLPGGKAINSEEAQEPV
SDEIYYIVPDKCTECKGFHEEPQCAAVCPVDCCVPDEDVVETEEFLLQKQAFMHKE

>2[4Fe-4S]AlvST11(D1815_03950)Aquimarina sp. AD1
MAIIITDECINCGACEPECPNTAIYEGADDWRYADGTDLEGDVVLPGGKAVNSEEAQEPV
SDEIYYIVPDKCTECKGFHEEPQCAAVCPVDCCVPDEDIVETEEFLLQKQAFMHKE

>2[4Fe-4S]AlvST11(D1816_03330)Aquimarina sp. AD10
MAIIITDECINCGACEPECPNTAIYEGADDWRYSDGTDLEGDVVLPGGKGVNADEAQEPV
SDELYYIVPDKCTECKGFHEEPQCAAVCPVDCCVPDEDVVETEEFLLEKQAFMHKE

>2[4Fe-4S]AlvST11(BW723_13210)Polaribacter reichenbachii
MAIIITDECINCGACEPECPNTAIYEGADDWKYSDGTDLSGDIVLPGGKAANADEDQEPV
SDEIYYIVPDKCTECKGFHDEPQCAAVCPVDCCVPDEDVVETEEFLLEKQAFMHNE

>2[4Fe-4S]AlvST11(DDD_3368)Nonlabens dokdonensis
MAIIITDECINCGACEPECPNTAIYEAADDWRYADGTDLDGNVVLPSGKEVDANETQEPI
SDEFYYIAPDKCTECVGFHEEPQCAAVCPVDCCVPDDDVVETEAELRAKQAFMHKS

>2[4Fe-4S]AlvST11(AAT17_01460)Nonlabens sp. MIC269
MAIIITDECINCGACEPECPNTAIYEAADDWRYADGTDLDGQIVLPNGKEVDANETQEPV
SDEFYYIVPDKCTECVGFHEEPQCAAVCPVDCCVPDEDIVETEEELKAKQAFMHKE

>2[4Fe-4S]AlvST11(CW736_10985)Nonlabens sp. MB-3u-79
MAIIITDECINCGACEAECPNTAIYEPADDYRYADGTDLDGNVVLPSGKEIDANETQEPV
SDEFYYIVPDKCTECVGFHEEPQCAAVCPVDCCVPDDNVVETKEELEAKQAFMHKG

>2[4Fe-4S]AlvST11(EJ995_09580)Nonlabens ponticola
MAIIITDECINCGACEPECPNTAIYEAADDWRYADGTDLDGNVVLPSGKEVDANETQEPV
SDEFYYIVPDKCTECVGFHEEPQCAAVCPVDCCVPDEDVVEDEATLRAKQAFMHKD

>2[4Fe-4S]AlvST11(TJEJU_2222)Tenacibaculum jejuense
MAIIITDECINCGACEPECPNTAIYEGAEEWKYADGTDLEGNIVLPNGNNANADEDQEPI
SDEIYYIVADKCTECKGFHEEPQCAAVCPVDCCVPDENNVETEEELLGKQRFMHND

>2[4Fe-4S]AlvST11(Krodi_2456)Dokdonia sp. 4H-3-7-5
MAIIITDECINCGACEPECPNTAIYEGADDWRYADGTDLEGNVVLPNGKEVDANEAQQPV
SDEIYYIIPDKCTECKGFHDEPQCAAVCPVDCCVPDDEHVETEEVLLAKQSFMHKDA

>2[4Fe-4S]AlvST11(I597_2315)Dokdonia donghaensis
MAIIITDECINCGACEPECPNTAIYEGADDWRYADGTDLEGNVVLPNGKEVDANEAQEPV
SDEIYYIIPDKCTECKGFHEEPQCAAVCPVDCCVPDDDHVETDEVLLAKQAFMHKDA

>2[4Fe-4S]AlvST11(DCS32_15045)Dokdonia sp. Dokd-P16
MAIIITDECINCGACEPECPNTAIYEGADDWRYADGTDLEGNVILPNGKEVDANEAQQPV
SDEIYYIIPDKCTECKGFHDEPQCAAVCPVDCCVPDDEHVETEEVLLAKQSFMHKDA

>2[4Fe-4S]AlvST11(ZPR_2137)Zunongwangia profunda
MAIIITDECINCGACEPECPNTAIYEGADDWRYADGTELSGDIVLPSGTEANAEEAQEPV
SDEYYYIVPDKCTECKGFHEEPQCAAVCPVDCCVPDEDHVETEEELLAKQKFMHE

>2[4Fe-4S]AlvST11(GFO_1472Gramella forsetii
MAIVITDECINCGACEPECPNTAIYEGADDWRYADGTDLEGDVVLPGGKEADANEAQEPI
SDELYYIVPDKCTECQGFHEEPQCAAVCPVDCCVPDDEHVESEEELLAKQRFMHHDE

>2[4Fe-4S]AlvST11(LPB144_04165)Gramella salexigens
MAIVITDECINCGACEPECPNTAIYEGADDWRYADGTDLEGNVVLPNGKEADANEAQEPI
SDEIYYIVPDKCTECKGFHEEPQCAAVCPVDCCVPDDEHVESDEELLAKQRFMHEED

>2[4Fe-4S]AlvST11(GRFL_3157)Gramella flava
MAIVITDECINCGACEPECPNTAIYEGADDWRYADGTDLEGNVVLPNGHEANAEEPQEPI
SDEIYYIVPDKCTECKGFHEEPQCAAVCPVDCCVPDDDHVESEEELLEKQRFMHHE

>2[4Fe-4S]AlvST11(C7S20_14835)Gramella fulva
MAIVITDECINCGACEPECPNTAIYEGADDWRYADGTDLEGNVVLPNGKEANAEEAQEPV
SDEIYYIVPDKCTECKGFHEEPQCAAVCPVDCCVPDEDHVETEEELLAKQRFMHNQD

>2[4Fe-4S]AlvST11(AO058_13050)Salegentibacter sp. T436
MAIVITDECINCGACEPECPNTAIYEGADDWRYADGTDLEGNVVLPNGKEADANEAQEPI
SDEIYYIVPDKCTECKGFHEEPQCAAVCPVDCCVPDDEHVESEEELLAKQKFMHENE

>2[4Fe-4S]AlvST11(2506616094)Niastella koreensis GR20-10, DSM 17620

MALKITTDCIVCGACEPECPNNAIYDAGVEWRMADGTTVKDGYTLLNGTITDSATNQAPLSDLYYYIVPDKCTECQGFHEQPQCVDACPVDSCVP
DEIYQETVEELLAKKEKLHS

>2[4Fe-4S]AlvST11(Sph21_3001)Sphingobacterium sp. 21
MAIKITDECINCGACEPECPNNAIYDAGMAWRFSDGTELHGVIDFGDGTTLDADEAQEALSDEIYYIVSDKCTECVGFHDEPQCAAV
CPVDCCVDDEDVRETQEELLAKKAWLHAEG

>2[4Fe-4S]AlvST11(2758370175)Psychroflexus torquis ATCC 700755

MAIKITDECINCGACESECPNTAIYEGGDDWRHADGTSLKGDIVLPNGKEANAEEAQEPVSDELYYIAADKCTECMGFHEEPQCAAV
CPVDCCVPDDEHVESEETLLGKQSFMHNK

>2[4Fe-4S]AlvST11(2620658017)Zobellia galactanivorans DsijT

MAIVITDECINCGACEPECPNTAIYEGADEWRYSDGTSLEGDVVLPNGKAVNADEVQEPISDEIYYISPDKCTECMGFHEEPQCAAV
CPVDCCVPDDEHVESEETLLAKQKFMHPDS

>2[4Fe-4S]AlvST11(6,5E+08)Cellulophaga algicola IC166, DSM 14237

MAIIITDECINCGACEPECPNTAIYEGADEWRYSDGTSLKGQVVLPNGKEIDADVVQEPISDEVYYISPDKCTECVGFHEEPQCAAV
CPVDCCVPDDDHVETEDVLLGKQKFMHPEG

>2[4Fe-4S]AlvST11(646746259)Zunongwangia profunda SM-A87

MAIIITDECINCGACEPECPNTAIYEGADDWRYADGTELSGDIVLPSGTEANAEEAQEPVSDEYYYIVPDKCTECKGFHEEPQCAAV
CPVDCCVPDEDHVETEEELLAKQKFMHE

>2[4Fe-4S]AlvST11(2,724E+09)Cellulophaga lytica DAU203

MAIIITDECINCGACEPECPNTAIYEGADDWRYADGTSLEGTIVLPNGKEVDAEESQEPISDEIYYISPDKCTECKGFHEEPQCAAV
CPVDCCVPDDDIVETEEELLAKQRFMHPEG

>2[4Fe-4S]AlvST11(649972079)Cellulophaga lytica LIM-21, DSM 7489

MAIIITDECINCGACEPECPNTAIYEGADDWRYADGTSLEGTIVLPNGKEVDAEESQEPISDEIYYISPDKCTECKGFHEEPQCAAV
CPVDCCVPDDDIVETEEELLAKQRFMHPEG

>2[4Fe-4S]AlvST11(639718332)Gramella forsetii KT0803

MAIVITDECINCGACEPECPNTAIYEGADDWRYADGTDLEGDVVLPGGKEADANEAQEPISDELYYIVPDKCTECQGFHEEPQCAAV
CPVDCCVPDDEHVESEEELLAKQRFMHHDE

>2[4Fe-4S]AlvST11(2719632980)Gramella sp. LPB0144

MAIVITDECINCGACEPECPNTAIYEGADDWRYADGTDLEGNVVLPNGKEADANEAQEPISDEIYYIVPDKCTECKGFHEEPQCAAV
CPVDCCVPDDEHVESDEELLAKQRFMHEED

>2[4Fe-4S]AlvST11(2758560734)Tenacibaculum jejuense KCTC 22618

MAIIITDECINCGACEPECPNTAIYEGAEDWNYGQGTSLEGDIELLNGIKLNAEDDQEAISDEYYFIVPDKCTECKGFHEEPQCAAV
CPVDCCVPDENHVETEEELLNKQQFLHN

>2[4Fe-4S]AlvST11(TJEJU_3525)Tenacibaculum jejuense
MAIIITDECINCGACEPECPNTAIYEGAEDWNYGQGTSLEGDIELLNGIKLNAEDDQEAI
SDEYYFIVPDKCTECKGFHEEPQCAAVCPVDCCVPDENHVETEEELLNKQQFLHN

>2[4Fe-4S]AlvST11(644741892)Flavobacterium johnsoniae UW101, ATCC 17061

MAIIITDECINCGACEPECPNTAIYEGADDWRYKDGTSLSGTVILPDGTEVDADDAQTPISDEIYYIVPGKCTECKGFHDEPQCAAV
CPVDCCVPDDNHVEDDETLLNRQAFLHGE

>2[4Fe-4S]AlvST11(2718722668)Wenyingzhuangia fucanilytica CZ1127

MAIIITDECINCGACEPECPNNAIYEGAEEWKYSEGTSLEGDFVLPNGNNGNADEEQEPISDEVYYIVPDKCTECKGFHDEPQCAAV
CPVDCCVPDEDVVETEEELLAKQAFLHKD

>2[4Fe-4S]AlvST11(2758559443)Tenacibaculum jejuense KCTC 22618

MAIIITDECINCGACEPECPNTAIYEGAEEWKYADGTDLEGNIVLPNGNNANADEDQEPISDEIYYIVADKCTECKGFHEEPQCAAV
CPVDCCVPDENNVETEEELLGKQRFMHND

>2[4Fe-4S]AlvST11(CW736_10985)Nonlabens sp. MB-3u-79
MAIIITDECINCGACEAECPNTAIYEPADDYRYADGTDLDGNVVLPSGKEIDANETQEPV
SDEFYYIVPDKCTECVGFHEEPQCAAVCPVDCCVPDDNVVETKEELEAKQAFMHKG

>2[4Fe-4S]AlvST11(2540718691)Nonlabens dokdonensis DSW-6

MAIIITDECINCGACEPECPNTAIYEAADDWRYADGTDLDGNVVLPSGKEVDANETQEPISDEFYYIAPDKCTECVGFHEEPQCAAV
CPVDCCVPDDDVVETEAELRAKQAFMHKS

>2[4Fe-4S]AlvST11(2753205917)Nonlabens spongiae JCM 13191

MAIIITDECINCGACEPECPNTAIYEAADDWRYADGTDLDGNVVLPSGKEVDANETQEPVSDEFYYIVPDKCTECVGFHEEPQCAAV
CPVDCCVPDEDIVETEAELRAKQSFMHKN

>2[4Fe-4S]AlvST11(2758618013)Polaribacter sp. SA4-12

MAIIITDECINCGACEPECPNTAIYEGADDWKYSDGTDLKGNAVLPNGKSVNADEDQEPVSDEIYYIVPDKCTECKGFHDEPQCAAV
CPVDCCVPDDDVVETEEELLAKQSFMHNE

>2[4Fe-4S]AlvST11(2597963859)Dokdonia sp. PRO95

MAIIITDECINCGACEPECPNTAIYEGADDWRYADGTDLDGTVVLPNGKEVDANEAQEPVSDEIYYIIPDKCTECKGFHDEPQCAAV
CPVDCCVPDDDHVETDEVLLAKQSFMHKDA

>2[4Fe-4S]AlvST11(2,51E+09)Aequorivita sublithincola QSSC9-3, DSM 14238

MAIIITDECINCGACEPECPNTAIYEGADDWRYADGTDLDGKVVLPNGKEVDANETQEPVSDEIYFIVADKCTECKGFHDEPQCAAV
CPVDCCVPDEDHVETEEELLGKQAFMHKK

>2[4Fe-4S]AlvST11(2598914115)Sphingobacterium sp. ML3W

MAIKITDECINCGACEPECPNNAIYDAGVSWKFSDGTALEGVIDFGDGVTLDAEDSQAAISDEVYYIVSDKCTECVGFHDEPQCAAV
CPVDCCVDDEEVRESEEELLAKKSWLHAE

>2[4Fe-4S]AlvST11(644936856)Pedobacter heparinus HIM 762-3, DSM 2366

MAIKITDECINCGACEPECPNNAIYDAGTAWRFSDGTNLNGIIDFGGKEMDAESAQEAVSDEVYYIVSDKCTECKGFHDEPQCAAVC
PVDCCVDDEDVRETEEELLAKKAWLHQEG

>2[4Fe-4S]AlvST11(2506616093)Niastella koreensis GR20-10, DSM 17620

MAIKITEECINCGACEPECPNNAIYEGGVEWRVADGTTVKGSFVLLDGSIIDADTANAPISVDTYYIVPNKCTECQGFHEEPQCAAV
CPVDCCVPDEMYVETVEQLMDKKGKLHI

>2[4Fe-4S]AlvST11(644968086)Chitinophaga pinensis UQM 2034, DSM 2588

MAIKITDECINCGACEPECPNNAIYEGGVEWAMADGTSIKGGFVMMDGSTIDADQRNAPIAVDSYYIVPNKCTECQGFHEEPQCAAV
CPVDCCVPDEMYQETVDDLLAKKEKLHM

>2[4Fe-4S]AlvST11(2504770455)Haliscomenobacter hydrossis O, DSM 1100

MAIMITDDCINCGACEPECPNNAIYEGGVEWAISDGTSITDDYTLEDGSTVDASDKQNPVSDEFYYIVPDKCTECVGFHEEPQCAAV
CPVDCCVPDPEREEDEAVLLGRKERLHL

>2[4Fe-4S]AlvST11(D770_26070)Flammeovirgaceae bacterium 311
MAIMITDECINCGACEPECPNTAIYEGGIEWTWAGGTSLEEVELEDGANVDAHAPQPPLS
DEFYYIVSGKCTECMGFHEEPQCAAVCPVDCCVPDPDYVEAEEELLAKKAWMHNE

>2[4Fe-4S]AlvST11(2629374622)Pontibacter korlensis X14-1T

MAIMITDECINCGACEPECPNTAIYEGGAEWTWGGGTALTEVEIDGGEVVPGDAPQTPISDEFYYIVSDKCTECMGFHEEPQCAAVC
PVDCCVDDPDYRETEEELLAKKSWLHQEA

>2[4Fe-4S]AlvST11(2506489581)Emticicia oligotrophica GPTSA100-15, DSM 17448

MAIMITDECINCGACEPECPNTAIYEGGVEWTWSGGTKLTEVELEDGTVIGGKDPMSPVSDEFYYIVPDKCTECHGFHEEPQCAAVC
PVDCCVPDPDHEEDDDTLLAKKAWLHAEA

>2[4Fe-4S]AlvST11(644928318)Dyadobacter fermentans NS114, DSM 18053

MITDECINCGACEPECPNTAIYEGGVEWTWGDGTSLDEVDFGDGTIVSGKEKQSPVSDEFYYIVTDKCTECVGFHEEPQCAAVCPVD
CCVPDPDNEEEEETLLAKKAWMHGE

>2[4Fe-4S]AlvST11(646494940)Spirosoma linguale DSM 74

MAIMITDECINCGACEPECPNTAIYEGGVEWTWGGGTELTEVDFGDGTVVSGKAPQAPVSNEFYYIVSDKCTECMGFHEEPQCAAVC
PVDCCVPDPEHVEDEEILLAKKAWLHAEA

>2[4Fe-4S]AlvST11(2600232362)Spirosoma radiotolerans DG5A (Spirosoma radiotolerans genome sequence)

MITDECINCGACEPECPNTAIYEGGVEWTWGGGTELDEVDFGDGTIVSGKAPQAPVSNEFYYIVADKCTECMGFHEEPQCAAVCPVD
CCVPDPDHVEEEETLLAKKAWLHAEA

Subtype 12
>2[4Fe-4S]AlvST12(AY601_0116)Pedobacter cryoconitis
MAIKITDECINCGACEPECPNNAIYDAGTAWRFSDGTNLNGIIDFGDQEVDAGAAQEAVSDEVYYIVSDKCTECKGFHDEPQCAAVCPVDCCVDD
EDIRETEEELLKKKAWLHQEN

>2[4Fe-4S]AlvST12(AQ505_05310)Pedobacter sp. PACM 27299
MAIKITDECINCGACEPECPNNAIYDAGTAWRFSDGTNLNGIIDFGGKELDAEASMEAGSDEVYYIVSDKCTECKGFHDEPQCAAVCPVDCCVDD
EDVRETEEELLAKKAWLHQEN

>2[4Fe-4S]AlvST12(AUC43_13390)Hymenobacter sedentarius
MAIMITDECINCGACEPECPNNAIYEGGAQWRWADGTTLKEVATADGTVVSGTAPQTPVS
DEYYYIVSDKCTECVGFHEEPQCAAVCPVDCCVDDPDYRETRERLLEKKDWLHIAA

>2[4Fe-4S]AlvST12(A0257_01460)Hymenobacter sp. PAMC 26554
MAIMITDECINCGACEPECPNTAIYEGGAQWRWADGTTLKEVQTIDGHLAGGTEPQRPVS
NEYYYIVTDKCTECVGFHEEPQCAAVCPVDCCVDDPDHRESRERLTQKQQWLHKAA

>2[4Fe-4S]AlvST12(AXW84_08695)Hymenobacter sp. PAMC 26628
MAIMITDECINCGACEPECPNNAIYEGGAQWRWADGTTLKQVETADGTTVAGTAPQTPVS
DEYYYIVSDKCTECVGFHEEPQCAAVCPVDCCVDDPDLRETHERLLEKKAWLHLAA

>2[4Fe-4S]AlvST12(DDQ68_03635)Hymenobacter nivis
MAIMITDECINCGACEPECPNNAIYEGGAQWRWADGTTLKQVETADGATVAGTAPQTPVS
DEYYYIVSDKCTECVGFHEEPQCAAVCPVDCCVDDPDLRETRERLLEKKGWLHLVA

>2[4Fe-4S]AlvST12(N008_00785)Hymenobacter sp. APR13
MAIMITDECINCGACEPECPNTAIYEGGAAWRWSDGTTLKEVTVDGGQTVSGVAPQTPIS
DEYYYIVSDKCTECVGFHEEPQCAAVCPVDCCVDDPDYRESQEKLTAKKEWLHS

>2[4Fe-4S]AlvST12(PK28_09160)Hymenobacter sp. DG25B
MAIMITDECINCGACEPECPNNAIYEGGAQWRWADGTALKEVTVDGGATVSGTAPQTPVS
DEYYYIVSDKCTECVGFHEEPQCAAVCPVDCCVDDPDYRESQDALLKKKEWLHAEVS

>2[4Fe-4S]AlvST12(AM218_07200)Hymenobacter sp. DG25A
MAIMITDECINCGACEPECPNNAIYEGGAQWRWADGTALKEVTVDGGETVSGTAPQTPVS
DEYYYIVSDKCTECVGFHEEPQCAAVCPVDCCVDDPDYRESQDALLKKKEWLHAEAS

>2[4Fe-4S]AlvST12(MY04_1257)Flammeovirga sp. MY04
MAIIITDECINCGACEPECPNTAIYEGGVEWTWGDGTSLTSIEMEDGSSIDGSEDQEPVS
DEFYYIVPDKCTECIGFHEEPQCAAVCPVDCCVDDPDYRETEEELTAKKEWLHDE

>2[4Fe-4S]AlvST12(EI427_07665)Flammeovirga pectinis
MAIIITDECINCGACEPECPNTAIYEGGVTWSWGGGTELKSVELEDGNSIDGEDEQEPVS
DEFYYIVPDKCTECIGFHEEPQCAAVCPVDCCVDDPDYRETEDELLAKKQWLHDE

>2[4Fe-4S]AlvST12(DR864_22565)Hymenobacter nivis
MAIMITDECINCGACEPECPNTAIYEGGVEWTYAGGTHLEEVDYGDGTVVDAKVKQSPVS
NEFYYIVTDKCTECMGFHEEPQCAAVCPVDCCVPDPENVEDEETLLAKKAWMHGE

>2[4Fe-4S]AlvST12(CA264_00950)Pontibacter actiniarum
MAIMITDECINCGACEPECPNTAIYEGGMEWTWGGGTELKEVEIEDGEVIPGDAAQQPIS
DEFYYIVSDKCTECMGFHEEPQCAAVCPVDCCVDDPDYRETEEELLAKKDWLHQAS

>2[4Fe-4S]AlvST12(Emtol_4090)Emticicia oligotrophica
MAIMITDECINCGACEPECPNTAIYEGGVEWTWSGGTKLTEVELEDGTVIGGKDPMSPVS
DEFYYIVPDKCTECHGFHEEPQCAAVCPVDCCVPDPDHEEDDDTLLAKKAWLHAEA

>2[4Fe-4S]AlvST12(PKOR_10720)Pontibacter korlensis
MAIMITDECINCGACEPECPNTAIYEGGAEWTWGGGTALTEVEIDGGEVVPGDAPQTPIS
DEFYYIVSDKCTECMGFHEEPQCAAVCPVDCCVDDPDYRETEEELLAKKSWLHQEA

>2[4Fe-4S]AlvST12(D770_26070)Flammeovirgaceae bacterium 311
MAIMITDECINCGACEPECPNTAIYEGGIEWTWAGGTSLEEVELEDGANVDAHAPQPPLS
DEFYYIVSGKCTECMGFHEEPQCAAVCPVDCCVPDPDYVEAEEELLAKKAWMHNE

>2[4Fe-4S]AlvST12(Dfer_0441)Dyadobacter fermentans
MITDECINCGACEPECPNTAIYEGGVEWTWGDGTSLDEVDFGDGTIVSGKEKQSPVSDEF
YYIVTDKCTECVGFHEEPQCAAVCPVDCCVPDPDNEEEEETLLAKKAWMHGE

>2[4Fe-4S]AlvST12(SD10_16460)Spirosoma radiotolerans

MAIMITDECINCGACEPECPNTAIYEGGVEWTWGGGTELDEVDFGDGTIVSGKAPQAPVS
NEFYYIVADKCTECMGFHEEPQCAAVCPVDCCVPDPDHVEEEETLLAKKAWLHAEA

>2[4Fe-4S]AlvST12(CWM47_07245)Spirosoma pollinicola
MAIMITDECINCGACEPECPNTAIYEGGVEWTWGGGTELNEVDFGDGTVVSGKAPQAPVS
NEFYYIVSDKCTECMGFHEEPQCAAVCPVDCCVPDPEHVEEEDTLLAKKAWLHAEA

>2[4Fe-4S]AlvST12(DTQ70_00090)Runella sp. SP2
MAIMITDECINCGACEPECPNTAIYEGGVEWTFAGGTALEEIDYGDGTVVNAKVKQKPVS
DEFYYIVTDKCTECVGFHEEPQCAAVCPVDCCVPDPDNEEDEETLLAKKAWMHGE

>2[4Fe-4S]AlvST12(2505789837)Runella slithyformis LSU4, DSM 19594
MFFDYECKYPKTKRYSAIMAIMITDECINCGACEPECPNTAIYEGGVEWTYAGGTHLEEVDYGDGTVVDAKVKQAPVSNEFYYIVTDKCTECMGF
HEEPQCAAVCPVDCCVPDPENVEDEETLLAKKAWMHGE

>2[4Fe-4S]AlvST12(Runsl_0595)Runella slithyformis
MFFDYECKYPKTKRYSAIMAIMITDECINCGACEPECPNTAIYEGGVEWTYAGGTHLEEVDYGDGTVVDAKVKQAPVSNEFYYIVTDKCTECMGF
HEEPQCAAVCPVDCCVPDPENVEDEETLLAKKAWMHGE

>2[4Fe-4S]AlvST12(FAES_2953)Fibrella aestuarina
MAIMITEECINCGACEPECPNTAIYEGGVEWTWGGGTALDVVDFGDGTVVSGKAPQTPVS
NEFYYIVTDKCTECKGFHEEPQCAAVCPVDCCVPDPEHEEDEDTLLAKKAWLHAEE

>2[4Fe-4S]AlvST12(A6C57_23615)Fibrella sp. ES10-3-2-2
MAIMITEECINCGACEPECPNTAIYEGGVEWTWGGGTALDVVDFGDGTVVSGKSPQTPVS
SEFYYIVTDKCTECKGFHEEPQCAAVCPVDCCVPDPEHEEDEETLLAKKAWLHAEE

>2[4Fe-4S]AlvST12(DJ013_02005)Arcticibacterium luteifluviistationis
MAIIITDECINCGACEPECPNTAIYEGGIEWTWAGGTALEEVDFGDGTVIDAKDDQEPVS
DEFYYIVSDKCTECMGFHEEPQCAAVCPVDCCVPDPDKVEEEETLLAKKAWLHAEEA

>2[4Fe-4S]AlvST12(Slin_1345)Spirosoma linguale
MAIMITDECINCGACEPECPNTAIYEGGVEWTWGGGTELTEVDFGDGTVVSGKAPQAPVS
NEFYYIVSDKCTECMGFHEEPQCAAVCPVDCCVPDPEHVEDEEILLAKKAWLHAEA

>2[4Fe-4S]AlvST12(AWR27_05870)Spirosoma montaniterrae
MAIMITDECINCGACEPECPNTAIYEGGVEWTWGGGTELTEVDFGDGTVISGKAPQAPVSNEFYYIVSDKCTECMGFHEEPQCAAVCPVDCCVPD
PDQVEDEETLLAKKAWLHAEA
