# Supplementary material for: Contrasting Health Effects of Bacteroidetes and Firmicutes Lies in Their Genomes: Analysis of P450s, Ferredoxins, and Secondary Metabolite Clusters
Source: Int J Mol Sci. 2022 May 2;23(9):5057. doi: 10.3390/ijms23095057 (PMC9100364; doi:10.3390/ijms23095057)
Supplement: Supplementary file 1 [file ijms-23-05057-s001.zip › Supplementary information.pdf]

Article

Contrasting Health Effects of *Bacteroidetes* and *Firmicutes* Lies in Their Genomes: Analysis of P450s, Ferredoxins, and Secondary Metabolite Clusters

Bridget Valeria Zinhle Nkosi [1], Tiara Padayachee [1], Dominik Gront [2], David R. Nelson [3,*] and Khajamohiddin Syed [1,*]

[1] Department of Biochemistry and Microbiology, Faculty of Science and Agriculture, University of Zululand, KwaDlangezwa 3886, South Africa; brilenhle@gmail.com (B.V.Z.N.); teez07padayachee@gmail.com (T.P.)
[2] Biological and Chemical Research Center, Faculty of Chemistry, University of Warsaw, Pasteura 1, 02-093 Warsaw, Poland; dgront@gmail.com
[3] Department of Microbiology, Immunology and Biochemistry, University of Tennessee Health Science Center, Memphis, TN 38163, USA; drnelson1@gmail.com
* Correspondence: drnelson1@gmail.com (D.R.N.); khajamohiddinsyed@gmail.com (K.S.); Tel.: +1901-448-8303 (D.R.N.); +27-035-902-6857 (K.S.)

Table of Contents

Table S1. Information on *Bacteroidetes* species and their respective genera was used in the study. Species abbreviations, their genome IDs (GenBank) and presence or absence of P450s in different species, and the number of P450s were presented in the table. 2

Table S2. Comparative analysis of ferredoxins in *Bacteroidetes* species. 16

Table S3. Subtype-level comparative analysis of ferredoxins between *Bacteroidetes*- and *Firmicutes*-species. Ferredoxins were classified into different subtypes following the procedure described elsewhere [1] 21

Table S4. Information on ferredoxins that are used as reference proteins for datamining of ferredoxins in *Bacteroidetes* species. The reference protein data is retrieved from the published article [1]. 23

Table S1. Information on *Bacteroidetes* species and their respective genera was used in the study. Species abbreviations, their genome IDs (GenBank) and presence or absence of P450s in different species, and the number of P450s were presented in the table.

**Genus**	**Number of species analyzed**	**Number of species with P450s**	**Number of species without P450s**	**Species code**	**Number of P450s**	**Species name**	**Genome ID**
Flavobacterium	20	1	19	fjo	1	*Flavobacterium johnsoniae* UW101	CP000685
				fjg	0	*Flavobacterium anhuiense*	CP016907
				fps	0	*Flavobacterium psychrophilum* JIP02/86	AM398681
				fpc	0	*Flavobacterium psychrophilum* CSF259-93	CP007627
				fpy	0	*Flavobacterium psychrophilum* FPG101	CP007206
				fpo	0	*Flavobacterium psychrophilum* FPG3	CP007207
				fpq	0	*Flavobacterium psychrophilum* 950106-1/1	CP008902
				fpv	0	*Flavobacterium psychrophilum* V3-5	CP008878
				fpw	0	*Flavobacterium psychrophilum* V4-24	CP008881
				fpk	0	*Flavobacterium psychrophilum* v4-33	CP008883
				fpsz	0	*Flavobacterium psychrophilum* Z2	CP012586
				fbr	0	*Flavobacterium branchiophilum*	FQ859183
				fco	0	*Flavobacterium columnare*	CP003222
				fin	0	*Flavobacterium indicum*	HE774682
				fgl	0	*Flavobacterium gilvum*	CP017479

				fcm	0	*Flavobacterium commune*	CP017774
				ffa	0	*Flavobacterium faecale*	CP020918
				fat	0	*Flavobacterium arcticum*	CP031188
				fki	0	*Flavobacterium kingsejongi*	CP020919
				fpal	0	*Flavobacterium pallidum*	CP029187
Bacteroides	19	0	19	bth	0	*Bacteroides thetaiotaomicron* VPI-5482	AE015928
				btho	0	*Bacteroides thetaiotaomicron* 7330	CP012937
				bfr	0	*Bacteroides fragilis* YCH46	AP006841
				bfs	0	*Bacteroides fragilis* NCTC9343	CR626927
				bfg	0	*Bacteroides fragilis* 638R	FQ312004
				bfb	0	*Bacteroides fragilis* BOB25	CP011073
				bvu	0	*Bacteroides vulgatus*	CP000139
				bhl	0	*Bacteroides helcogenes*	CP002352
				bsa	0	*Bacteroides salanitronis*	CP002530
				bxy	0	*Bacteroides xylanisolvens*	FP929033
				bdo	0	*Bacteroides dorei* HS1_L_1_B_010	CP007619
				bdh	0	*Bacteroides dorei* HS1_L_3_B_079	CP008741
				boa	0	*Bacteroides ovatus*	CP012938
				bcel	0	*Bacteroides cellulosilyticus*	CP012801
				bcac	0	*Bacteroides caccae*	CP022412
				bcae	0	*Bacteroides caecimuris*	CP015401
				bzg	0	*Bacteroides zoogleoformans*	CP027231
				bhf	0	*Bacteroides heparinolyticus*	CP027234
				bis	0	*Bacteroides intestinalis*	CP041379
Capnocytophaga	13	0	13	coc	0	*Capnocytophaga ochracea*	CP001632
				ccm	0	*Capnocytophaga canimorsus*	CP002113

				col	0	*Capnocytophaga* sp. oral taxon 323	CP012589
				chg	0	*Capnocytophaga haemolytica*	CP014227
				capn	0	*Capnocytophaga* sp. ChDC OS43	CP022022
				cgh	0	*Capnocytophaga gingivalis*	CP022386
				clk	0	*Capnocytophaga leadbetteri*	CP022384
				cspu	0	*Capnocytophaga sputigena*	CP022385
				ccyn	0	*Capnocytophaga cynodegmi*	CP022378
				caph	0	*Capnocytophaga* sp. H4358	CP022380
				csto	0	*Capnocytophaga stomatis*	CP022387
				capq	0	*Capnocytophaga* sp. H2931	CP022381
				capf	0	*Capnocytophaga* sp. FDAARGOS_737	CP046316
Chryseobacterium	13	1	12	chz	0	*Chryseobacterium* sp. StRB126	AP014624
				cgn	0	*Chryseobacterium gallinarum*	CP009928
				cih	0	*Chryseobacterium* sp. IHB B 17019	CP013293
				chh	0	*Chryseobacterium glaciei*	CP015199
				cio	0	*Chryseobacterium indologenes*	CP022058
				chry	0	*Chryseobacterium* sp. T16E-39	CP022282
				cpip	0	*Chryseobacterium piperi*	CP023049
				ctak	0	*Chryseobacterium taklimakanense*	LT906465
				chrs	1	*Chryseobacterium* sp. 3008163	CP033070
				chrz	0	*Chryseobacterium* sp. 6424	CP023540
				carh	0	*Chryseobacterium arthrosphaerae*	CP033811
				csha	0	*Chryseobacterium shandongense*	CP033914
				cnk	0	*Chryseobacterium nakagawai*	CP033923
Prevotella	10	0	10	pru	0	*Prevotella ruminicola*	CP002006
				pmz	0	*Prevotella melaninogenica*	CP002123

				pdn	0	*Prevotella denticola*	CP002589
				pit	0	*Prevotella intermedia*	CP003503
				pdt	0	*Prevotella dentalis*	CP003368
				pro	0	*Prevotella* sp. oral taxon 299	CP003666
				pfus	0	*Prevotella fusca*	CP012075
				peo	0	*Prevotella enoeca*	CP013195
				pje	0	*Prevotella jejuni*	CP023863
				poc	0	*Prevotella oris*	LR134384
Hymenobacter	10	8	2	hsw	0	*Hymenobacter swuensis*	CP007145
				hym	1	*Hymenobacter* sp. APR13	CP006587
				hyd	1	*Hymenobacter* sp. DG25B	CP010054
				hye	1	*Hymenobacter* sp. DG25A	CP012623
				hyg	1	*Hymenobacter sedentarius*	CP013909
				hyp	1	*Hymenobacter* sp. PAMC 26554	CP014771
				hyz	1	*Hymenobacter* sp. PAMC 26628	CP014304
				hnv	2	*Hymenobacter nivis*	CP029145
				hyh	1	*Hymenobacter* sp. sh-6	CP032317
				hyj	0	*Hymenobacter* sp. 17J68-5	CP040896
Sulcia	9	0	9	smg	0	*Candidatus Sulcia muelleri* GWSS	CP000770
				sms	0	*Candidatus Sulcia muelleri* SMDSEM	CP001605
				smh	0	*Candidatus Sulcia muelleri* DMIN	CP001981
				sum	0	*Candidatus Sulcia muelleri* CARI	CP002163
				smv	0	*Candidatus Sulcia muelleri Sulcia*-ALF	CP006060
				smub	0	*Candidatus Sulcia muelleri* BGSS	CP008986
				smum	0	*Candidatus Sulcia muelleri* ML	CP010105

				smue	0	*Candidatus Sulcia muelleri* TETUND	CP007234
				smup	0	*Candidatus Sulcia muelleri* PSPU	AP013293
Blattabacterium	9	0	9	bbl	0	*Blattabacterium* sp. *(Blattella germanica) Bge*	CP001487
				bpi	0	*Blattabacterium* sp. BPLAN *(Periplaneta americana)*	CP001429
				bmm	0	*Blattabacterium* sp. *(Mastotermes darwiniensis)*	CP003000
				bcp	0	*Blattabacterium Cpu (Cryptocercus punctulatus)*	CP003015
				bbg	0	*Blattabacterium* sp. *(Blaberus giganteus)*	CP003535
				bbq	0	*Blattabacterium* sp. *(Blatta orientalis)*	CP003605
				blp	0	*Blattabacterium cuenoti*	AP012548
				blu	0	*Blattabacterium* sp. *(Nauphoeta cinerea)*	CP005488
				blck	0	*Blattabacterium* sp. *(Cryptocercus kyebangensis)*	CP029820
Elizabethkingia	8	0	8	eao	0	*Elizabethkingia anophelis* NUHP1	CP007547
				emn	0	*Elizabethkingia anophelis* FMS-007	CP006576
				een	0	*Elizabethkingia anophelis* JM-87	CP016372
				elb	0	*Elizabethkingia miricola*	CP011059
				emg	0	*Elizabethkingia meningoseptica*	CP016376
				ego	0	*Elizabethkingia ursingii*	CP016377
				egm	0	*Elizabethkingia bruuniana*	CP014337
				elz	0	*Elizabethkingia* sp. 2-6	CP039929
Pedobacter	7	2	5	phe	0	*Pedobacter heparinus*	CP001681

				pep	1	*Pedobacter* sp. PACM 27299	CP012996
				pcm	2	*Pedobacter cryoconitis*	CP014504
				psty	0	*Pedobacter steynii*	CP017141
				pgs	0	*Pedobacter ginsengisoli*	CP024091
				pej	0	*Pedobacter* sp. CJ43	CP043329
				pek	0	*Pedobacter* sp. KBS0701	CP042171
Sphingobacterium	7	1	6	shg	1	*Sphingobacterium* sp. 21	CP002584
				sht	0	*Sphingobacterium* sp. ML3W	CP009278
				sphn	0	*Sphingobacterium* sp. B29	CP019158
				smiz	0	*Sphingobacterium mizutaii*	LT906468
				spsc	0	*Sphingobacterium psychroaquaticum*	CP038029
				sphz	0	*Sphingobacterium* sp. CZ-2	CP038159
				sphe	0	*Sphingobacterium* sp. dk4302	CP045652
Mucilaginibacter	7	0	7	mup	0	*Mucilaginibacter* sp. PAMC 26640	CP014773
				muc	0	*Mucilaginibacter* sp. BJC16-A31	CP022743
				mgot	0	*Mucilaginibacter gotjawali*	AP017313
				muh	0	*Mucilaginibacter* sp. HYN0043	CP032869
				mgin	0	*Mucilaginibacter ginsenosidivorans*	CP042436
				mgk	0	*Mucilaginibacter ginsenosidivorax*	CP042437
				mrub	0	*Mucilaginibacter rubeus*	CP043450
Riemerella	6	0	6	ran	0	*Riemerella anatipestifer* ATCC 11845 = DSM 15868	CP002346
				rai	0	*Riemerella anatipestifer* ATCC 11845 = DSM 15868	CP003388
				rar	0	*Riemerella anatipestifer* RA-GD	CP002562
				rag	0	*Riemerella anatipestifer* RA-CH-1	CP003787

				rae	0	*Riemerella anatipestifer* RA-CH-2	CP004020
				rat	0	*Riemerella anatipestifer* CH3	CP006649
Porphyromonas	5	0	5	pgi	0	*Porphyromonas gingivalis* W83	AE015924
				pgn	0	*Porphyromonas gingivalis* ATCC 33277	AP009380
				pgt	0	*Porphyromonas gingivalis* TDC60	AP012203
				pah	0	*Porphyromonas asaccharolytica*	CP002689
				pcre	0	*Porphyromonas crevioricanis*	LS483447
Spirosoma	5	4	1	sli	1	*Spirosoma linguale*	CP001769
				srd	1	*Spirosoma radiotolerans*	CP010429
				smon	1	*Spirosoma montaniterrae*	CP014263
				spir	1	*Spirosoma pollinicola*	CP025096
				spik	0	*Spirosoma* sp. KCTC 42546	CP041360
Maribacter	5	2	3	fbc	0	*Maribacter* sp. HTCC2170	CP002157
				marm	1	*Maribacter* sp. 1_2014MBL_MicDiv	CP011318
				mart	0	*Maribacter* sp. T28	CP018760
				marb	0	*Maribacter cobaltidurans*	CP022957
				mare	2	*Maribacter* sp. MJ134	CP034570
Cellulophaga	5	5	0	cao	2	*Cellulophaga algicola*	CP002453
				cly	1	*Cellulophaga lytica* DSM 7489	CP002534
				clh	1	*Cellulophaga lytica* HI1	CP009239
				cbal	2	*Cellulophaga baltica* NN016038	CP009887
				cbat	2	*Cellulophaga baltica* 18	CP009976
Nonlabens	5	4	1	ndo	1	*Nonlabens dokdonensis*	CP001397
				nom	1	*Nonlabens* sp. MIC269	CP011373
				nsd	0	*Nonlabens sediminis*	CP019342
				nob	1	*Nonlabens* sp. MB-3u-79	CP025116
				noj	1	*Nonlabens* sp. MJ115	CP034549

Polaribacter	5	1	4	pom	0	*Polaribacter* sp. MED152	CP004349
				pob	0	*Polaribacter vadi*	CP017477
				prn	1	*Polaribacter reichenbachii*	CP019419
				pola	0	*Polaribacter* sp. BM10	CP019704
				poa	0	*Polaribacter* sp. ALD11	CP025119
Tenacibaculum	5	1	4	tdi	0	*Tenacibaculum dicentrarchi*	CP013671
				ten	0	*Tenacibaculum* sp. LPB0136	CP018155
				tje	2	*Tenacibaculum jejuense*	LT899436
				tmar	0	*Tenacibaculum maritimum*	LT634361
				tmp	0	*Tenacibaculum mesophilum*	CP045192
Arachidicoccus	4	0	4	arb	0	*Arachidicoccus* sp. BS20	CP015971
				ark	0	*Arachidicoccus* sp. KIS59-12	CP032489
				agi	0	*Arachidicoccus ginsenosidivorans*	CP042434
				arac	0	*Arachidicoccus* sp. B3-10	CP044016
Gramella	4	4	0	gfo	1	*Gramella forsetii*	CU207366
				grl	1	*Gramella salexigens*	CP018153
				gfl	1	*Gramella flava*	CP016359
				grs	1	*Gramella fulva*	CP028136
Dokdonia	4	4	0	kdi	1	*Dokdonia* sp. 4H-3-7-5	CP002528
				dok	1	*Dokdonia* sp. MED134	CP009301
				ddo	1	*Dokdonia donghaensis*	CP015125
				dod	1	*Dokdonia* sp. Dokd-P16	CP029151
Myroides	4	0	4	myr	0	*Myroides* sp. A21	CP010327
				mpw	0	*Myroides profundi*	CP010817
				mod	0	*Myroides odoratimimus*	CP013690
				myz	0	*Myroides* sp. ZB35	CP017769
Alistipes	3	0	3	afd	0	*Alistipes finegoldii*	CP003274
				ash	0	*Alistipes shahii*	FP929032
				ald	0	*Alistipes* sp. dk3624	CP045651

Chitinophaga	3	1	2	cpi	2	*Chitinophaga pinensis*	CP001699
				cbae	0	*Chitinophaga caeni*	CP023777
				chit	0	*Chitinophaga* sp. XS-30	CP043006
Echinicola	3	0	3	evi	0	*Echinicola vietnamensis*	CP003346
				est	0	*Echinicola strongylocentroti*	CP030041
				echi	0	*Echinicola* sp. LN3S3	CP041253
Runella	3	3	0	rsi	2	*Runella slithyformis*	CP002859
				run	2	*Runella* sp. HYN0085	CP030850
				rup	1	*Runella* sp. SP2	CP031030
Rufibacter	3	0	3	ruf	0	*Rufibacter* sp. DG31D	CP010777
				rti	0	*Rufibacter tibetensis*	CP012643
				rud	0	*Rufibacter* sp. DG15C	CP010776
Cardinium	3	0	3	che	0	*Cardinium endosymbiont cEper1 (Encarsia pergandiella)*	HE983995
				cec	0	*Cardinium endosymbiont of Sogatella furcifera*	CP022339
				cher	0	*Candidatus Cardinium hertigii*	CP029619
Lacinutrix	3	0	3	lan	0	*Lacinutrix* sp. 5H-3-7-4	CP002825
				lvn	0	*Lacinutrix venerupis*	CP019352
				laci	0	*Lacinutrix* sp. Bg11-31	CP025118
Winogradskyella	2	0	2	win	0	*Winogradskyella* sp. PG-2	AP014583
				wij	0	*Winogradskyella* sp. J14-2	CP019388
Formosa	3	0	3	for	0	*Formosa* sp. Hel1_33_131	CP017260
				foh	0	*Formosa* sp. Hel3_A1_48	CP017259
				fop	0	*Formosa* sp. PS13	CP041637
Aquimarina	3	3	0	aqb	2	*Aquimarina* sp. BL5	CP031963
				aqa	2	*Aquimarina* sp. AD1	CP031966
				aqd	2	*Aquimarina* sp. AD10	CP031965

Unclassified Flavobacteriaceae	3	0	3	fba	0	*Flavobacteriaceae bacterium* 3519-10	CP001673
				fbu	0	*Flavobacteriaceae bacterium* UJ101	CP016269
				fbe	0	*Flavobacteriaceae bacterium* 10Alg115	CP040749
Petrimonas	2	0	2	pmuc	0	*Petrimonas mucosa*	LT608328
				pet	0	*Petrimonas* sp. IBARAKI	AP018040
					0		
Parabacteroides	2	0	2	pdi	0	*Parabacteroides distasonis*	CP000140
				parc	0	*Parabacteroides* sp. CT06	CP022754
Tannerella	2	0	2	tfo	0	*Tannerella forsythia*	CP003191
				toh	0	*Tannerella* sp. oral taxon HOT-286	CP017038
Salinibacter	2	0	2	sru	0	*Salinibacter ruber* DSM 13855	CP000159
				srm	0	*Salinibacter ruber* M8	FP565814
Rhodothermus	2	0	2	rmr	0	*Rhodothermus marinus* DSM 4252	CP001807
				rmg	0	*Rhodothermus marinus* SG0.5JP17-172	CP003029
Niabella	2	0	2	nso	0	*Niabella soli*	CP007035
				nia	0	*Niabella ginsenosidivorans*	CP015772
Flavisolibacter	2	0	2	fla	0	*Flavisolibacter tropicus*	CP011390
				fgg	0	*Flavisolibacter ginsenosidimutans*	CP042433
Cyclobacterium	2	0	2	cmr	0	*Cyclobacterium marinum*	CP002955
				camu	0	*Cyclobacterium amurskyense*	CP012040
Fibrella	2	2	0	fae	1	*Fibrella aestuarina*	HE796683
				fib	1	*Fibrella* sp. ES10-3-2-2	CP015317
Pontibacter	2	2	0	pko	1	*Pontibacter korlensis*	CP009621
				pact	2	*Pontibacter actiniarum*	CP021235

Flammeovirga	2	2	0	flm	2	*Flammeovirga* sp. MY04	CP003561
				fll	2	*Flammeovirga* sp. L12M1	CP034562
Muricauda	2	0	2	mrs	0	*Muricauda ruestringensis*	CP002999
				mlt	0	*Muricauda lutaonensis*	CP011071
Aequorivita	2	1	1	asl	1	*Aequorivita sublithincola*	CP003280
				aev	0	*Aequorivita* sp. H23M31	CP034951
Ornithobacterium	2	0	2	orh	0	*Ornithobacterium rhinotracheale* DSM 15997	CP003283
				ori	0	*Ornithobacterium rhinotracheale* ORT-UMN 88	CP006828
Olleya	2	2	0	oll	1	*Olleya* sp. Bg11-27	CP025117
				oaq	1	*Olleya aquimaris*	CP031612
Kordia	2	1	1	kos	1	*Kordia* sp. SMS9	CP031153
				kan	0	*Kordia antarctica*	CP019288
Antarcticibacterium	2	0	2	afla	0	*Antarcticibacterium flavum*	CP042476
				anp	0	*Antarcticibacterium* sp. PAMC 28998	CP042476
Fluviicola	2	0	2	fte	0	*Fluviicola taffensis*	CP002542
				flu	0	*Candidatus Fluviicola riflensis*	CP022585
Fermentimonas	1	0	1	pbt	0	*Fermentimonas caenicola*	LN515532
Paludibacter	1	0	1	ppn	0	*Paludibacter propionicigenes*	CP002345
Muribaculum	1	0	1	pary	0	*Muribaculum intestinale*	CP015402
Duncaniella	1	0	1	dun	0	*Duncaniella* sp. B8	CP040121
Barnesiella	1	0	1	bvs	0	*Barnesiella viscericola*	CP007034
Proteiniphilum	1	0	1	psac	0	*Proteiniphilum saccharofermentans*	LT605205
Odoribacter	1	0	1	osp	0	*Odoribacter splanchnicus*	CP002544
Butyricimonas	1	0	1	buy	0	*Butyricimonas* sp. H184	CP032819

Azobacteroides	1	0	1	aps	0	*Candidatus Azobacteroides pseudotrichonymphae (Coptotermes formosanus)*	AP010656
Alloprevotella	1	0	1	alq	0	*Alloprevotella* sp. E39	CP033459
Mucinivorans	1	0	1	rbc	0	*Mucinivorans hirudinis*	HG934468
Unclassified Bacteroidales	1	0	1	bacc	0	*Bacteroidales bacterium* CF	CP006772
Draconibacterium	1	0	1	dori	0	*Draconibacterium orientale*	CP007451
Salinivirga	1	0	1	blq	0	*Salinivirga cyanobacteriivorans*	CP013118
Alkalitalea	1	0	1	asx	0	*Alkalitalea saponilacus*	CP021904
Labilibaculum	1	0	1	mbas	0	*Labilibaculum antarcticum*	AP018042
Unclassified Rhodothermaceae	1	0	1	rbar	0	*Rhodothermaceae bacterium* RA	CP020382
Niastella	1	0	1	nko	0	*Niastella koreensis*	CP003178
Filimonas	1	1	0	fln	1	*Filimonas lacunae*	AP017422
Pseudoflavitalea	1	1	0	pseg	1	*Pseudoflavitalea* sp. 5GH32-13	CP032157
Panacibacter	1	0	1	pgin	0	*Panacibacter ginsenosidivorans*	CP042435
Pseudobacter	1	0	1	pgo	0	*Pseudobacter ginsenosidimutans*	CP042431
Flavihumibacter	1	0	1	fls	0	*Flavihumibacter* sp. SB-02	CP046566
Haliscomenobacter	1	0	1	hhy	0	*Haliscomenobacter hydrossis*	CP002691
Saprospira	1	1	0	sgn	1	*Saprospira grandis*	CP002831
Pseudopedobacter	1	0	1	psn	0	*Pseudopedobacter saltans*	CP002545
Solitalea	1	0	1	scn	0	*Solitalea canadensis*	CP003349
Anseongella	1	0	1	agd	0	*Anseongella ginsenosidimutans*	CP042432
Olivibacter	1	0	1	oli	0	*Olivibacter* sp. LS-1	CP041643
Unclassified Sphingobacteriaceae	1	0	1	sbx	0	*Sphingobacteriaceae bacterium* GW460-11-11-14-LB5	CP021237
Belliella	1	0	1	bbd	0	*Belliella baltica*	CP003281
Algoriphagus	1	0	1	alm	0	*Algoriphagus sanaruensis*	CP012836

Cytophaga	1	0	1	chu	0	*Cytophaga hutchinsonii*	CP000383
Dyadobacter	1	1	0	dfe	2	*Dyadobacter fermentans*	CP001619
Leadbetterella	1	0	1	lby	0	*Leadbetterella byssophila*	CP002305
Emticicia	1	1	0	eol	1	*Emticicia oligotrophica*	CP002961
Allopseudarcicella	1	1	0	psez	1	*Allopseudarcicella aquatilis*	CP029346
Arcticibacterium	1	1	0	als	1	*Arcticibacterium luteifluviistationis*	CP029480
Flexibacter	1	0	1	fli	0	*Bernardetia litoralis*	CP003345
Nibribacter	1	0	1	nib	0	*Nibribacter* sp. BT10	CP047897
Marivirga	1	0	1	mtt	0	*Marivirga tractuosa*	CP002349
Fabibacter	1	0	1	fpf	0	*Fabibacter pacificus*	CP028923
Unclassified Flammeovirgaceae	1	1	0	fbt	1	*Flammeovirgaceae bacterium* 311	CP004371
Amoebophilus	1	0	1	aas	0	*Candidatus Amoebophilus asiaticus*	CP001102
Chryseolinea	1	0	1	chk	0	*Chryseolinea* sp. KIS68-18	CP032382
Robiginitalea	1	0	1	rbi	0	*Robiginitalea biformata*	CP001712
Zunongwangia	1	1	0	zpr	3	*Zunongwangia profunda*	CP001650
Croceibacter	1	0	1	cat	0	*Croceibacter atlanticus*	CP002046
Weeksella	1	0	1	wvi	0	*Weeksella virosa*	CP002455
Zobellia	1	1	0	zga	1	*Zobellia galactanivorans*	FP476056
Psychroflexus	1	1	0	ptq	1	*Psychroflexus torquis*	CP003879
Siansivirga	1	0	1	sze	0	*Siansivirga zeaxanthinifaciens*	CP007202
Algibacter	1	0	1	ahz	0	*Algibacter alginicilyticus*	CP012898
Sediminicola	1	1	0	syi	1	*Sediminicola* sp. YIK13	CP010535
Lutibacter	1	0	1	lut	0	*Lutibacter profundi*	CP013355
Urechidicola	1	0	1	lul	0	*Urechidicola croceus*	CP017478
Wenyingzhuangia	1	1	0	wfu	1	*Wenyingzhuangia fucanilytica*	CP014224
Salegentibacter	1	1	0	salt	1	*Salegentibacter* sp. T436	CP012872

Seonamhaeicola	1	0	1	seon	0	*Seonamhaeicola* sp. S2-3	CP019389
Arenibacter	1	1	0	aalg	1	*Arenibacter algicola*	CP022515
Flavivirga	1	1	0	fek	3	*Flavivirga eckloniae*	CP025791
Tamlana	1	1	0	taj	1	*Tamlana* sp. UJ94	CP025938
Aureitalea	1	1	0	aue	1	*Aureitalea* sp. RR4-38	CP027062
Spongiibacterium	1	1	0	spon	1	*Flagellimonas* sp. HME9304	CP030104
Mariniflexile	1	0	1	marf	0	*Mariniflexile* sp. TRM1-10	CP022985
Euzebyella	1	1	0	emar	1	*Euzebyella marina*	CP032050
Cloacibacterium	1	0	1	cnr	0	*Cloacibacterium normanense*	CP034157
Muriicola	1	1	0	mur	1	*Muriicola* sp. MMS17-SY002	CP035544
Psychroserpens	1	0	1	psyn	0	*Psychroserpens* sp. NJDZ02	CP039451
Empedobacter	1	0	1	ebv	0	*Empedobacter brevis*	CP043634
Oceanihabitans	1	0	1	oci	0	*Oceanihabitans* sp. IOP_32	CP040813
Bergeyella	1	0	1	bcad	0	*Bergeyella cardium*	CP029149
Owenweeksia	1	1	0	oho	1	*Owenweeksia hongkongensis*	CP003156
Ichthyobacterium	1	0	1	ise	0	*Ichthyobacterium seriolicida*	AP014564
Walczuchella	1	0	1	elv	0	*Candidatus Walczuchella monophlebidarum*	CP006873
Uzinura	1	0	1	udi	0	*Candidatus Uzinura diaspidicola*	CP003263
Unclassified Bacteroidetes	1	0	1	bbau	0	*Bacteroidetes bacterium*	CP012155

Table S2. Comparative analysis of ferredoxins in *Bacteroidetes* species.

**Species name**	**Species code**	**Ferredoxin types**					
		**2Fe-2S**	**3Fe-4S**	**4Fe-4S**	**7Fe-8S**	**2[4Fe-4S]**	**2[4Fe-4S]Alv**
Zunongwangia profunda	zpr	1	0	0	0	0	1
Flavivirga eckloniae	fek	2	0	1	0	0	2
Chitinophaga pinensis	cpi	0	0	0	0	0	1
Pedobacter cryoconitis	pcm	2	0	0	0	0	1
Dyadobacter fermentans	dfe	1	0	0	0	0	1
Runella slithyformis	rsi	3	0	0	0	0	1
Runella sp. HYN0085	run	2	0	0	0	0	0
Hymenobacter nivis	hnv	2	0	0	0	0	2
Pontibacter actiniarum	pact	2	0	0	0	1	1
Flammeovirga sp. MY04	flm	1	0	1	0	1	1
Flammeovirga pectinis	fll	0	0	1	0	1	1
Maribacter sp. MJ134	mare	2	0	0	0	0	1
Cellulophaga algicola	cao	2	0	0	0	0	1
Cellulophaga baltica NN016038	cbal	1	0	0	0	0	1
Cellulophaga baltica 18	cbat	1	0	0	0	0	1
Tenacibaculum jejuense	tje	3	0	0	0	0	3
Aquimarina sp. BL5	aqb	2	1	0	0	0	1
Aquimarina sp. AD1	aqa	2	0	0	0	0	1
Aquimarina sp. AD10	aqd	2	0	0	0	0	1
Saprospira grandis	sgn	2	0	0	0	0	1
Filimonas lacunae	fln	2	0	0	0	0	1
Paraflavitalea soli	pseg	1	0	0	0	0	1
Pedobacter sp. PACM 27299	pep	2	0	0	0	0	1

Sphingobacterium sp. 21	shg	1	0	0	0	1	1
Spirosoma linguale	sli	1	0	0	0	0	1
Spirosoma radiotolerans	srd	1	0	0	0	0	1
Spirosoma montaniterrae	smon	0	0	0	0	0	1
Spirosoma pollinicola	spir	1	0	0	0	0	1
Runella sp. SP2	rup	1	0	0	0	0	1
Fibrella aestuarina	fae	0	0	0	0	0	1
Fibrella sp. ES10-3-2-2	fib	0	0	0	0	0	1
Emticicia oligotrophica	eol	1	0	1	0	0	1
Allopseudarcicella aquatilis	psez	0	0	0	0	0	0
Arcticibacterium luteifluviistationis	als	2	1	0	0	0	1
Hymenobacter sp. APR13	hym	1	0	0	0	0	1
Hymenobacter sp. DG25B	hyd	2	0	0	0	0	1
Hymenobacter sp. DG25A	hye	2	0	0	0	0	1
Hymenobacter sedentarius	hyg	2	0	0	0	0	1
Hymenobacter sp. PAMC 26554	hyp	2	0	0	0	0	1
Hymenobacter sp. PAMC 26628	hyz	2	0	0	0	0	1
Hymenobacter oligotrophus	hyh	3	0	0	0	0	1
Pontibacter korlensis	pko	2	0	0	0	1	1
Flammeovirgaceae bacterium 311	fbt	2	0	0	0	0	2
Gramella forsetii	gfo	1	0	0	0	0	1
Gramella salexigens	grl	1	0	0	0	0	1
Gramella flava	gfl	1	0	0	0	0	1
Gramella fulva	grs	1	0	1	0	0	2
Flavobacterium johnsoniae UW101	fjo	1	0	1	0	0	1
Maribacter sp. 1_2014MBL_MicDiv	marm	1	0	0	0	0	2
Cellulophaga lytica DSM 7489	cly	2	0	0	0	0	1
Cellulophaga lytica HI1	clh	2	0	0	0	0	1

Dokdonia sp. 4H-3-7-5	kdi	1	0	0	0	0	1
Dokdonia sp. MED134	dok	1	0	0	0	0	0
Dokdonia donghaensis	ddo	0	0	0	0	0	1
Dokdonia sp. Dokd-P16	dod	1	0	0	0	0	1
Zobellia galactanivorans	zga	3	0	0	0	0	1
Aequorivita sublithincola	asl	1	0	0	0	0	0
Psychroflexus torquis	ptq	1	0	0	0	0	1
Nonlabens dokdonensis	ndo	1	0	0	0	0	1
Nonlabens sp. MIC269	nom	1	0	0	0	0	1
Nonlabens sp. MB-3u-79	nob	1	0	0	0	0	2
Nonlabens ponticola	noj	1	0	0	0	0	1
Polaribacter reichenbachii	prn	2	0	1	0	0	1
Chryseobacterium sp. 3008163	chrs	1	1	0	0	0	1
Sediminicola sp. YIK13	syi	1	0	0	0	0	1
Wenyingzhuangia fucanilytica	wfu	1	0	0	0	0	1
Salegentibacter sp. T436	salt	1	0	0	0	0	1
Arenibacter algicola	aalg	0	1	0	0	0	1
Olleya sp. Bg11-27	oll	1	0	0	0	0	1
Olleya aquimaris	oaq	1	0	0	0	0	1
Tamlana carrageenivorans	taj	2	0	2	0	0	1
Pukyongia salina	aue	1	1	0	0	0	0
Flagellimonas sp. HME9304	spon	0	0	0	0	0	0
Kordia sp. SMS9	kos	0	1	0	0	0	1
Euzebyella marina	emar	1	0	0	0	0	1
Muriicola sp. MMS17-SY002	mur	1	0	0	0	0	1
Owenweeksia hongkongensis	oho	0	0	0	0	0	0
Aequorivita sublithincola QSSC9-3, DSM 14238		1	0	0	0	0	1

Cellulophaga algicola IC166, DSM 14237		2	0	0	0	0	1
Cellulophaga lytica DAU203		1	0	0	0	0	1
Cellulophaga lytica LIM-21, DSM 7489		1	0	0	0	0	1
Chitinophaga pinensis UQM 2034, DSM 2588		0	0	0	0	1	1
Dokdonia sp. PRO95		1	0	0	0	0	1
Dyadobacter fermentans NS114, DSM 18053		1	0	0	0	0	1
Emticicia oligotrophica GPTSA100-15, DSM 17448		1	0	0	0	0	1
Flavobacterium johnsoniae UW101, ATCC 17061		1	0	1	0	0	1
Gramella forsetii KT0803		1	0	0	0	0	1
Gramella sp. LPB0144		1	0	0	0	0	1
Haliscomenobacter hydrossis O, DSM 1100		2	0	0	0	0	1
Niastella koreensis GR20-10, DSM 17620		1	0	0	0	1	2
Nonlabens dokdonensis DSW-6		1	0	0	0	0	1
Nonlabens spongiae JCM 13191		1	0	0	0	0	1
Pedobacter heparinus HIM 762-3, DSM 2366		2	0	0	0	0	1
Polaribacter sp. SA4-12		2	0	1	0	0	1
Pontibacter korlensis X14-1T		3	0	0	0	1	1
Psychroflexus torquis ATCC 700755		1	0	0	0	0	1
Runella slithyformis LSU4, DSM 19594		2	0	0	0	0	1
Sphingobacterium sp. ML3W		1	0	0	0	1	1
Spirosoma linguale DSM 74		1	0	0	0	0	1
Spirosoma radiotolerans DG5A *(Spirosoma radiotolerans genome sequence)*		1	0	0	0	0	1

Tenacibaculum jejuense KCTC 22618		3	0	0	0	0	2
Wenyingzhuangia fucanilytica CZ1127		2	0	0	0	0	1
Zobellia galactanivorans DsijT		2	0	0	0	0	1
Zunongwangia profunda SM-A87		1	0	0	0	0	1
**104**		**136**	**6**	**11**	**0**	**9**	**107**

Note: Two Fe-S proteins were unable to assign to subtypes considering missing one of the typical cysteine amino acid. Experimenta evidence needed on Fe-S bining amino acids.

>*Hymenobacter oligotrophus* (D3Y59_10155)
MIYQHHLFVCTNQKSGVGSDVAKAIKKELKKQDLKKLLIEGKKRKNRVQTVGCLDVCKQCKKGHGAALVVYPEGVWYGNVHPRDAADIVHQHLGEGLAVGRLVIEK
>*Flavivirga eckloniae* (C1H87_05785)
MDIDAKEFRNSDITVTYDPCKCILSGICARELSDVFSNSIIPWVNLDNTETKRVIKQINRCPSGALKYHKNNEKKQAS

Table S3. Subtype-level comparative analysis of ferredoxins between *Bacteroidetes*- and *Firmicutes*-species. Ferredoxins were classified into different subtypes following the procedure described elsewhere [1]

Subtypes	Cysteine Spacing Signature	*Bacteroidetes*	*Firmicutes*
**2Fe-2S**			
Subtype 1	$CX_5CX_2CX_{36}C$	19	
Subtype 2	$CX_5CX_2CX_{37}C$	5	1
Subtype 3	$CX_4CX_2CX_{29}C$		1
Subtype 4	$CX_5CX_2CX_{35}C$	45	3
Subtype 5	$CX_5CX_2CX_{38}C$		1
Subtype 6	$CX_4CX_2CX_{34}C$		11
Subtype 8	$CX_4CX_2CX_{31}C$		6
Subtype 9	$CX_4CX_2CX_{33}C$		3
Subtype 18	$CX_5CX_2CX_{34}C$	66	20
Subtype 19	$CX_{10}CX_{31}CX_3C$	1	
Subtype 20	$CX_5CX_2CX_{32}C$		44
Subtype 21	$CX_4CX_{31}CX_3C$		5
Subtype 22	$CX_2CX_{41}CX_3C$		2
**3Fe-4S**			
Subtype 8	$CX_5CX_{32}CP$	6	
**4Fe-4S**			

Subtype 2	$CX_2CX_2CX_{43}CP$		107
Subtype 3	$CX_2CX_2CX_{45}CP$		24
Subtype 4	$CX_2CX_2CX_{37}CP$		6
Subtype 5	$CX_2CX_2CX_{44}CP$		2
Subtype 6	$CX_2CX_2CX_{39}CP$		1
Subtype 13	$CX_2CX_2CX_{48}CP$	7	
Subtype 14	$CX_2CX_2CX_{47}CP$	3	
Subtype 15	$CX_3CX_5CX_{32}CP$	1	
**7Fe-8S**			
Subtype 1	$CX_7CX_3CPX_{17}CX_2CX_2CX_3CP^*$		32
**2[4Fe-4S]**			
Subtype 3	$CX_2CX_2CX_3CX_{20}CX_2CX_2CX_3C$		2
Subtype 9	$CX_2CX_2CX_3CX_{18}CX_2CX_2CX_3C$		6
Subtype 12	$CX_2CX_2CX_3CX_{28}CX_2CX_2CX_3C$	3	
Subtype 15	$CX_2CX_2CX_3CX_{19}CX_2CX_2CX_3C$		4
Subtype 34	$CX_2CX_2CX_3CX_{38}CX_2CX_2CX_3C$	6	
**2[4Fe-4S]Alv**			
Subtype 11	$CX_2CX_2CX_3CX_{52}CX_2CX_8CX_3CX_3C$	80	
Subtype 12	$CX_2CX_2CX_3CX_{51}CX_2CX_8CX_3CX_3C$	27	

Table S4. Information on ferredoxins that are used as reference proteins for datamining of ferredoxins in *Bacteroidetes* species. The reference protein data is retrieved from the published article [1].

**2Fe-2S.**		
**GenBank Accession Number or PDB Code**	**Species Name**	**Reference**
NP_004100.1 (Adrenodoxin)	*Homo sapiens*	[2]
1PDX (Putidaredoxin)	*Pseudomonas putida*	[3]
ABB56370.1	*Synechococcus elongatus* PCC 7942 = FACHB-805	[4]
ABB56928.1	*Synechococcus elongatus* PCC 7942 = FACHB-805	[4]
WP_013424358.1 (FraEuI1c_3227)	*Frankia* sp. EuI1c (*Frankia inefficax* sp.)	[5]
WP_012394830.1 (mmi:MMAR_3155)	*Mycobacterium marinum*	[6]
**3Fe-4S/4Fe-4S**		
CAB59502.1	*Streptomyces coelicolor* A3(2)	[7]
WP_013425251.1 (FraEuI1c_4132)	*Frankia* sp. EuI1c (*Frankia inefficax* sp.)	[5]
WP_013426476.1 (FraEuI1c_5370)	*Frankia* sp. EuI1c (*Frankia inefficax* sp.)	[5]
WP_011740769.1 (Mmar_2879)	*Mycobacterium marinum*	[6]
WP_012395565.1 (Mmar_3973)	*Mycobacterium marinum*	[6]
WP_012396301.1 (Mmar_4763)	*Mycobacterium marinum*	[6]
NP_215277.1 (FdX-Rv0763c)	*Mycobacterium tuberculosis* H37Rv	[8, 9]
NP_216302.1 (FdxE-Rv1786)	*Mycobacterium tuberculosis* H37Rv	[8, 9]
ABB57779.1	*Synechococcus elongatus* PCC 7942 = FACHB-805	[4]
**7Fe-8S**		
NP_215693 (FdxC-Rv1177),	*Mycobacterium tuberculosis* H37Rv	[8]
NP_216523.1 (FdxA-Rv2007c)	*Mycobacterium tuberculosis* H37Rv	[8]
2VKR	*Acidianus ambivalens*	[10]
1H98	*Thermus thermophilus*	[11]
ABB56846.1	*Synechococcus elongatus* PCC 7942 = FACHB-805	[4]
**2[4Fe-4S]**		
2ZVS	*Escherichia coli* K-12	[12]
2FDN	*Clostridium acidurici*	[13]
WP_013068980.1 (FDI)	*Rhodobacter capsulatus*	[14]
**2[4Fe-4S]Alv**		
1BLU	*Allochromatium vinosum*	[15]
2FGO	*Pseudomonas aeruginosa*	[13]
WP_023923722.1 (FDIII)	*Rhodobacter capsulatus*	[14]
1RGV	*Thauera aromatica* K172	[16]

Note: For easy identification purpose for some ferredoxins their most popular literary names were included in parenthesis right after their GenBank accession number or PDB code.

References

1. Nzuza, N.; Padayachee, T.; Chen, W.; Gront, D.; Nelson, D. R.; Syed, K., Diversification of Ferredoxins across Living Organisms. *Current Issues in Molecular Biology* **2021,** 43, (3), 1374-1390.
2. Cai, K.; Tonelli, M.; Frederick, R. O.; Markley, J. L., Human mitochondrial ferredoxin 1 (FDX1) and ferredoxin 2 (FDX2) both bind cysteine desulfurase and donate electrons for iron–sulfur cluster biosynthesis. *Biochemistry* **2017,** 56, (3), 487-499.
3. Pochapsky, T. C.; Jain, N. U.; Kuti, M.; Lyons, T. A.; Heymont, J., A refined model for the solution structure of oxidized putidaredoxin. *Biochemistry* **1999,** 38, (15), 4681-4690.
4. Koksharova, O. A.; Klint, J.; Rasmussen, U., The first protein map of Synechococcus sp. strain PCC 7942. *Microbiology* **2006,** 75, (6), 664-672.
5. Lau, I. C.; Feyereisen, R.; Nelson, D. R.; Bell, S. G., Analysis and preliminary characterisation of the cytochrome P450 monooxygenases from Frankia sp. EuI1c (Frankia inefficax sp.). *Archives of biochemistry and biophysics* **2019,** 669, 11-21.
6. Child, S. A.; Bradley, J. M.; Pukala, T. L.; Svistunenko, D. A.; Le Brun, N. E.; Bell, S. G., Electron transfer ferredoxins with unusual cluster binding motifs support secondary metabolism in many bacteria. *Chemical science* **2018,** 9, (41), 7948-7957.
7. Bentley, S. D.; Chater, K. F.; Cerdeño-Tárraga, A.-M.; Challis, G. L.; Thomson, N.; James, K. D.; Harris, D. E.; Quail, M. A.; Kieser, H.; Harper, D., Complete genome sequence of the model actinomycete Streptomyces coelicolor A3 (2). *Nature* **2002,** 417, (6885), 141.
8. Ortega Ugalde, S.; de Koning, C. P.; Wallraven, K.; Bruyneel, B.; Vermeulen, N. P. E.; Grossmann, T. N.; Bitter, W.; Commandeur, J. N. M.; Vos, J. C., Linking cytochrome P450 enzymes from Mycobacterium tuberculosis to their cognate ferredoxin partners. *Applied microbiology and biotechnology* **2018,** 102, (21), 9231-9242.
9. McLean, K. J.; Warman, A. J.; Seward, H. E.; Marshall, K. R.; Girvan, H. M.; Cheesman, M. R.; Waterman, M. R.; Munro, A. W., Biophysical characterization of the sterol demethylase P450 from Mycobacterium tuberculosis, its cognate ferredoxin, and their interactions. *Biochemistry* **2006,** 45, (27), 8427-43.
10. Frazão, C.; Aragão, D.; Coelho, R.; Leal, S. S.; Gomes, C. M.; Teixeira, M.; Carrondo, M. A., Crystallographic analysis of the intact metal centres [3Fe–4S] 1+/0 and [4Fe–4S] 2+/1+ in a Zn2+-containing ferredoxin. *FEBS letters* **2008,** 582, (5), 763-767.
11. Macedo-Ribeiro, S.; Martins, B. M.; Pereira, P. B.; Buse, G.; Huber, R.; Soulimane, T., New insights into the thermostability of bacterial ferredoxins: high-resolution crystal structure of the seven-iron ferredoxin from Thermus thermophilus. *JBIC Journal of Biological Inorganic Chemistry* **2001,** 6, (7), 663-674.
12. Saridakis, E.; Giastas, P.; Efthymiou, G.; Thoma, V.; Moulis, J.-M.; Kyritsis, P.; Mavridis, I. M., Insight into the protein and solvent contributions to the reduction potentials of [4Fe–4S] 2+/+ clusters: crystal structures of the Allochromatium vinosum ferredoxin variants C57A and V13G and the homologous Escherichia coli ferredoxin. *JBIC Journal of Biological Inorganic Chemistry* **2009,** 14, (5), 783-799.
13. Dauter, Z.; Wilson, K. S.; Sieker, L. C.; Meyer, J.; Moulis, J.-M., Atomic resolution (0.94 Å) structure of Clostridium acidurici ferredoxin. Detailed geometry of [4Fe-4S] clusters in a protein. *Biochemistry* **1997,** 36, (51), 16065-16073.

14. Saeki, K.; Suetsugu, Y.; Tokuda, K.-I.; Miyatake, Y.; Young, D.; Marrs, B.; Matsubara, H., Genetic analysis of functional differences among distinct ferredoxins in Rhodobacter capsulatus. *Journal of Biological Chemistry* **1991,** 266, (20), 12889-12895.
15. Moulis, J. M.; Sieker, L. C.; Wilson, K. S.; Dauter, Z., Crystal structure of the 2 [4Fe‐4S] ferredoxin from Chromatium vinosum: Evolutionary and mechanistic inferences for [3/4Fe‐4S] ferredoxins. *Protein science* **1996,** 5, (9), 1765-1775.
16. Unciuleac, M.; Boll, M.; Warkentin, E.; Ermler, U., Crystallization of 4-hydroxybenzoyl-CoA reductase and the structure of its electron donor ferredoxin. *Acta Crystallographica Section D: Biological Crystallography* **2004,** 60, (2), 388-391.
